# Noise-induced shallow circuits and the absence of barren plateaus

**DOI:** 10.1038/s41567-026-03245-z

**Published:** 2026-04-02

**Authors:** Antonio Anna Mele, Armando Angrisani, Soumik Ghosh, Sumeet Khatri, Jens Eisert, Daniel Stilck França, Yihui Quek

**Affiliations:** 1 Dahlem Center for Complex Quantum Systems, Freie Universität Berlin, Berlin, Germany; 2 LIP6, CNRS, Sorbonne Université, Paris, France; 3 Institute of Physics, Ecole Polytechnique Fédérale de Lausanne, Lausanne, Switzerland; 4 Department of Computer Science, University of Chicago, Chicago, IL USA; 5 Fraunhofer Heinrich Hertz Institute, Berlin, Germany; 6 Department of Mathematical Sciences, University of Copenhagen, Copenhagen, Denmark; 7 Univ Lyon, ENS Lyon, UCBL, CNRS, Inria, LIP, Lyon, France; 8 Departments of Mathematics and Physics, Massachusetts Institute of Technology, Cambridge, MA USA

**Keywords:** Quantum information, Qubits

## Abstract

Without a successful implementation of fault-tolerant quantum error correction, calculations on quantum computers are subject to noise that limits their capabilities. Here, motivated by realistic near-term hardware considerations, we study the impact of uncorrected local noise on logical quantum circuits. We first show that, in the task of estimating observable expectation values, any noise effectively truncates most quantum circuits to logarithmic depth. We then prove that quantum circuits under any non-unital noise do not exhibit barren plateaus for cost functions composed of local observables. However, by using the effective shallowness, we also design an efficient classical algorithm to estimate observable expectation values within any constant additive accuracy, with high probability over the choice of the circuit, in any circuit architecture. Taken together, our results establish that, unless we carefully engineer quantum circuits to take advantage of the noise, noisy quantum circuits are unlikely to offer an advantage over shallow ones for algorithms that output observable expectation value estimates, such as many variational quantum machine learning proposals.

## Abstract (web summary)

Calculations on near-term quantum computers will be limited by the effects of noise. It has now been shown how different kinds of noise limit the achievable computational advantage of many proposed quantum algorithms.

## Main

Understanding the impact of noise is one of the central questions for today’s quantum computers^1^. A key issue is whether noisy devices can already provide an advantage—either for practically relevant problems^2–5^ or as proof-of-principle demonstrations^6^—or whether error-corrected logical qubits are ultimately required^4,7^. Recent years have seen a tussle between demonstrations of quantum advantage^3,6,8–10^ and subsequent efficient classical simulation^11–20^.

Noise plays a multifaceted role across near-term quantum computing. In quantum machine learning, certain noise models can induce barren plateaus, flattening optimization landscapes and suppressing quantum signals^21,22^. In random circuit sampling^10^, noise can render the dynamics efficiently classically simulable^23^. Yet, most prior work assumes local, unital and primitive noise (for example, depolarizing), whereas in many physical platforms it is more realistic to consider non-unital noise^6,24–26^, which can decrease entropy and make depolarizing models misleading, as already observed in fault tolerance^27^ and random circuit sampling^28^.

In this work, we develop a unified picture of how possibly non-unital noise affects typical quantum circuits. We assume only that the noise is local and incoherent, meaning it has a tensor-product structure and is non-unitary. Our main results provide guidance on the impact of quantum noise and are as follows.

## Effective depth

We show that arbitrarily deep random quantum circuits, under any uncorrected, possibly non-unital noise, effectively get ‘truncated’: the influence of gates on observable expectation values decreases exponentially with their distance from the last layer, so only the last layers contribute significantly. In particular, for typical circuits this implies that, within the task of estimating expectation values, non-unital and unital noise lead to essentially the same effective complexity. It is well known that, under certain unital noise models, such as depolarizing noise, meaningful computation must be confined to logarithmic depth^29^. We prove that the same logarithmic-depth limitation holds for typical circuits under arbitrary local noise—even non-unital: all but the last $\log n$ layers can be discarded without affecting predicted expectation values.

## Lack of barren plateaus

Under non-unital noise, we get a provable lack of barren plateaus for cost functions made out of local observables—that is, the cost landscape is never flat and gradients do not vanish—at any depth. This also implies that local expectation values of arbitrary deep random circuits with non-unital noise are not too concentrated towards a fixed value, in stark contrast to the unital-noise scenario^21^. This phenomenon, however, is not good news for variational quantum algorithms^22^, as we show that such circuits behave like shallow circuits, which have limited computational power.

## Classical simulation

Furthermore, exploiting this effective shallowness, we show how to efficiently classically simulate—on average over the circuit ensemble—expectation values of any local observable up to constant additive precision, at any depth and in any circuit architecture.

In this work, we focus on the problem of estimating expectation values of observables, rather than sampling tasks. This focus is motivated by two considerations. First, in condensed-matter physics and quantum simulation, the physically relevant quantities that diagnose phases of matter, reveal order parameters or determine response functions are expectation values of local or few-body observables. Second, in variational quantum algorithms and quantum machine learning, the central task is to estimate cost functions made by observables expectation values on noisy near-term devices. For these reasons, expectation values are the natural figure of merit for the questions we target, and form the basis of our analysis.

In summary, our results show that most quantum circuits with non-unital noise at any depth behave qualitatively as (noisy) shallow circuits for estimating observable expectation values. Beyond this task, we further establish that the majority of noisy quantum circuits *Φ* with depth at least linear in the number of qubits become independent of the initial state: for any two states *ρ* and *σ*, the trace distance between *Φ*(*ρ*) and *Φ*(*σ*) vanishes exponentially in the number of qubits. Although our noise model is significantly more general than in much prior work, our results hold only on average over a well-motivated class of circuits and do not apply to every circuit. However, this limitation is necessary; specifically, it reflects the fact that not every quantum circuit without access to fresh auxiliary systems becomes computationally trivial after a certain number of operations under more general noise, unlike circuits subjected to depolarizing noise^30^. For instance, ref. ^27^ has shown that it is possible to perform exponentially long quantum computations under non-unital noise, with specially constructed circuits. Because of this, we cannot expect to prove our statements for all quantum circuits with non-unital noise.

From a technical perspective, our results rely on bounding various second moments of observable expectation values under noisy random quantum circuits. In particular, we show how combining a normal form of qubit channels^31^ with a reduction to ensembles of random Clifford circuits renders most computations tractable. The only assumption we require is satisfied for any architecture in which the local gates form 2-designs^32,33^, making our results widely applicable.

Taken together, our findings substantially advance the understanding of noise in near-term quantum computation and indicate that, unless circuits are carefully engineered to exploit non-unital effects (for example, as in ref. ^27^), a quantum computer with non-unital noise is unlikely to outperform one with depolarizing noise.

### Noise-induced effective shallow circuits

We now present our main results on the effective depth of noisy random quantum circuits with respect to estimating observable expectation values. Our findings show that the influence of gates decays exponentially with their distance from the final layer. We formalize this phenomenon as follows.

#### Theorem 1 (effective logarithmic depth)

*Let O* *be an observable*, *ρ*_0_ *an arbitrary initial state, L the circuit depth and* $m\in {\mathbb{N}}$. *Assume the noise is local and decomposes into single-qubit non-unitary channels*. *Then*

1 $${{\mathbb{E}}}_{{\varPhi }_{[L-m,L]}}| {\rm{T}}{\rm{r}}\,(O\varPhi ({\rho }_{0}))-{\rm{T}}{\rm{r}}\,(O{\varPhi }_{[L-m,L]}({\sigma }_{0}))| \le \parallel O{\parallel }_{\infty }{{\rm{e}}}^{-\alpha m}.$$

*Here*, *σ*_0_ *is any fixed reference state and* *α* > 0 *depends only on the noise parameters, and* *Φ*_[*L*−*m*, *L*]_ *denotes the noisy circuit obtained by restricting* *Φ* *to its last* *m* *layers, that is, discarding all gates and noise operations preceding layer* *L* − *m*. *The expectation* ${{\mathbb{E}}}_{{\varPhi }_{[L-m,L]}}$ *is* *taken over the randomness of the two-qubit gates in these last* *m* *layers, assumed to form a local* 2-*design*.

Thus, with high probability over the circuit ensemble, only the last $\varTheta (\log n)$ layers influence observable expectation values up to inverse-polynomial precision. In this sense, deep noisy random circuits behave effectively as shallow ones. The previous theorem actually follows from a stronger second-moment estimate.

#### Theorem 2

*For general scaling, let* *ρ*, *σ* *be arbitrary states and* *P* ∈ {*I*, *X*, *Y*, *Z*}^⊗*n*^ *a Pauli operator of weight* ∣*P*∣. *For circuit depth* *m*

2 $${{\mathbb{E}}}_{\varPhi }[{({\rm{T}}{\rm{r}}(P\varPhi (\rho -\sigma )))}^{2}]\le {{\rm{e}}}^{-\varOmega (m+| P| )}.$$

The proof proceeds in the Heisenberg picture by iteratively applying adjoint noisy layers to *P*, showing that Pauli expectations contract exponentially in depth. By Jensen’s inequality

3 $${{\mathbb{E}}}_{{\Phi }}[| {\rm{T}}{\rm{r}}(P\varPhi (\rho ))-{\rm{T}}{\rm{r}}(P\varPhi (\sigma ))| ]\le {{\rm{e}}}^{-\varOmega (m+| P| )}.$$

As a consequence, for linear depth *m* = *Ω*(*n*)

4 $${{\mathbb{E}}}_{\varPhi }[\parallel \varPhi (\rho )-\varPhi (\sigma ){\parallel }_{1}]\le {{\rm{e}}}^{-\varOmega (m)}.$$

This implies that the application of the same linear depth random circuit affected by any amount of noise on two different input states renders them effectively indistinguishable (because of the Holevo–Helstrom theorem^34^). To our knowledge, this kind of result was not known before; except for the result of ref. ^35^, which applies only to exponential depths but holds for worst-case circuits, whereas our statement holds on average. We remark that it is in principle not possible to prove our result for worst-case non-unital noisy circuits, because there are some special classes of circuits^27^ that would violate a worst-case version of our inequality (that is, equation (4) without the expectation value). However, in a sufficiently high-noise regime, we can also prove a worst-case contraction bound in trace distance (that is, without averaging over circuits). Concretely, there exists an explicit constant $b=b({\mathcal{N}})\in (0,1)$, depending only on the single-qubit noise channel ${\mathcal{N}}$, such that for any depth-*m* noisy circuit *Φ* and any two states *ρ*, *σ*, we find

5 $$\parallel \varPhi (\rho )-\varPhi (\sigma ){\parallel }_{1}\,\le \,n\,{b}^{m}\,\parallel \rho -\sigma {\parallel }_{1}.$$

In particular, for any *ε* > 0, if $m=\varOmega (\log (n/\varepsilon ))$, then ∥*Φ*(*ρ*) − *Φ*(*σ*)∥_1 _≤ *ε*.

The proof relies on contraction properties of the quantum Wasserstein distance of order 1 (ref. ^36^). Bounds of this form are often referred to as reverse threshold theorems, as they show that, above a certain noise level, long computations (and, hence, error correction) become impossible^37–39^. Here, we extend this type of statement to non-unital noise.

### Classical simulation of random quantum circuits with possibly non-unital noise

We consider the classical task of estimating expectation values produced by noisy random quantum circuits. Given a depth-*L* noisy circuit instance *Φ*, whose two-qubit gates are sampled uniformly at random from a fixed architecture, together with an initial state *ρ*_0_ and an observable *O*, the goal is to estimate ${\rm{T}}{\rm{r}}(O\varPhi ({\rho }_{0}))$ to additive accuracy *ε* with high probability over the choice of *Φ*. We assume that *O* is a linear combination of *M* = poly(*n*) local Pauli operators. Because the runtime scales linearly in *M*, it suffices to treat the case *M* = 1, that is, estimating the expectation value of a single local Pauli operator *P*. High-Pauli-weight components are exponentially suppressed and can be handled separately.

The effective-depth picture yields an average-case truncation guarantee. Let

6 $$C(\varPhi ):={\rm{T}}{\rm{r}}\,(P\,\varPhi ({\rho }_{0})),$$

7 $${C}_{m}(\varPhi ):={\rm{T}}{\rm{r}}\,(P\,{\varPhi }_{[L-m,L]}(| {0}^{n}\rangle \langle {0}^{n}| {0}^{n})),$$

where *Φ*_[*L*−*m*, *L*]_ denotes the noisy circuit obtained by restricting *Φ* to its last *m* layers. Then, theorem 2 implies

$${{\mathbb{E}}}_{{\varPhi }_{[L-m,L]}}[| C(\varPhi )-{C}_{m}(\varPhi )| ]\le \rm{e}^{-\varOmega (m+| P| )}.$$

This suggests the following estimator. In the Heisenberg picture, compute ${P}_{m}:={\varPhi }_{[L-m,L]}^{* }(P)$, and output

8 $$\widehat{C}:=Tr\,({P}_{m}| {0}^{n}\rangle \langle {0}^{n}| {0}^{n}).$$

The runtime is governed by the size of the light cone of *P* under ${\varPhi }_{[L-m,L]}^{* }$.

#### Proposition 3

*For the average classical simulation of local expectation values, let* *ε*, *δ* > 0. *Fix a local Pauli operator* *P* *and an arbitrary initial state* *ρ*_0_. *Let* *Φ* *be a noisy circuit of depth* *L* *drawn from the described random-circuit ensemble*. *Then there exists a classical algorithm that outputs a value* $\widehat{C}$ *such that*

9 $$| \widehat{C}-{\rm{T}}{\rm{r}}(P\varPhi ({\rho }_{0}))| \le \varepsilon$$

*with probability at least* 1 − *δ* *over the choice of the random circuit*. *One valid output is the estimator* (8) *with*

10 $$m=\left\lceil \frac{1}{\log ({c}^{-1})}\log \,\left(\frac{4}{{\rm{\delta }}{\varepsilon }^{2}}\right)\right\rceil,$$

*where* *δ* *denotes the allowed failure probability, so that the estimator succeeds with probability at least* 1 − *δ* *and* *c* < 1 *is a noise parameter* (*defined in ‘Preliminaries and definitions’ in* Methods). *The runtime satisfies* $\exp \,(O({m}^{D}))$ *for D-dimensional geometrically local architectures and* $\exp \,(\exp (O(m)))$ *for all-to-all connected architectures*.

The runtime bounds follow from evaluating $\widehat{C}=Tr({P}_{m}| {0}^{n}\rangle \langle {0}^{n}| {0}^{n})$, where ${P}_{m}={\varPhi }_{[L-m,L]}^{* }(P)$ is supported on the light cone of *P*. In *D*-dimensional geometrically local architectures, this support has size *O*(*m*^*D*^), while in all-to-all architectures it is at most 2^*m*^. For constant accuracy *ε* = *O*(1) (and *δ* = *O*(1)) the algorithm is efficient in any architecture. For inverse-polynomial accuracy, the runtime is polynomial in one dimension and quasi-polynomial in higher constant dimensions.

We additionally provide a verification (early-break) condition. Let ${P}_{t}:={\varPhi }_{[L-t,L]}^{* }(P).$ If

11 $$\mathop{\min }\limits_{c\in {\mathbb{R}}}\,\parallel {P}_{t}-c\,I{\parallel }_{\infty }\le \varepsilon /2,$$

where *I* denotes the identity operator, then $Tr({P}_{t}| {0}^{n}\rangle \langle {0}^{n}| {0}^{n})$ is guaranteed to be *ε*-accurate. Equation (11) can be checked within the same asymptotic runtime as above.

When the Pauli weight ∣*P*∣ is large, no simulation is necessary. For any non-unitary noise channel

12 $${{\mathbb{E}}}_{\Phi }\,[{\rm{T}}{\rm{r}}(P\varPhi ({\rho }_{0}))]=0,$$

13 $${\rm{V}}{\rm{a}}{{\rm{r}}}_{\varPhi }\,({\rm{T}}{\rm{r}}(P\varPhi ({\rho }_{0})))\le \rm{e}^{-\varOmega (| P| )},$$

and Chebyshev’s inequality implies that outputting 0 achieves inverse-polynomial accuracy with high probability once ∣*P*∣ is sufficiently large. Thus, for any constant target accuracy and any architecture, there exists an efficient classical algorithm for estimating Pauli expectation values of noisy random circuits at any depth. The method extends to any observable with bounded operator norm that can be expressed as a linear combination of polynomially many Pauli operators.

We now state the performance guarantee of an improved algorithm that reduces the runtime in the regime of inverse-polynomial accuracy. In addition to truncating the depth to the last *m* layers, this algorithm further restricts the simulation to Pauli strings of small weight within the light cone, discarding high-weight components using the exponential suppression established earlier.

#### Theorem 4

*For the improved classical algorithm*, *let* *Φ* *be sampled uniformly at random from a fixed architecture with arbitrary depth and any local non-unitary noise*. *For any constant-weight Pauli operator* *P* *and any initial state* *ρ*_0_, *the expectation value* ${\rm{T}}{\rm{r}}(P\varPhi ({\rho }_{0}))$ *can be estimated to accuracy* *ε* = poly(*n*^−1^) *with high probability*. *The runtime is* ${n}^{O((D-1)\log \log n)}poly(n)$ *for D-dimensional geometrically local architectures and* ${n}^{O(\log n)}$ *for all-to-all architectures*.

The proof of correctness of this algorithm follows from combining the effective-depth and Pauli-weight suppression results developed in this work with the techniques from ref. ^40^, originally introduced for noiseless random circuits, which we extend to the noisy setting.

### Lack of barren plateaus with non-unital noise, but only the last ${\pmb{\varTheta}} {\bf{(}}{\pmb{\log}} {\boldsymbol{n)}}$ layers matter

The barren-plateau phenomenon^21,41^ is widely viewed as a main hurdle for variational quantum algorithms^22^, where one encodes the solution to a problem in the minimization of a cost function, typically given by expectation values of observables, and optimizes over gate parameters. In barren-plateau regimes, a randomly chosen circuit instance lies with overwhelming probability in a nearly flat region of the landscape, so that extracting gradient information requires an exponential number of circuit evaluations, precluding any advantage.

Two standard signatures of barren plateaus are: (1) exponential concentration of the cost around a fixed value, and (2) exponentially small gradients. We show that both signatures are avoided under non-unital noise for cost functions built from local observables, in stark contrast to the noiseless^41^ and unital-noise^21^ settings. At the same time, we will see that this absence of barren plateaus is entirely driven by the final $\varTheta (\log n)$ layers: gradient contributions from earlier layers are negligible, reflecting the effective logarithmic depth established in ‘Noise-induced effective shallow circuits’.

#### Local costs do not concentrate under non-unital noise

We exploit that two-qubit gates are drawn from a 2-design, and we consider up to second-moment quantities. In this setting, composing the single-qubit noise channel ${\mathcal{N}}$ on the left and right by unitary channels does not affect the relevant distributions. We may therefore invoke the standard normal-form representation^31,42^, writing the action of ${\mathcal{N}}$ on the Bloch ball as

14 $${\mathcal{N}}\,\left(\frac{I+{\bf{w}}\cdot {\bf{\upsigma }}}{2}\right)=\frac{I}{2}+\frac{1}{2}({\bf{t}}+D{\bf{w}})\cdot {\bf{\upsigma }},$$

where **σ** = (*X*, *Y*, *Z*), ${\bf{w}}\in {{\mathbb{R}}}^{3}$ with ∥**w**∥_2 _≤ 1, ${\bf{t}}=({t}_{X},{t}_{Y},{t}_{Z})\in {{\mathbb{R}}}^{3}$ is the translation vector (quantifying non-unitality), and *D* = diag(**D**) with **D** = (*D*_*X*_, *D*_*Y*_, *D*_*Z*_). We consider circuit architectures as in ‘Preliminaries and definitions’ in Methods, where the local noise channels are characterized by constant parameters **t**, **D** (independent of *n*). Our first theorem states that under non-unital noise the variance of observable expectation values is depth independent.

#### Theorem 5

*For the variance of expectation values of random circuits with non-unital noise*, *let* $H:={\sum }_{P\in {\{I,X,Y,Z\}}^{\otimes n}}{a}_{P}P$ *be an arbitrary Hamiltonian with* ${a}_{P}\in {\mathbb{R}}$, *and let* *ρ* *be any quantum state*. *Assume the noise is non-unital and* ∥**t**∥_2_ = *Θ*(1). *Then, at any depth*

15 $${\rm{V}}{\rm{a}}{{\rm{r}}}_{\varPhi }\,[{\rm{T}}{\rm{r}}(H\,\varPhi (\rho ))]=\mathop{\sum }\limits_{P\in {\{I,X,Y,Z\}}^{\otimes n}\backslash {I}^{\otimes n}}{a}_{P}^{2}\,\rm{e} \,^{-\Theta (| P| )}.$$

This contrasts sharply with noiseless random circuits and circuits with unital noise^21,43^, where the variance becomes exponentially small in *n* at sufficiently large depth. In particular, theorem 5 implies that local cost functions have macroscopically large variance at any depth.

#### Corollary 6

*For the lack of exponential concentration for local cost functions*, *let* *ρ* *be any quantum state, let* *P* *be a local Pauli* (∣*P*∣ = *Θ*(1)) *and let* *Φ* *be the noisy random-circuit ansatz*. *Under the assumptions of theorem* 5, *at any depth*

16 $${\rm{V}}{\rm{a}}{{\rm{r}}}_{\varPhi }\,[{\rm{T}}{\rm{r}}(P\,\varPhi (\rho ))]=\varTheta (1).$$

In particular, the lower bound we prove is $\frac{1}{3}\parallel {\bf{t}}{\parallel }_{2}^{2| P| }$. As expected, in the unital limit ∥**t**∥_2_ = 0, this becomes vacuous. Notably, even a tiny deviation from unitality—for example, ∥**t**∥_2_ inverse-polynomial in *n*—already prevents the variance of local observables from being exponentially small. Thus local expectation values under non-unital noise are not overly concentrated around their mean ${{\mathbb{E}}}_{\varPhi }[{\rm{T}}{\rm{r}}(P\,\varPhi (\rho ))]=0$, unlike the depolarizing-noise scenario where the variance decays exponentially with depth^21^.

By contrast, global cost functions still exhibit exponential concentration, as in the noiseless case^43,44^ and under depolarizing noise.

#### Corollary 7

For cost concentration for global expectation values, let *P* ∈ {*I*, *X*, *Y*, *Z*}^⊗*n*^ have Pauli weight ∣*P*∣ = *Θ*(*n*). Assuming the noise is not a unitary channel and the noise parameters are constant, we have

17 $${\rm{V}}{\rm{a}}{{\rm{r}}}_{\varPhi }\,[{\rm{T}}{\rm{r}}(P\,\varPhi (\rho ))]\le \rm{e}^{-\varOmega (n)}.$$

#### Gradients do not vanish, but only the last ${\pmb{\varTheta}} {\bf{(}}{\pmb{\log}} {\boldsymbol{n)}}$ layers are trainable

The effective-depth picture from ‘Noise-induced effective shallow circuits’ implies that, on average, changing gates in layers preceding the last $\varTheta (\log n)$ has negligible effect on local expectation values. We formalize this via the standard notion of trainability^22,45^: a cost *C* is trainable with respect to a parameter *μ* if Var[∂_*μ*_*C*] = *Ω*(1/poly(*n*)). We show that, for local cost functions under non-unital noise, only parameters in the final $\varTheta (\log n)$ layers are trainable.

#### Theorem 8

*(Only the last few layers are trainable for local cost functions.)* *Let* $C=Tr(P\,\varPhi ({\rho }_{0}))$ *be a cost function associated with a local Pauli* *P* (∣*P*∣ = *Θ*(1)), *an initial state* *ρ*_0_, *and a depth*-*L* *noisy random circuit ansatz* *Φ* *in arbitrary dimension*. *Let* *μ* *be a parameter* (*in the light cone*) *of the kth layer*. *Assume the noise is non-unital and not a replacer channel* (*that is, it does not output a fixed state*). *Then*

18 $${\rm{V}}{\rm{a}}{\rm{r}}[{\partial }_{\mu }C]=\exp \,(-\varTheta (L-k)).$$

Theorem 8 shows that local cost functions are sensitive primarily to parameters in the final layers. This is in stark contrast to the depolarizing-noise and noiseless settings, where partial-derivative variances across all layers are exponentially suppressed in *n* at sufficiently large depth^21,41,43^.

As a direct consequence, the gradient of local cost functions does not vanish exponentially in the number of qubits at any depth; crucially, this is entirely due to the final trainable layers.

#### Corollary 9

*For non-vanishing gradient for local cost functions with non-unital noise*, *let* *ρ*_0_ *be any quantum state and* *let* *P* *be a local Pauli*. *Let* $C:={\rm{T}}{\rm{r}}(P\,\varPhi ({\rho }_{0}))$ *be the cost function associated with a noisy random circuit ansatz of arbitrary depth*. *Assume the noise is non-unital and not a replacer channel*. *Then*

19 $${\mathbb{E}}\,\left[\parallel \nabla C{\parallel }_{2}^{2}\right]=\varTheta (1).$$

We also show that global cost functions (associated with high-weight Pauli observables) have exponentially small partial-derivative variances, and in Supplementary Information section V we present numerical simulations supporting these predictions and suggesting similar qualitative behaviour beyond the exact 2-design assumption, including for structured ansätze such as the quantum approximate optimization algorithm^46^.

#### Further applications

In the unital-noise setting, the same techniques yield improved barren-plateau upper bounds compared with ref. ^21^, with a tighter dependence on depth and an explicit dependence on Pauli weight (Methods). Moreover, we show that fidelity quantum kernels^47–49^ exhibit exponential concentration at any depth under broad noise models, and we provide corresponding worst-case bounds under mild additional assumptions (Methods).

## Discussion

Our work develops a unified picture of how random quantum circuits behave under local, possibly non-unital noise. First, we establish an effective-depth principle: for the task of estimating observable expectation values, most noisy circuits behave as shallow circuits of logarithmic depth, regardless of their actual depth. Importantly, this conclusion requires essentially no structure of the noise beyond locality and incoherence. As such, it applies to physically relevant noise models, including dephasing, and may have implications for settings where decoherence competes with measurement dynamics, such as measurement-induced phase transitions^50,51^.

Second, we show that, unlike in the noiseless and unital-noise settings, local cost functions do not exhibit barren plateaus under non-unital noise: they neither concentrate exponentially around a fixed value nor develop exponentially small gradients. This observation must be interpreted with care. The absence of barren plateaus is not, by itself, good news for variational algorithms: it arises because only the final $\varTheta (\log n)$ layers are effectively trainable, while parameters deeper in the circuit have a negligible influence—again reflecting effective logarithmic depth.

Third, we leverage this picture to obtain classical simulation algorithms for estimating Pauli expectation values, on average over the circuit ensemble. In particular, for one-dimensional architectures the resulting algorithms remain efficient even for inverse-polynomial precision at arbitrary depth, while for general architectures they are efficient for constant precision.

Taken together, our results suggest a simple message: for expectation-value estimation without access to fresh auxiliary qubits, typical circuits under non-unital noise are not qualitatively more powerful than under depolarizing noise. Indeed, depolarizing noise is known to confine meaningful computation to logarithmic depth^29,30^; while non-unital noise can in principle be exploited to surpass this barrier in specially engineered constructions (for example, the quantum refrigerator^27^), our results indicate that such an advantage is not generic. In this sense, depolarizing and non-unital noise impose qualitatively similar logarithmic-depth limitations for typical circuits in expectation-value estimation. At the same time, our work highlights that overly simplified depolarizing models can be misleading for many platforms, and motivates a more careful characterization of the underlying noise when assessing quantum advantage.

Our results raise several follow-up questions. On the technical side, it is natural to ask whether the trace-distance indistinguishability bound in equation (4) can be established at depths smaller than linear in *n* (for example, logarithmic), and whether the verification/early-break condition in equation (11) is satisfied with high probability over the circuit ensemble. Another direction is to relax the 2-design assumption on the gate distribution, in line with our numerics, and to extend the analysis to more complex noise models, including spatially correlated two-qubit noise (with correlations decaying in distance) and non-Markovian noise (with correlations decaying in time).

**Fig. 1:**
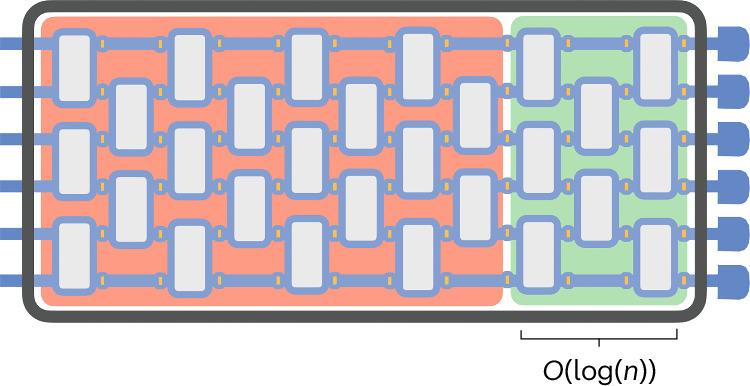
Effective depth of noisy circuits. For most of the quantum circuits with any possibly non-unital noise, only the last $O(\log (n))$ influence significantly observables expectation values. Here, *n* is the number of qubits.

More broadly, while our analysis identifies structural limitations of noisy random circuits for expectation-value estimation and trainability, the complexity of sampling from such circuits remains a subtle open problem. Finally, it would be interesting to explore whether our techniques can shed light on related dynamical phenomena, including measurement-induced phase transitions^50,51^, and to understand how the picture changes once one allows mid-circuit measurements and classical feedforward, where interlayer independence no longer holds (Fig. 1).

## Methods

### Preliminaries and definitions

Because our main statements concern average-case behaviour, we begin by defining the random-circuit ensembles over which expectations are taken. We consider *n*-qubit circuits *Φ* built from layers of two-qubit gates interleaved with local (single-qubit) noise, and optionally followed by a final layer of single-qubit gates. Unless stated otherwise, no geometric locality is assumed. All gate layers are drawn from (local) unitary 2-designs, and all of our bounds use only second-moment information.

We represent the depth-*L* noisy circuit as the channel

20 $$\varPhi :={{\mathcal{V}}}^{{\rm{s}}{\rm{i}}{\rm{n}}{\rm{g}}{\rm{l}}{\rm{e}}}\circ {{\mathcal{N}}}^{\otimes n}\circ {{\mathcal{U}}}_{L}\circ \cdots \circ {{\mathcal{N}}}^{\otimes n}\circ {{\mathcal{U}}}_{1},$$

where ${{\mathcal{U}}}_{\ell }$ is the channel induced by the *ℓ*th two-qubit unitary layer (*ℓ* ∈ [*L*]: = {1, …, *L*}), ${\mathcal{N}}$ is a single-qubit quantum channel modelling local noise, and ${{\mathcal{V}}}^{{\rm{s}}{\rm{i}}{\rm{n}}{\rm{g}}{\rm{l}}{\rm{e}}}(\cdot ):=V(\cdot ){V}^{\dagger }$ with $V{=\bigotimes }_{i=1}^{n}{U}_{i}$ is a final single-qubit layer. This last layer is not essential and can be removed with minor modifications.

**Normal form for single-qubit noise**. Because we average over two-qubit 2-designs and work only up to second moments, we may conjugate ${\mathcal{N}}$ on the left and right by arbitrary unitary channels without changing the relevant distributions. We therefore use the standard ‘normal form’ for a single-qubit channel^31,42^. Writing an input state in Bloch form as *ρ* = (*I* + **w** ⋅ **σ**)/2, with **σ** = (*X*, *Y*, *Z*) and ∥**w**∥_2 _≤ 1, we have

21 $${\mathcal{N}}\,\left(\frac{I+{\bf{w}}\cdot {\boldsymbol{\upsigma }}}{2}\right)=\frac{I}{2}+\frac{1}{2}({\bf{t}}+D{\bf{w}})\cdot {\boldsymbol{\upsigma }},$$

where ${\bf{t}}=({t}_{X},{t}_{Y},{t}_{Z})\in {{\mathbb{R}}}^{3}$ and *D* = diag(**D**) with ${\bf{D}}=({D}_{X},{D}_{Y},{D}_{Z})\in {{\mathbb{R}}}^{3}$. A key quantity throughout is

22 $$c:=\frac{1}{3}\left(\parallel {\bf{D}}{\parallel }_{2}^{2}+\parallel {\bf{t}}{\parallel }_{2}^{2}\right),$$

which will control the contraction rate with circuit depth. In particular, *c* ≤ 1, with equality if and only if ${\mathcal{N}}$ is unitary.

We frequently switch to the Heisenberg picture. The adjoint channel ${{\mathcal{N}}}^{* }$ acts on *Q* ∈ {*X*, *Y*, *Z*} as

23 $${{\mathcal{N}}}^{* }(Q)={t}_{Q}I+{D}_{Q}Q,$$

and satisfies ${{\mathcal{N}}}^{* }(I)=I$. In particular, ${\mathcal{N}}$ is unital (that is, ${\mathcal{N}}(I)=I$) if and only if *t*_*Q*_ = 0 for all *Q* ∈ {*X*, *Y*, *Z*}.

**Truncated circuits**. We will often consider subcircuits obtained by retaining only a contiguous block of layers. For 1 ≤ *a* ≤ *b* ≤ *L*, we write

24 $${\varPhi }_{[a,b]}:={{\mathcal{V}}}_{b}^{{\rm{s}}{\rm{i}}{\rm{n}}{\rm{g}}{\rm{l}}{\rm{e}}}\circ {{\mathcal{N}}}^{\otimes n}\circ {{\mathcal{U}}}_{b}\circ \cdots \circ {{\mathcal{V}}}_{a}^{{\rm{s}}{\rm{i}}{\rm{n}}{\rm{g}}{\rm{l}}{\rm{e}}}\circ {{\mathcal{N}}}^{\otimes n}\circ {{\mathcal{U}}}_{a},$$

where ${{\mathcal{V}}}_{\ell }^{single}$ denotes the (possibly present) single-qubit layer immediately following ${{\mathcal{U}}}_{\ell }$. In particular, *Φ*_[*L*−*m*, *L*]_ denotes the truncated circuit consisting only of the last *m* noisy layers.

**Extensions beyond homogeneous single-qubit noise**. For simplicity, we present the model with the same single-qubit channel ${\mathcal{N}}$ acting on every qubit and layer, but the uniformity assumption is not essential: the arguments extend to spatially varying local noise as long as each site is acted upon by a local non-unitary channel. Moreover, our analysis also accommodates certain classes of correlated two-qubit noise appearing after each two-qubit gate, for instance channels of the form

25 $${\mathcal{E}}={\tilde{\mathcal{U}}}_{2}\circ ({{\mathcal{N}}}_{1}\otimes {{\mathcal{N}}}_{2})\circ {\tilde{\mathcal{U}}}_{1},$$

with arbitrary two-qubit unitaries ${\tilde{\mathcal{U}}}_{1},{\tilde{\mathcal{U}}}_{2}$ and single-qubit channels ${{\mathcal{N}}}_{1},{{\mathcal{N}}}_{2}$. Owing to the left and right invariance built into the random-circuit ensemble, our structural conclusions (for example, logarithmic effective depth and lack of concentration for local observables) remain unchanged; see Supplementary Information for more details.

#### Improved upper bounds for barren plateaus in the unital noise scenario

The technical tools developed in this work also allow us, in the unital-noise scenario, to improve upon the barren-plateau upper bounds presented in ref. ^21^. In particular, we establish the following.

#### Proposition 10

*For the improved upper bound on the partial derivative for unital noise*, *let* $C:={\rm{T}}{\rm{r}}(P\varPhi ({\rho }_{0}))$ *be the cost function, where* *P* *is a Pauli*, *ρ*_0_ *is an arbitrary initial state and* *Φ* *is a random quantum circuit ansatz of depth* *L* *in arbitrary dimension*. *We assume that the noise is unital and not unitary*. *Let* *μ* *denote a parameter of any* 2-*qubit gate* $\rm{e}^{-i{\theta }_{\mu }{H}_{\mu }}$ *in the circuit such that* ∥*H*_*μ*_∥_*∞* _≤ 1. *Then, we have*

26 $${\rm{V}}{\rm{a}}{\rm{r}}[{\partial }_{\mu }C]\le \rm{e}^{-\varOmega (| P| +L)}.$$

It is noteworthy that the upper bound of ref. ^21^ has no dependence on the Pauli weight, unlike ours. Furthermore, the upper bound of ref. ^21^ includes an *n*^1/2^ factor in front of the exponential decay in *L*, making it meaningful only at depths $\varOmega (\log (n))$, whereas our result is without such a factor. Moreover, our result is more general than that shown in ref. ^21^ because it extends to any unital noise, whereas the results shown in ref. ^21^ apply only to primitive Pauli noise, which is only a particular type of unital noise (for example, dephasing is not included in this class).

#### Exponential concentration and lack thereof in noisy kernels

As a complementary result, we demonstrate how quantum kernels^47–49^ exhibit a similar behaviour to cost functions under the influence of non-unital noise. In particular, we show that fidelity quantum kernels^47–49^ can incur an exponential concentration at any depth. Assuming that the noise parameters satisfy $\parallel {\bf{t}}{\parallel }_{2}^{2}+\parallel {\bf{D}}{\parallel }_{2}^{2} < 1$, we show that

27 $${{\mathbb{E}}}_{\varPhi ,{\varPhi }^{{\prime} }}[{\rm{T}}{\rm{r}}[\varPhi (\rho ){\varPhi }^{{\prime} }(\rho )]]\le \rm{e}^{-\varOmega (n)},$$

for any two noisy quantum circuits *Φ* and *Φ*′, where the expectation is taken over the random gates in both the circuits. This result follows by upper bounding the overlap of two states in terms of their purities by the Cauchy–Schwarz inequality, expanding the expected purities in the Pauli basis, and upper bounding the contribution of each Pauli by means of theorem 5.

Furthermore, we are able to show worst-case concentration bounds by introducing an additional assumption on the noise model. In particular, we assume that the noise channel ${\tilde{\mathcal{N}}}$ is the composition of the depolarizing channel ${{\mathcal{N}}}_{p}^{({\rm{d}}{\rm{e}}{\rm{p}})}$ with constant *p* > 0 and an arbitrary noise channel with ∥**t**∥_2_ < 1, that is, ${\tilde{\mathcal{N}}}:={\mathcal{N}}\circ {{\mathcal{N}}}_{p}^{({\rm{d}}{\rm{e}}{\rm{p}})}$. Thus, we obtain

28 $${\rm{T}}{\rm{r}}[\varPhi (\rho ){\varPhi }^{{\prime} }(\rho )]\le {2}^{n({\delta }_{L}-1)},$$

where ${\delta }_{L}:={(1-p)}^{2L}+\parallel {\bf{t}}{\parallel }_{2}\frac{1-{(1-p)}^{2L}}{2p-{p}^{2}}$. We emphasize that, when the noise is purely depolarizing, that is, ∥**t**∥_2_ = 0, this bound predicts exponential concentration at any depth, thus improving a previous result given in ref. ^47^, predicting exponential concentration at linear depth.

## Online content

Any methods, additional references, Nature Portfolio reporting summaries, source data, extended data, supplementary information, acknowledgements, peer review information; details of author contributions and competing interests; and statements of data and code availability are available at 10.1038/s41567-026-03245-z.

## Supplementary information

Supplementary Information Supplementary Information.

## Data Availability

The code used to generate the numerical results and figures in this Article is available via GitHub at https://github.com/AntMele/Noise-induced-shallow-circuits-and-the-absence-of-barren-plateaus.
