# Supplementary material for: Noise-induced shallow circuits and the absence of barren plateaus
Source: Nat Phys. 2026 Apr 2;22(5):751–6. doi: 10.1038/s41567-026-03245-z (PMC13183580; doi:10.1038/s41567-026-03245-z)
Supplement: Supplementary file 1 — Supplementary Information. [file 41567_2026_3245_MOESM1_ESM.pdf]

nature physics

Supplementary information

https://doi.org/10.1038/s41567-026-03245-z

Noise-induced shallow circuits and the absence of barren plateaus

In the format provided by the authors and unedited

Supplementary Material

Contents

I. Preliminaries 2
A. Notation and basic definitions 2
B. Haar measure and unitary designs 3
1. Useful relations 3
2. Properties of layers of single-qubit random gates 5
C. Quantum channels 6
1. Pauli transfer matrix representation of a quantum channel 6
2. Normal form representation of a quantum channel 7
D. Circuit and noise model 9
1. Assumption on the circuit distribution 10
2. Noise model 10

II. Related works 12

III. Observable expectation values of noisy random quantum circuits 13
A. Variance lower bound: Local expectation values with non-unital noise are not exponentially concentrated 14
B. Variance upper bound: Global expectation values are exponentially concentrated 15
C. Effective shallow circuits 17
D. Indistinguishability of quantum states affected by noisy quantum circuits 19
1. Indistinguishability in terms of the trace distance 19
2. Worst-case upper bound on the trace distance 20
E. Classical simulation of Pauli expectation values of noisy random quantum circuits 22
F. Classical simulation via depth- and Pauli-weight-truncated light cones 25

IV. Quantum machine learning under non-unital noise: Barren plateaus 32
A. Preliminaries on barren plateaus 32
1. Cost functions 32
2. Review of previous results 33
B. Gradients: useful lemmas 34
C. Absence of barren plateaus, but only few layers are trainable 37
1. Partial derivative upper bound: Layers before the last $O(\log(n))$ are not trainable 38
2. Partial derivative lower bound: the last $\Theta(\log(n))$ layers are the only trainable 40
D. Improved upper bounds for unital noise 44

V. Purity and kernel methods under non-unital noise 46
A. Purity of average and worst-case circuits 46
1. Purity of an average-case noisy circuit 46
2. Purity of a worst-case noisy circuit 47
B. Quantum machine learning under non-unital noise: Kernel methods 48
1. Preliminaries on quantum kernel methods 48
2. Kernel-based supervised learning 48
3. Assumption on the training data distribution. 49
4. Fidelity quantum kernels: Exponential concentration at any depth 49

VI. Miscellaneous 49
A. Trace distance decay for worst-case circuits under local depolarizing noise 50
B. Numerical simulations 51

References 52

I. Preliminaries

A. Notation and basic definitions

Throughout this work, we employ the following notation and conventions.

- $\mathcal{L}(\mathbb{C}^d)$ denotes the set of linear operators that act on the d-dimensional complex vector space $\mathbb{C}^d$.
- The *unitary group*, denoted as $\mathrm{U}(d)$, comprises operators $U \in \mathcal{L}(\mathbb{C}^d)$ satisfying $U^\dagger U = I$, with I representing the identity operator. Additionally, we use $[d]$ to represent the set of integers from 1 to d, i.e., $[d] := \{1, \dots, d\}$.
- Given a vector $v \in \mathbb{C}^d$ and a value $p \in [1, \infty]$, we denote the p-norm of v as $\left\|v\right\|_p$, defined as

$$\left\|v\right\|_p := \left(\sum_{i=1}^{d} |v_i|^p\right)^{1/p}. \tag{1}$$

- **Norms:** For a matrix $A \in \mathcal{L}\left(\mathbb{C}^d\right)$, its *Schatten p-norm* is $\left\|A\right\|_p := \mathrm{Tr}((\sqrt{A^\dagger A})^p)^{1/p}$, corresponding to the p-norm of the vector of singular values of A.

  The trace norm and Hilbert-Schmidt norm, specific instances of Schatten p-norms, are, respectively, denoted as $\left\|\cdot\right\|_1$ and $\left\|\cdot\right\|_2$.

  The infinity norm, $\left\|\cdot\right\|_\infty$, of a matrix is the maximum singular value, which is equal to the limit of the Schatten p-norm as p approaches infinity.

  The Hilbert-Schmidt norm arises from the scalar product $\langle A, B\rangle_{HS} := \mathrm{Tr}\left(A^\dagger B\right)$ for $A, B \in \mathcal{L}\left(\mathbb{C}^d\right)$.

  The *Hölder inequality*, $|\langle A, B\rangle_{HS}| \le \left\|A\right\|_p \left\|B\right\|_q$, holds for $1 \le p, q \le \infty$ such that $p^{-1} + q^{-1} = 1$.

  For all matrices A and $1 \le p \le q$, we have $\left\|A\right\|_q \le \left\|A\right\|_p$ and $\left\|A\right\|_p \le \mathrm{rank}(A)^{(p^{-1}-q^{-1})}\left\|A\right\|_q$. In particular, we have that $\left\|A\right\|_1 \le \sqrt{d}\left\|A\right\|_2$.
- **Quantum states:** The set of density matrices (*quantum states*) is

$$\mathcal{S}\left(\mathbb{C}^d\right) := \{\rho \in \mathcal{L}\left(\mathbb{C}^d\right) : \rho \ge 0,\ \mathrm{Tr}(\rho) = 1\}. \tag{2}$$

  We adopt the bra-ket notation, denoting a vector $v \in \mathbb{C}^d$ as $|v\rangle$ and its adjoint as $\langle v|$. A vector $|\psi\rangle \in \mathbb{C}^d$ is a (pure) state vector if $\left\||\psi\rangle\right\|_2 = 1$. The canonical basis of $\mathbb{C}^d$ is $\{|i\rangle\}_{i=1}^d$, and the non-normalized maximally entangled state vector is given by $|\Omega\rangle := \sum_{i=1}^d |i\rangle \otimes |i\rangle = \sum_{i=1}^d |i, i\rangle$.
- Given an element of $A \in \mathcal{L}(\mathbb{C}^{d_1} \otimes \mathbb{C}^{d_2})$, we indicate with $\mathrm{Tr}_1(A)$ the partial trace of A with respect the first subsystem, and similarly for $\mathrm{Tr}_2(A)$, for partial trace of A with respect the second subsystem.
- When addressing a system of n qubits, we use I to denote the identity operator on the Hilbert space $\mathbb{C}^2$ of one qubit, while $I_n = I^{\otimes n}$ denotes the identity on the Hilbert space of n qubits.
- **Pauli basis:** Let $d = 2^n$, where $n \in \mathbb{N}$. Elements of the Pauli basis $\{I, X, Y, Z\}^{\otimes n}$ are Hermitian, unitary, trace-less, they square to the identity and they are orthogonal to each other with respect the Hilbert-Schmidt Scalar product. The Pauli basis forms an orthogonal basis for the linear operators $\mathcal{L}\left(\mathbb{C}^d\right)$. We denote $\boldsymbol{\sigma} := (X, Y, Z)$ the vector of single qubit Pauli matrices.

  Given $P \in \{I, X, Y, Z\}^{\otimes n}$ such that $P = Q_1 \otimes \cdots \otimes Q_n$, we define $[P]$ the set $[P] := \{Q_1, \dots, Q_n\}$.
- **Support:** We define the *support of* $P \in \{I, X, Y, Z\}^{\otimes n}$ as the set of integers containing the non-identity terms in $[P]$, i.e.,

$$\mathrm{supp}(P) := \{i \in [n] : Q_i \ne I\}. \tag{3}$$

  For example, $\mathrm{supp}(X \otimes I \otimes Y) = \{1, 3\}$.

  Similarly, if H is an operator expressed in the Pauli basis as $H = \sum_{i=1}^M c_i P_i$, where $\{c_i\}_{i=1}^M$ are real non-zero numbers, and $\{P_i\}_{i=1}^M$ are elements of the Pauli basis, then

$$\mathrm{supp}(H) := \bigcup_{i=1}^{M} \mathrm{supp}(P_i). \tag{4}$$

- **Pauli weight:** The *Pauli-weight* of P, denoted as $|P|$, is the number of Pauli in the tensor product decomposition of P different from I, namely $|P| := |\mathrm{supp}(P)|$. For example $|X \otimes I \otimes Y| = |\{1,3\}| = 2$. The Pauli-$X$ weight of P, denoted as $|P|_X$, corresponds to the number of Pauli-X operators in the tensor-product decomposition of P. Similarly, the Pauli-Y weight of P, denoted as $|P|_Y$, represents the number of Pauli-Y operators in the decomposition of P. Analogously, the Pauli-Z weight is denoted as $|P|_Z$.

- **Locality of an observable:** The *locality* of a Hermitian operator H is defined as $|\mathrm{supp}(H)|$.

- **Light cone:** The light-cone of a Hermitian operator H with respect to a linear map Φ is defined as

$$\mathrm{Light}(\Phi, H) := \mathrm{supp}(\Phi^*(H)), \tag{5}$$

  where Φ^* denotes the adjoint of Φ with respect the Hilbert-Schmidt scalar product.

- **Circuit layer:** A unitary operator is said to form a circuit layer if it can be expressed as a tensor product of two-qubit gates (not necessarily nearest neighbors).

- **Geometrical locality:** A circuit $U = U_L U_{L-1} \dots U_1$ is said to have geometrical locality $\mathrm{D} > 0$ if, for any observable H and unitary layers $U_1, \dots, U_L$, the Heisenberg-evolved observable $U_{j+1}^\dagger U_{j+2}^\dagger \dots U_L^\dagger H U_L \dots U_{j+2} U_{j+1}$ is supported on at most $|\mathrm{supp}(H)|(2m)^{\mathrm{D}}$ qubits, where $m := L - j$. This definition aligns and it is implied by other common definitions of geometric locality. A circuit without any assumptions on geometric locality is said to have all-to-all connectivity, in which case the Heisenberg-evolved observable $U_{j+1}^\dagger U_{j+2}^\dagger \dots U_L^\dagger H U_L \dots U_{j+2} U_{j+1}$ is supported on at most $|\mathrm{supp}(H)| 2^m$ qubits.

- **Asymptotic notation:** Big-O notation: For a function $f(n)$, if there exists a constant c and a specific input size n_0 such that $f(n) \le c \cdot g(n)$ for all $n \ge n_0$, where $g(n)$ is a well-defined function, then we express it as $f(n) = O(g(n))$. This notation signifies the upper limit of how fast a function grows in relation to $g(n)$.

  Big-Omega notation: For a function $f(n)$, if there exists a constant c and a specific input size n_0 such that $f(n) \ge c \cdot g(n)$ for all $n \ge n_0$, where $g(n)$ is a well-defined function, then we express it as $f(n) = \Omega(g(n))$. This notation signifies the lower limit of how fast a function grows in relation to $g(n)$.

  Big-Theta notation: For a function $f(n)$, if $f(n) = O(g(n))$ and if $f(n) = \Omega(g(n))$, where $g(n)$ is a well-defined function, then we express it as $f(n) = \Theta(g(n))$.

  Little-Omega notation: For a function $f(n)$, if for any constant c, there exists an input size n_0 such that $f(n) > c \cdot g(n)$ for all $n \ge n_0$, where $g(n)$ is a well-defined function, then then we express it as $f(n) = \omega(g(n))$. This notation implies that the function grows strictly faster than the provided lower bound.

B. Haar measure and unitary designs

In the following, for our proofs it will be useful to have some familiarity with the Haar measure, which formalizes the notion of uniform distribution over unitaries. For a more detailed explanation we refer to Ref. [1] – here we state a few crucial properties that will be useful later on. We define as the *Haar measure* $\mu_H(\mathrm{U}(d))$ the (unique) probability distribution over the unitary group $\mathrm{U}(d)$ which is left and right invariant, which means that for any integrable function f, we have

$$\mathop{\mathbb{E}}_{U\sim\mu_H}[f(U)] = \mathop{\mathbb{E}}_{U\sim\mu_H}[f(UV)] = \mathop{\mathbb{E}}_{U\sim\mu_H}[f(VU)], \tag{6}$$

for any $U, V \in \mathrm{U}(d)$. In the last equation, we have used $\mu_H \equiv \mu_H(\mathrm{U}(d))$, that is, we omitted to specify the group $\mathrm{U}(d)$, and we will do the same in subsequent sections.Moreover, it holds that

$$\mathop{\mathbb{E}}_{U\sim\mu_H}[f(U)] = \mathop{\mathbb{E}}_{U\sim\mu_H}\left[f(U^\dagger)\right]. \tag{7}$$

1. *Useful relations*

We define the *identity* $\mathbb{I}$ and the *flip operator* $\mathbb{F}$, also known as the permutation operators associated to a tensor product of two Hilbert spaces, as

$$\mathbb{I} := \sum_{i,j=1}^{d} |i,j\rangle\langle i,j|, \qquad \mathbb{F} := \sum_{i,j=1}^{d} |i,j\rangle\langle j,i|. \tag{8}$$

From this definition, it can be observed that they satisfy

$$\mathbb{I}\left(|\psi\rangle \otimes |\phi\rangle\right) = |\psi\rangle \otimes |\phi\rangle, \qquad \mathbb{F}\left(|\psi\rangle \otimes |\phi\rangle\right) = |\phi\rangle \otimes |\psi\rangle, \tag{9}$$

for all $|\psi\rangle, |\phi\rangle \in \mathbb{C}^d$. Useful properties of the flip operator are the *swap-trick* and the *partial-swap-trick*

$$\operatorname{Tr}(A \otimes B\mathbb{F}) = \operatorname{Tr}(AB), \quad \operatorname{Tr}_2(A \otimes B\mathbb{F}) = AB, \tag{10}$$

equalities that can be easily verified in terms of tensor network diagrams.

Let n now be the number of qubits in a system. If $d = 2^n$, then the flip operator can be written in terms of the Pauli basis as

$$\mathbb{F} = \frac{1}{d} \sum_{P \in \{I,X,Y,Z\}^{\otimes n}} P \otimes P, \tag{11}$$

where we have used the fact that the Pauli basis is an orthogonal basis and the *swap-trick*.

Two formulas, involving expected values over the Haar measure and permutation operators, will be crucial in our proofs. Given $O \in \mathcal{L}\left(\mathbb{C}^d\right)$, we have the so called *first-moment formula*, given by

$$\mathop{\mathbb{E}}_{U \sim \mu_H}\left[UOU^\dagger\right] = \frac{\operatorname{Tr}(O)}{d} I. \tag{12}$$

Given $O \in \mathcal{L}((\mathbb{C}^d)^{\otimes 2})$, we have the second-moment formula

$$\mathop{\mathbb{E}}_{U \sim \mu_H}\left[U^{\otimes 2} O U^{\dagger \otimes 2}\right] = c_{\mathbb{I},O}\mathbb{I} + c_{\mathbb{F},O}\mathbb{F}, \tag{13}$$

where

$$c_{\mathbb{I},O} = \frac{\operatorname{Tr}(O) - d^{-1}\operatorname{Tr}(\mathbb{F}O)}{d^2 - 1} \quad \text{and} \quad c_{\mathbb{F},O} = \frac{\operatorname{Tr}(\mathbb{F}O) - d^{-1}\operatorname{Tr}(O)}{d^2 - 1}. \tag{14}$$

(see Ref. [1] for a proof of the previous two equations). A probability distribution over unitaries ν is defined to be a *k-design* [2], for $k \in \mathbb{N}$, if and only if

$$\mathop{\mathbb{E}}_{U \sim \mu_H}\left[U^{\otimes k} O U^{\dagger \otimes k}\right] = \mathop{\mathbb{E}}_{V \sim \nu}\left[V^{\otimes k} O V^{\dagger \otimes k}\right]. \tag{15}$$

If the distribution ν is a $(k+1)$-design, then it is also a k-design. An important set of unitaries which will be useful in our work is the Clifford group [3].

Definition 1 (Clifford group [3, 4])**.** *The Clifford group* $\mathrm{Cl}(n)$ *is the set of unitaries which sends the Pauli group* $\mathcal{P}_n := \{i^k\}_{k=0}^3 \times \{I,X,Y,Z\}^{\otimes n}$ *in itself under the adjoint operation,*

$$\mathrm{Cl}(n) := \{U \in \mathrm{U}(2^n) \colon UPU^\dagger \in \mathcal{P}_n \text{ for all } P \in \{I,X,Y,Z\}^{\otimes n}\}, \tag{16}$$

and it is equivalent to the set of unitaries generated by $\{\mathrm{H}, \mathrm{CNOT}, \mathrm{S}\}$ *where* H, CNOT, *and* S *are, respectively, the* Hadamard, Controlled-NOT*, and* Phase *gates.*

We make extensive use of the following seminal result throughout our work.

Lemma 2 (Clifford group is a 2-design [5, 6])**.** *The uniform distribution over the Clifford group* $\mathrm{Cl}(n)$ *is a 2-design.*

The Clifford group is actually also a 3-design [5, 6], but in our work we need only its 2-design property. Now, we state an important formula – the Pauli mixing formula – that we use in many of the proofs.

Lemma 3 (Pauli mixing)**.** *Let $d = 2^n$ and consider ν to be a 2-design distribution. If $P_1, P_2 \in \{I,X,Y,Z\}^{\otimes n}$ are elements of the Pauli basis, then*

$$\mathop{\mathbb{E}}_{U \sim \nu}\left[U^{\otimes 2}(P_1 \otimes P_2)U^{\dagger \otimes 2}\right] = \delta_{P_1,P_2} \mathop{\mathbb{E}}_{U \sim \nu}\left[U^{\otimes 2}(P_1 \otimes P_1)U^{\dagger \otimes 2}\right] \tag{17}$$

$$= \begin{cases} I \otimes I & \text{if } P_1 = P_2 = I, \\ \frac{1}{d^2-1}\sum_{P \in \{I,X,Y,Z\}^{\otimes n} \setminus I_n} P \otimes P & \text{if } P_1 = P_2 \neq I, \\ 0 & \text{if } P_1 \neq P_2. \end{cases} \tag{18}$$

This can be shown using the second-moment formula previously introduced and the decomposition of the flip operator in terms of the Pauli basis.

2. Properties of layers of single-qubit random gates

In our work, frequent calculations involve averages over the tensor product of single-qubit 2-design gates. To facilitate these calculations, we introduce two Lemmas that will be instrumental in later sections of our work.

Lemma 4 (A layer of 1-qubit Haar random gates is a global 1-design)**.** *Let ν be a distribution over the tensor product of single-qubit 1-design gates, namely over unitaries U of the form $U = \bigotimes_{i=1}^{n} u_i$, where u_i is a single-qubit unitary acting on the i-th qubit. Then, ν is a n-qubit 1-design.*

Proof. Let $O \in \mathcal{L}\left(\mathbb{C}^d\right)$ with $d = 2^n$. By writing it in the Pauli basis, we have

$$\mathop{\mathbb{E}}_{U\sim\nu}\left[UOU^\dagger\right] = \frac{1}{d}\sum_{P\in\{I,X,Y,Z\}^{\otimes n}} \mathrm{Tr}(OP)\mathop{\mathbb{E}}_{U\sim\nu}\left[UPU^\dagger\right] = \frac{\mathrm{Tr}(O)}{d}I_n, \tag{19}$$

where in the last equality we have used the first-moment formula (Eq. (12)) on each of the qubits and used that the Pauli are trace-less. □

As a consequence of the Pauli mixing property, we have the following lemma.

Lemma 5 (Second moments of single-qubit random gates layers)**.** *Let ν be a distribution over the tensor product of single-qubit 2-design gates, namely over unitaries U of the form $U = \bigotimes_{i=1}^{n} u_i$, where u_i is a single-qubit unitary acting on the i-th qubit. Let B be any operator. Then we have*

1. *Let $O \coloneqq \sum_{P\in\{I,X,Y,Z\}^{\otimes n}} a_P P$, with $a_P \in \mathbb{R}$ for any $P \in \{I,X,Y,Z\}^{\otimes n}$. We have*

$$\mathop{\mathbb{E}}_{U\sim\nu}\left[\mathrm{Tr}\left(OUBU^\dagger\right)^2\right] = \sum_{P\in\{I,X,Y,Z\}^{\otimes n}} a_P^2 \mathop{\mathbb{E}}_{U\sim\nu}\left[\mathrm{Tr}\left(PUBU^\dagger\right)^2\right]. \tag{20}$$

2. *For any $P \in \{I,X,Y,Z\}^{\otimes n}$, we have*

$$\mathop{\mathbb{E}}_{U\sim\nu}\left[\mathrm{Tr}\left(PUBU^\dagger\right)^2\right] = \frac{1}{3^{|P|}}\sum_{\substack{Q\in\{I,X,Y,Z\}^{\otimes n}:\\ \mathrm{supp}(Q)=\mathrm{supp}(P)}} \mathrm{Tr}(QB)^2. \tag{21}$$

Proof. We have

$$\begin{aligned}
\mathop{\mathbb{E}}_{U\sim\nu}[\mathrm{Tr}\left(OUBU^\dagger\right)^2] &= \mathop{\mathbb{E}}_{U\sim\nu}[\mathrm{Tr}\left(O^{\otimes 2}U^{\otimes 2}B^{\otimes 2}U^{\dagger\otimes 2}\right)] && (22)\\
&= \sum_{P,Q\in\{I,X,Y,Z\}^{\otimes n}} a_P a_Q \mathop{\mathbb{E}}_{U\sim\nu}[\mathrm{Tr}\left((P\otimes Q)U^{\otimes 2}B^{\otimes 2}U^{\dagger\otimes 2}\right)]\\
&= \sum_{P,Q\in\{I,X,Y,Z\}^{\otimes n}} a_P a_Q \mathop{\mathbb{E}}_{U\sim\nu}[\mathrm{Tr}\left(U^{\dagger\otimes 2}(P\otimes Q)U^{\otimes 2}B^{\otimes 2}\right)]\\
&= \sum_{P\in\{I,X,Y,Z\}^{\otimes n}} a_P^2 \mathop{\mathbb{E}}_{U\sim\nu}[\mathrm{Tr}\left(U^{\dagger\otimes 2}(P\otimes P)U^{\otimes 2}B^{\otimes 2}\right)]\\
&= \sum_{P\in\{I,X,Y,Z\}^{\otimes n}} a_P^2 \mathop{\mathbb{E}}_{U\sim\nu}[\mathrm{Tr}\left(PUBU^\dagger\right)^2],
\end{aligned}$$

where for the fourth equality we have used the fact that $U = \bigotimes_{i=1}^{n} u_i$ is a layer of single-qubit 2-design unitaries,

$$\mathop{\mathbb{E}}_{U\sim\mu_H}[f(U)] = \mathop{\mathbb{E}}_{U\sim\mu_H}\left[f(U^\dagger)\right] \tag{23}$$

for any measurable function f, and the Pauli mixing property in Eq. (17) for each of the single-qubit unitaries to conclude that $\mathop{\mathbb{E}}_{u_i\sim\mu_H}[u_i^{\otimes 2}(P_1\otimes P_2)u_i^{\dagger\otimes 2}] = 0$ for two different single-qubit Pauli P_1 and P_2.

Similarly, for $P \in \{I,X,Y,Z\}^{\otimes n}$ such that $P = P_1 \otimes P_2 \otimes \cdots \otimes P_n$, we use the Pauli-mixing property in Eq. (18), along with the fact that $U = \bigotimes_{i=1}^{n} u_i$ is a tensor product of single-qubit unitaries from a 2-design, to obtain

$$\mathop{\mathbb{E}}_{U\sim\nu}[\mathrm{Tr}\left(PUBU^\dagger\right)^2] = \mathop{\mathbb{E}}_{U\sim\nu}[\mathrm{Tr}\left(U^{\dagger\otimes 2}(P\otimes P)U^{\otimes 2}B^{\otimes 2}\right)] \tag{24}$$

$$= \mathrm{Tr}\left(\mathop{\mathbb{E}}_{U\sim\nu} \left[\bigotimes_{i=1}^{n} u_i^{\otimes 2} (P_i \otimes P_i) u_i^{\dagger\otimes 2} \right] B^{\otimes 2} \right)$$
$$= \mathrm{Tr}\left(\mathop{\mathbb{E}}_{U\sim\nu} \left[\bigotimes_{i\in \mathrm{supp}(P)} u_i^{\otimes 2} (P_i \otimes P_i) u_i^{\dagger\otimes 2} \right] B^{\otimes 2} \right)$$
$$= \mathrm{Tr}\left(\left(\bigotimes_{i\in \mathrm{supp}(P)} \frac{1}{3} \sum_{Q_i \in \{X,Y,Z\}} Q_i \otimes Q_i \right) B^{\otimes 2} \right)$$
$$= \frac{1}{3^{|P|}} \sum_{\substack{Q\in\{I,X,Y,Z\}^{\otimes n}: \\ \mathrm{supp}(Q)=\mathrm{supp}(P)}} \mathrm{Tr}\big(Q^{\otimes 2} B^{\otimes 2}\big),$$

which is the desired result, because $\mathrm{Tr}\big(Q^{\otimes 2} B^{\otimes 2}\big) = \mathrm{Tr}(QB)^2$. □

C. Quantum channels

A *quantum channel* $\mathcal{N} : \mathcal{L}(\mathbb{C}^d) \to \mathcal{L}(\mathbb{C}^d)$ is a linear, completely positive, and trace-preserving map. Completely positive means that for all positive operators $\sigma \in \mathcal{L}(\mathbb{C}^d \otimes \mathbb{C}^D)$, for any $D \in \mathbb{N}$, the operator $(\mathcal{N} \otimes \mathcal{I})(\sigma)$ is positive. The trace-preserving property means that $\mathrm{Tr}(\mathcal{N}(A)) = \mathrm{Tr}(A)$ for any $A \in \mathcal{L}\left(\mathbb{C}^d\right)$. Here, $\mathcal{I} : \mathcal{L}\left(\mathbb{C}^D\right) \to \mathcal{L}\left(\mathbb{C}^D\right)$ denotes the identity map. Any quantum channel $\mathcal{N}$ can be represented in terms of at most d^2 Kraus operators $\{K_i\}_{i=1}^{d^2}$, i.e.,

$$\mathcal{N}(\cdot) = \sum_{i=1}^{d^2} K_i \,(\cdot)\, K_i^\dagger, \tag{25}$$

with the condition $\sum_{i=1}^{d^2} K_i^\dagger K_i = I$ to satisfy trace-preservation. Given a quantum channel $\mathcal{N}$, we say that $\mathcal{N}$ is *unital* if and only if it maps the identity operator to the identity operator, i.e., $\mathcal{N}(I) = I$. Otherwise, we say that $\mathcal{N}$ is *non-unital*. Given a quantum channel $\mathcal{N} : \mathcal{L}(\mathbb{C}^d) \to \mathcal{L}(\mathbb{C}^d)$, its adjoint map $\mathcal{N}^* : \mathcal{L}(\mathbb{C}^d) \to \mathcal{L}(\mathbb{C}^d)$ is defined as the linear map such that

$$\langle \mathcal{N}^*(A), B\rangle_{HS} = \langle A, \mathcal{N}(B)\rangle_{HS} \tag{26}$$

for any $A, B \in \mathcal{L}\left(\mathbb{C}^d\right)$. If $\{K_i\}_{i=1}^{d^2}$ is a set of Kraus operators for $\mathcal{N}$, then the adjoint channel $\mathcal{N}^*$ can be expressed as

$$\mathcal{N}^*(\cdot) = \sum_{i=1}^{d^2} K_i^\dagger (\cdot) K_i. \tag{27}$$

Note that $\mathcal{N}^*$ is always unital, $\mathcal{N}^*(I) = I$, inherited from the property of the channel being trace-preserving. However the adjoint is not necessarily trace-preserving: it holds that $\mathcal{N}^*$ is trace preserving if and only if $\mathcal{N}$ is unital. If the Kraus operators of the quantum channel $\mathcal{N}$ are Hermitian, then the adjoint channel coincides with the quantum channel $\mathcal{N}^* = \mathcal{N}$. If $\mathcal{N}_1, \mathcal{N}_2$ are two quantum channels, then $(\mathcal{N}_1 \circ \mathcal{N}_2)^* = \mathcal{N}_2^* \circ \mathcal{N}_1^*$. Moreover $(\mathcal{N}_1 \otimes \mathcal{N}_2)^* = \mathcal{N}_1^* \otimes \mathcal{N}_2^*$. For any Hermitian operator O, we have [7]

$$\|\mathcal{N}^*(O)\|_\infty \leq \|O\|_\infty. \tag{28}$$

1. *Pauli transfer matrix representation of a quantum channel*

In this subsection, we introduce the Pauli transfer matrix representation of a single-qubit quantum channel. Let $\mathcal{N} : \mathcal{L}(\mathbb{C}^2) \to \mathcal{L}(\mathbb{C}^2)$ be a linear map. Any linear map can be expressed in terms of its action on the Pauli basis, i.e.

$$\mathcal{N}(P) = \sum_{Q\in\{I,X,Y,Z\}} T_{Q,P} Q, \tag{29}$$

where $T_{Q,P} := \frac{1}{2}\mathrm{Tr}(Q\mathcal{N}(P))$. Assuming that $\mathcal{N}$ represents a quantum channel, it inherently preserves Hermiticity, implying that $T_{Q,P} \in \mathbb{R}$. Furthermore, by employing the *Hölder inequality*, we establish that $|T_{Q,P}| \leq 1$ for all $P, Q \in \{I, X, Y, Z\}$.

Given that a quantum channel is trace-preserving and the Pauli matrices are all trace-less except for the identity, we deduce that $T_{I,P} = \delta_{I,P}$. Consequently, we have

$$\mathcal{N}(I) = I + T_{X,I}X + T_{Y,I}Y + T_{Z,I}Z, \tag{30}$$
$$\mathcal{N}(X) = T_{X,X}X + T_{Y,X}Y + T_{Z,X}Z, \tag{31}$$
$$\mathcal{N}(Y) = T_{X,Y}X + T_{Y,Y}Y + T_{Z,Y}Z, \tag{32}$$
$$\mathcal{N}(Z) = T_{X,Z}X + T_{Y,Z}Y + T_{Z,Z}Z. \tag{33}$$

From Eqs. (30)-(33), we can see that considering a non-unital noise channel is equivalent to assuming that at least one of the parameters $T_{X,I}$, $T_{Y,I}$, or $T_{Z,I}$ must be non-zero.

We define the Pauli transfer matrix $\mathrm{T}(\mathcal{N})$ of the channel $\mathcal{N}$ as the matrix with components defined as $[\mathrm{T}(\mathcal{N})]_{Q,P} := \frac{1}{2}\operatorname{Tr}(Q\mathcal{N}(P)) = T_{Q,P}$ for all $Q, P \in \{I, X, Y, Z\}$, i.e.,

$$\mathrm{T}(\mathcal{N}) = \begin{bmatrix} 1 & 0 & 0 & 0 \\ T_{X,I} & T_{X,X} & T_{X,Y} & T_{X,Z} \\ T_{Y,I} & T_{Y,X} & T_{Y,Y} & T_{Y,Z} \\ T_{Z,I} & T_{Z,X} & T_{Z,Y} & T_{Z,Z} \end{bmatrix}. \tag{34}$$

It is important to note that any single-qubit quantum channel can be expressed in such a form. However, not every linear map of this form represents a valid quantum channel. Furthermore, utilizing the definition of the adjoint map, we can easily verify that the adjoint map $\mathcal{N}^*$ is given by $\mathcal{N}^*(P) = \sum_{Q\in\{I,X,Y,Z\}} T_{P,Q}Q$. This results in the fact that the Pauli transfer matrix of the adjoint channel is the transpose of the Pauli transfer matrix of the channel, i.e.,

$$\mathrm{T}(\mathcal{N}^*) = \mathrm{T}(\mathcal{N})^T. \tag{35}$$

Given two quantum channels $\mathcal{N}^{(A)}$ and $\mathcal{N}^{(B)}$, we have that the Pauli transfer matrix associated to their composition is given by the multiplication of their Pauli transfer matrices:

$$\mathrm{T}(\mathcal{N}^{(A)} \circ \mathcal{N}^{(B)}) = \mathrm{T}(\mathcal{N}^{(A)}) \cdot \mathrm{T}(\mathcal{N}^{(B)}). \tag{36}$$

2. *Normal form representation of a quantum channel*

We now present a quantum channel representation [8, 9] that will be useful when dealing with noisy random circuits. In words, it says that the Pauli transfer matrix of a single-qubit noise channel, up to unitary rotations, is diagonal in the sub-block corresponding to the non-identity Pauli matrices. We include here the lemma and proof of this representation for easy reference.

Lemma 6 (Normal form of a quantum channel [8, 9])**.** *Any single-qubit quantum channel $\mathcal{N}$ can be written in the so called 'normal' form:*

$$\mathcal{N}(\cdot) = U\mathcal{N}'(V^\dagger(\cdot)V)U^\dagger, \tag{37}$$

where U, V are unitaries and $\mathcal{N}'(\cdot)$ is a quantum channel with Pauli transfer matrix

$$\mathrm{T}(\mathcal{N}') = \begin{bmatrix} 1 & 0 & 0 & 0 \\ t_X & D_X & 0 & 0 \\ t_Y & 0 & D_Y & 0 \\ t_Z & 0 & 0 & D_Z \end{bmatrix}, \tag{38}$$

where $\mathbf{t} := (t_X, t_Y, t_Z)$ and $\mathbf{D} := (D_X, D_Y, D_Z) \in \mathbb{R}^3$, such that the entries of $\mathbf{D}$ have all the same sign.

Proof. Let us consider the Pauli transfer matrix of $\mathcal{N}$, which is characterized by the real 3×3 matrix $B \in \mathcal{L}(\mathbb{R}^3)$ and the vector $\boldsymbol{b} = (b_X, b_Y, b_Z) \in \mathbb{R}^3$:

$$\mathrm{T}(\mathcal{N}) = \begin{bmatrix} 1 & 0 & 0 & 0 \\ b_X & B_{X,X} & B_{X,Y} & B_{X,Z} \\ b_Y & B_{Y,X} & B_{Y,Y} & B_{Y,Z} \\ b_Z & B_{Z,X} & B_{Z,Y} & B_{Z,Z} \end{bmatrix}. \tag{39}$$

Any single qubit quantum state ρ can be written as $\rho = (I + \mathbf{w}\cdot\boldsymbol{\sigma})/2$, where $\mathbf{w} := (w_X, w_Y, w_Z) \in \mathbb{R}^3$ with $\|w\|_2 \le 1$ and $\boldsymbol{\sigma} := (X, Y, Z)$. Then, we have

$$\mathcal{N}(\rho) = \mathcal{N}\left(\frac{I}{2}\right) + \frac{1}{2}\mathcal{N}(\mathbf{w}\cdot\boldsymbol{\sigma}) = \left(\frac{I}{2} + \frac{1}{2}\boldsymbol{b}\cdot\boldsymbol{\sigma}\right) + \frac{1}{2}(B\mathbf{w})\cdot\boldsymbol{\sigma} = \frac{I}{2} + \frac{1}{2}(\boldsymbol{b} + B\mathbf{w})\cdot\boldsymbol{\sigma}. \tag{40}$$

Next, because B is real, we can perform a real singular value decomposition of B and have $B = R_1 D R_2^T$, where D is a diagonal matrix with the non-negative diagonal elements $(D_X, D_Y, D_Z) \in \mathbb{R}^3$ and R_1, R_2 are in general orthogonal O(3) matrices. Now, every orthogonal matrix has determinant equal to ± 1. This fact, along with the fact that $\det(-R) = (-1)^3\det(R) = -\det(R)$ for every $R \in \mathrm{O}(3)$, means that we can, without loss of generality, assume that R_1 and R_2 both have determinant equal to 1. In other words, we can assume that R_1 and R_2 are both special-orthogonal matrices in $\mathrm{SO}(3)$. The diagonal elements (D_X, D_Y, D_Z) are then not necessarily non-negative, but they all have the same sign. We now use the fact that for every special-orthogonal matrix $R \in \mathrm{SO}(3)$, there exists a unitary $U \in \mathrm{U}(2)$ such that [10]

$$(R\boldsymbol{v})\cdot\boldsymbol{\sigma} = U(\boldsymbol{v}\cdot\boldsymbol{\sigma})U^\dagger, \tag{41}$$

for all $\boldsymbol{v} \in \mathbb{R}^3$. The previous identity can be easily verified by choosing $U := \exp\left(-i\frac{\theta}{2}\hat{n}\cdot\boldsymbol{\sigma}\right)$, where $\hat{n}$ and θ are, respectively, the unit-norm vector and the rotation angle which characterizes the special-orthogonal matrix $R \in \mathrm{SO}(3)$. Thus, we have that

$$\mathcal{N}(\rho) = U\left(\frac{I}{2} + \frac{1}{2}(R_1^T\boldsymbol{b} + DR_2^T\mathbf{w})\cdot\boldsymbol{\sigma}\right)U^\dagger = U\mathcal{N}'\left(\frac{I + (R_2^T\mathbf{w})\cdot\boldsymbol{\sigma}}{2}\right)U^\dagger = U\mathcal{N}'\left(V^\dagger\left(\frac{I + \mathbf{w}\cdot\boldsymbol{\sigma}}{2}\right)V\right)U^\dagger, \tag{42}$$

where U and V are the unitaries associated to the special-orthogonal matrices R_1 and R_2, and $\mathcal{N}'$ is the linear map such that

$$\mathcal{N}'(\frac{I + \mathbf{w}\cdot\boldsymbol{\sigma}}{2}) = \frac{I + (\mathbf{t} + D\mathbf{w})\cdot\boldsymbol{\sigma}}{2}, \tag{43}$$

where $\mathbf{t} := R_1^T\boldsymbol{b}$. Hence, we have shown that $\mathcal{N}(\rho)$ can be written as $\mathcal{N}(\rho) = U\mathcal{N}'\left(V^\dagger\rho V\right)U^\dagger$, where the Pauli transfer matrix of $\mathcal{N}'$ is the one in Eq. (38). □

Thus, every single-qubit quantum channel $\mathcal{N}$ can be expressed as $\mathcal{N}(\cdot) = U\mathcal{N}'(V^\dagger(\cdot)V)U^\dagger$, where U, V are unitaries, and $\mathcal{N}'$ is a quantum channel such that it acts on a quantum state written in its Bloch sphere representation as

$$\mathcal{N}'\left(\frac{I + \mathbf{w}\cdot\boldsymbol{\sigma}}{2}\right) = \frac{I}{2} + \frac{1}{2}(\mathbf{t} + D\mathbf{w})\cdot\boldsymbol{\sigma}, \tag{44}$$

where $\mathbf{w} \in \mathbb{R}^3$ with $\|\mathbf{w}\|_2 \le 1$, $\mathbf{t} := (t_X, t_Y, t_Z) \in \mathbb{R}^3$ and $D := \mathrm{diag}(\mathbf{D})$ with $\mathbf{D} := (D_X, D_Y, D_Z) \in \mathbb{R}^3$.

We now prove that the parameters of the normal form representation satisfy a particular constrain. Such constrain will be crucial in our following discussion.

Lemma 7 (Contraction coefficient in terms of the normal form parameters)**.** *For any single-qubit quantum channel, the parameters* $\mathbf{t}, \mathbf{D} \in \mathbb{R}^3$ *of its normal form representation satisfy:*

$$c := \frac{1}{3}(t_X^2 + D_X^2 + t_Y^2 + D_Y^2 + t_Z^2 + D_Z^2) \le 1, \tag{45}$$

and the equality is saturated if and only if the channel is unitary. Furthermore, it also holds $\|\mathbf{t}\|_2 \le 1$ *and* $D_P \le 1$ *for all* $P \in \{X, Y, Z\}$.

Proof. Because of the previous Lemma, any single-qubit quantum channel $\mathcal{N}$ can be expressed as $\mathcal{N}(\cdot) = U\mathcal{N}'(V^\dagger(\cdot)V)U^\dagger$, where U, V are unitaries, and $\mathcal{N}'$ such that it holds Eq. (44). Let ρ be an arbitrary qubit quantum state. Noting that $\mathcal{N}'$ is a quantum channel, on account of being a composition of quantum channels, it holds that $\|\mathcal{N}'(\rho)\|_\infty \le 1$. If we let $\mathbf{w} \in \mathbb{R}^3$ be the Bloch vector corresponding to ρ, then because $\|\mathcal{N}'(\rho)\|_\infty$ is equal to the largest eigenvalue of $\mathcal{N}'(\rho)$, we find that

$$1 \ge \|\mathcal{N}'(\rho)\|_\infty = \frac{1}{2}(1 + \|\mathbf{t} + D\mathbf{w}\|_2). \tag{46}$$

Hence, we get

$$(t_X + D_X w_x)^2 + (t_Y + D_Y w_y)^2 + (t_Z + D_Z w_z)^2 = \|\mathbf{t} + D\mathbf{w}\|_2^2 \le 1. \tag{47}$$

Now, recall that $\|\mathbf{w}\|_2 \leq 1$. If $\mathbf{w} = 0$, then we get $\|\mathbf{t}\|_2 \leq 1$. In particular by choosing $w = (\pm 1, 0, 0)$, we get

$$(t_X \pm D_X)^2 \leq 1, \tag{48}$$

and similarly for Y and Z (from which follows that $D_P \leq 1$ for all $P \in \{X, Y, Z\}$). Now, assume that the entries of $\mathbf{D}$ are all non-negative (remember that they have the same sign). Together with the previous equation, this implies that

$$t_X^2 + D_X^2 + t_Y^2 + D_Y^2 + t_Z^2 + D_Z^2 \leq (t_X + \text{sign}(t_X)D_X)^2 + (t_Y + \text{sign}(t_Y)D_Y)^2 + (t_Z + \text{sign}(t_Z)D_Z)^2 \leq 3. \tag{49}$$

Similarly, if the entries of $\mathbf{D}$ are all negative, we have

$$t_X^2 + D_X^2 + t_Y^2 + D_Y^2 + t_Z^2 + D_Z^2 \leq (t_X - \text{sign}(t_X)D_X)^2 + (t_Y - \text{sign}(t_Y)D_Y)^2 + (t_Z - \text{sign}(t_Z)D_Z)^2 \leq 3. \tag{50}$$

This proves Eq. (45).

Finally, we show that Eq. (45) is saturated if and only if $\mathcal{N}$ is unitary. If $\mathcal{N}$ is unitary, then also $\mathcal{N}'$ is unitary. This implies that $\mathbf{t} = 0$, because unitary channels are also unital. Moreover, it also implies that the purity of any state must remain the same, so the diagonal matrix $D := \text{diag}(\mathbf{D})$ must be norm-preserving, hence orthogonal. Therefore, we have $D = \pm\text{diag}(1,1,1)$. This saturates inequality (45). Now, let us assume that

$$\frac{1}{3}(t_X^2 + D_X^2 + t_Y^2 + D_Y^2 + t_Z^2 + D_Z^2) = 1. \tag{51}$$

From the inequality (48), we get also that $t_X^2 + D_X^2 \leq 1$, and the same for Y and Z. Hence, Eq. (51) implies $t_X^2 + D_X^2 = 1$, and the same for Y and Z. Using this with Eq. (48), we have that the possible values for t_X^2 and D_X^2 are, respectively, 1 and 0, or vice versa. Similarly for Y and Z. From Eq. (47), we deduce that the only possibility is that $\mathbf{t} = 0$ and that $D_X^2 = D_Y^2 = D_Z^2 = 1$. Hence, we have that the Pauli transfer matrix of $\mathcal{N}'$ is equal, up to a possible minus sign factor, to the identity matrix. This implies that $\mathcal{N}'$ must be the identity channel and that $\mathcal{N}$ is unitary. □

Here, we give examples of normal form parameters $\mathbf{t} = (t_X, t_Y, t_Z)$ and $\mathbf{D} = (D_X, D_Y, D_Z)$ for standard noise channels. The single-qubit depolarizing channel with parameter $p \in [0,1]$ can be defined as

$$\mathcal{N}_p^{(\text{dep})}(\sigma) := (1-p)\sigma + p\,\text{Tr}(\sigma)\frac{I}{2}. \tag{52}$$

Its normal form parameters are $\mathbf{t} = (0,0,0)$ and $\mathbf{D} = (1-p, 1-p, 1-p)$. The amplitude damping channel $\mathcal{N}_q^{(\text{amp})}$, parameterized by $q \in [0,1]$, is given in the computational basis as

$$\mathcal{N}_q^{(\text{amp})}(\sigma) = \begin{pmatrix} \sigma_{0,0} + q\sigma_{1,1} & \sqrt{1-q}\sigma_{0,1} \\ \sqrt{1-q}\sigma_{1,0} & (1-q)\sigma_{1,1} \end{pmatrix}, \tag{53}$$

where $\sigma_{i,j} := \langle i|\,\sigma\,|j\rangle$. Here, $\mathbf{t} = (0,0,q)$ and $\mathbf{D} = (\sqrt{1-q}, \sqrt{1-q}, 1-q)$. The single-qubit dephasing channel $\mathcal{N}_p^{(\text{deph})}$ with $p \in [0,1]$ can be defined as

$$\mathcal{N}_p^{(\text{deph})}(\sigma) = \begin{pmatrix} \sigma_{0,0} & (1-p)\sigma_{0,1} \\ (1-p)\sigma_{1,0} & \sigma_{1,1} \end{pmatrix}. \tag{54}$$

Its normal form parameters are $\mathbf{t} = (0,0,0)$ and $\mathbf{D} = (1-p, 1-p, 1)$.

D. Circuit and noise model

In our work, we examine n-qubit quantum circuits Φ formed by layers of two-qubit random unitary gates interleaved by local noise, with a final layer of random single qubit gates. For example, the standard brickwork circuit architecture is within our model (Figure 1). Mathematically,

$$\Phi := \mathcal{V}^{\text{single}} \circ \mathcal{N}^{\otimes n} \circ \mathcal{U}_L \circ \cdots \circ \mathcal{N}^{\otimes n} \circ \mathcal{U}_1, \tag{55}$$

where $\mathcal{V}^{\text{single}} := V(\cdot)V^\dagger$ with $V := \bigotimes_{i=1}^n u_i$ is a layer of single-qubit gates, $\mathcal{U}_i := U_i(\cdot)U_i^\dagger$ corresponds to the n-qubit unitary channel associated with the i-th unitary layer U_i for $i \in [L]$, and $\mathcal{N}$ is a single-qubit quantum channel. We point out that the single-qubit layer $\mathcal{V}^{\text{single}}$ included in our model is not essential for our main results and can be omitted with minor modifications.

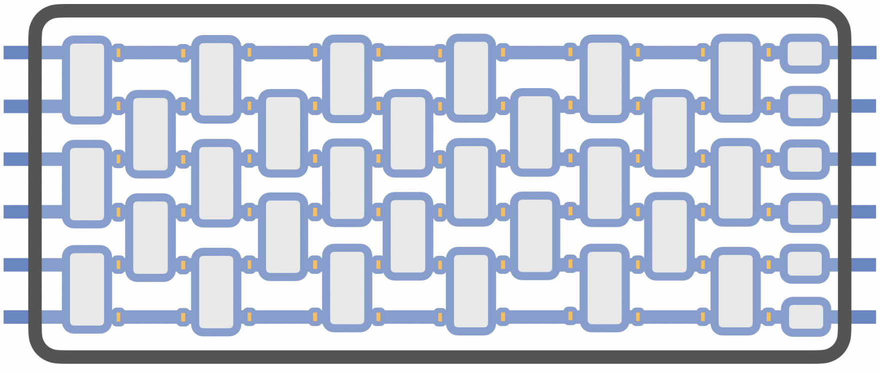

Figure 1. Example of the architecture that our model encompasses: A brickwork circuit composed of two-qubit gates followed by local noise (depicted with yellow circles).

1. Assumption on the circuit distribution

Firstly, we assume that each single-qubit gate in the layer $\bigotimes_{i=1}^{n} u_i$ is distributed according to a single-qubit 2-design (e.g., Haar random). Moreover, we assume that each unitary layer $\mathcal{U}_i := U_i(\cdot)U_i^\dagger$, for $i \in [L]$, consists of two-local qubit gates, each forming a local 2-design. More precisely, we assume that each U_i for $i \in [L]$ is distributed according to a 2-local 2-design layer distribution, defined as follows:

Definition 8 (2-local 2-design layer distribution)**.** *We say that ν is a 2-local 2-design layer distribution if and only if it is a probability distribution over quantum circuits formed by local 2-qubit gates, where each of them is distributed accordingly to a local 2-design and each qubit is acted on by at least one of the gates.*

Moreover, we point out that we consider an arbitrary circuit geometry/architecture, i.e., we do not make any particular assumptions on the geometric dimensionality of our circuit, except when explicitly mentioned. Note that our model is in stark contrast to works [11, 12] where the unitary layers are chosen as global n-qubit 2-designs, and we expect that the model that we consider is more realistic.

2. Noise model

Moreover, since before and after any noise channel $\mathcal{N}$ there is a gate that is distributed according to a 2-design and in our work we consider up to second moment quantities, because of the normal form representation of the channel and unitary invariance, we can restrict the noise channels $\mathcal{N}$ to have a sparse Pauli transfer matrix of the form of Eq. (38), characterized by two real vectors $\mathbf{t} := (t_X, t_Y, t_Z)$ and $\mathbf{D} := (D_X, D_Y, D_Z)$. In particular, the adjoint channel $\mathcal{N}^*$ acts on $Q \in \{X, Y, Z\}$ as

$$\mathcal{N}^*(Q) = t_Q I + D_Q Q = \sum_{a \in \{0,1\}} D_Q^a t_Q^{1-a} Q^a. \tag{56}$$

Since we work with at most second-moment quantities, we often single out 'for free' from each 2-local 2-design unitary layer $\{\mathcal{U}_i\}_{i=1}^{L}$ layers of single qubit Haar random gates, due to the invariance of the Haar measure and because each qubit is a acted on by at least one of the 2-local 2-design gates. Specifically, without loss of generality, we can consider equivalently circuits of the form

$$\Phi = (\mathcal{V}_L^{\text{single}} \circ \mathcal{N}^{\otimes n} \circ \mathcal{U}_L) \circ \cdots \circ (\mathcal{V}_1^{\text{single}} \circ \mathcal{N}^{\otimes n} \circ \mathcal{U}_1), \tag{57}$$

where $\{\mathcal{V}_k^{\text{single}}\}_{k=1}^{L}$ are layers of single-qubit gates distributed according a single-qubit 2-design. However, these single-qubit layers do not play a fundamental role in our model and could be omitted. (In particular, the last single-qubit layer might also be omitted with minor modifications: if the circuit ends with a layer of noise rather than a single-qubit layer, this noise layer and the preceding unitary layer can be absorbed into the observable in the Heisenberg picture. Thus, we can reduce the scenario to one where the circuit terminates with a layer of single-qubit gates, due to unitary invariance, and with an observable of comparable locality.)

We will often denote circuits derived from Φ by removing the last layer of single-qubit gates and the last layer of noise. In this case, we use the notation

$$\Phi' := (\mathcal{N}^{\otimes n} \circ \mathcal{U}_L) \circ \cdots \circ (\mathcal{V}_1^{\text{single}} \circ \mathcal{N}^{\otimes n} \circ \mathcal{U}_1), \tag{58}$$

$$\Phi'' := \mathcal{U}_L \circ \cdots \circ (\mathcal{V}_1^{\mathrm{single}} \circ \mathcal{N}^{\otimes n} \circ \mathcal{U}_1). \tag{59}$$

Here, Φ' denotes the circuit without the final layer of single-qubit gates, while Φ'' denotes the circuit without the final layer of single-qubit gates layer and also without the final layer of noise.

We also often need to denote circuits derived from Φ by retaining certain layers from the start or from the end. In these cases, we use subscripts to indicate the relevant layers. That is, for $a \leq b \in [L]$,

$$\Phi_{[a,b]} \coloneqq (\mathcal{V}_b^{\mathrm{single}} \circ \mathcal{N}^{\otimes n} \circ \mathcal{U}_b) \circ \cdots \circ (\mathcal{V}_a^{\mathrm{single}} \circ \mathcal{N}^{\otimes n} \circ \mathcal{U}_a). \tag{60}$$

Whenever we write an expectation value, $\mathbb{E}[\cdot]$ or $\mathrm{Var}[\cdot]$, without explicitly specifying the underlying distribution, we consider the probability distribution over the defined random circuit.

II. Related works

Effective depth and input-state independence. Several works have established that non-unital noise can erase dependence on the input state, yielding a notion of *effective depth* [12, 13]. These results, however, address regimes that are markedly different from ours. Ref. [13] proves that, for *worst-case* circuits interleaved with non-unital noise, depths exceeding $\exp(\Omega(n))$ are sufficient for the output state to become effectively independent of the input. Here we show that input-state independence already emerges at *linear* depth, albeit only *on average* over a well-motivated random-circuit ensemble. A comparable worst-case statement at linear depth is impossible in general, since non-unital noise can be exploited to perform fault-tolerant quantum computation up to $\exp(O(n))$ depth without fresh auxiliary qubits (the "quantum refrigerator" construction) [14].

Ref. [12] shows input-state independence for circuits composed of $\Omega(n)$ layers of *global* 2-designs interspersed with non-unital noise. In contrast, our model assumes only that *local* two-qubit gates form a 2-design, which is arguably closer to realistic circuit architectures.

Complementary contraction bounds under families of non-unital noise have also been derived for specific algorithmic settings, including quantum annealers and limitations for the *quantum approximate optimization algorithm* (QAOA) [15, 16]. Our results provide a different perspective: for the task of estimating observable expectation values, *typical* deep circuits under general local non-unitary noise behave like *shallow* circuits. This connects to a broader literature on the power and limitations of shallow quantum circuits. For a wide range of combinatorial optimization problems, shallow quantum circuits can be outperformed by classical algorithms [15–20], while shallow quantum circuits are nevertheless provably stronger than shallow classical circuits in other settings [21–23].

Barren plateaus, random circuits, and noise. The interplay between barren plateaus, random circuits, and noise has been extensively studied in the context of *variational quantum algorithms* (VQAs) [24]. In the noiseless setting, it was shown that if the parameter distribution forms a global 2-design then generic cost functions exhibit barren plateaus [25–27]. The role of locality of observables has been explored in Refs. [28–30]. A number of proposals aim to mitigate barren plateaus in noiseless settings, via initialization heuristics [26, 31–37] or by restricting expressibility [38–48]. It has also been conjectured that provable barren-plateau avoidance often coincides with efficient classical simulation (possibly after an initial data-acquisition stage) [49].

In noisy settings, Ref. [50] introduced *noise-induced barren plateaus* (NIBPs), showing that for certain unital noise models cost functions and gradients decay exponentially with depth, independently of initialization. More recently, Ref. [11] showed that barren plateaus may also occur under non-unital noise for circuits built from *global* 2-design layers. Our setting differs: we consider local-gate architectures with noise acting after each local gate, and in this model we prove that non-unital noise leads to qualitatively different behavior than unital noise. Concretely, we show that local cost functions avoid exponential concentration at any depth, and that only the last $O(\log n)$ layers remain trainable, implying an absence of barren plateaus despite arbitrarily large depth. By contrast, under depolarizing noise sufficiently deep circuits become untrainable [50]. Related observations that engineered Markovian noise can help with trainability were reported in Ref. [51], albeit for a single noisy layer.

More broadly, non-unital noise is known to change qualitative conclusions in several contexts. For example, fault-tolerant computation without fresh ancillas is possible up to $\exp(O(n))$ depth under certain non-unital noise models [14], whereas under depolarizing noise the corresponding depth is only $O(\log n)$ [52, 53] (and under dephasing only $O(\mathrm{poly}(n))$). In random-circuit sampling, Ref. [54] pointed out that existing easiness/hardness arguments can break down under non-unital noise because the output distribution may fail to anticoncentrate [55]. In many-body settings, a growing literature studies entanglement and phase-structure in noisy circuits under different noise models [56–58]. An independent study released contemporaneously with ours analyzes barren plateaus beyond unital noise and reaches conclusions consistent with ours, though under different assumptions (e.g., Hilbert–Schmidt contractivity) [59].

Classical simulation. Classical simulation of expectation values has a long history; for shallow circuits and local observables, light-cone arguments yield efficient computation [60, 61]. More recent works study simulation under structured noise, such as Pauli noise [62, 63], and related tensor-network perspectives appear in Refs. [64, 65]. In the depolarizing-noise regime, sufficiently deep circuits become close to maximally mixed, so estimating Pauli expectations reduces to outputting 0 [50, 53]. Our work shows that this simplification fails under non-unital noise: local expectation values remain non-trivially fluctuating, yet *typical* circuits become effectively shallow for expectation-value estimation, enabling an average-case classical simulation algorithm based on truncation and light-cone methods. For approximate *sampling* under depolarizing noise, an efficient classical algorithm was given in Ref. [66]; extending such analyses to non-unital noise remains challenging and is currently open, consistent with the issues highlighted in Ref. [54]. After our work appeared, Ref. [67] established polynomial-time simulation guarantees for certain noise models (including depolarizing noise and a constrained amplitude-damping family) with success probability over random input states, whereas our simulation results apply to arbitrary local noise channels and hold on average over the circuit ensemble.

III. Observable expectation values of noisy random quantum circuits

In this section, we analyze expectation values of random quantum circuits under possibly non-unital noise. We make here a summary of our results that we analyze in detail in their respective subsections. We consider a circuit architecture Φ as we described in Subsection I D, where the local noise channels are characterized by the parameters of their normal form representation $\mathbf{t} := (t_X, t_Y, t_Z)$ and $\mathbf{D} := (D_X, D_Y, D_Z)$, which we assume to be constants with respect to the number of qubits. Our first main theorem is the following.

Theorem 9 (Variance of expectation values of random circuits with non-unital noise)**.** *Let* $H := \sum_{P\in\{I,X,Y,Z\}^{\otimes n}} a_P P$*, with* $a_P \in \mathbb{R}$ *for* $P \in \{I,X,Y,Z\}^{\otimes n}$*, be an arbitrary Hamiltonian. Let* ρ *be a quantum state. We assume that the noise is non-unital, specifically* $\|\mathbf{t}\|_2 = \Theta(1)$*. Then, at any depth of the noisy circuit* Φ*, as defined in Eq. (55), we have*

$$\mathrm{Var}[\mathrm{Tr}(H\Phi(\rho))] = \sum_{P\in\{I,X,Y,Z\}^{\otimes n}\setminus I^{\otimes n}} a_P^2 \exp(-\Theta(|P|)). \tag{61}$$

To prove Theorem 9, we make use of results we show in Subsection III A and Subsection III B, where we respectively show a lower bound on the variance (Proposition 12) and a matching upper bound (Proposition 14).

Theorem 9 directly implies that the variance of expectation value of local observables (i.e., *local expectation values*) can be significantly large, e.g., $\mathrm{Var}[\mathrm{Tr}(Z_1\Phi(\rho))] = \Omega(1)$. This means that local expectation values can deviate significantly from their mean value $\mathbb{E}[\mathrm{Tr}(H\Phi(\rho))]$. This is in stark contrast to the behaviour of noiseless random quantum circuits or circuits with unital noise [30, 50]. Theorem 9 also implies that the variance of expectation value of global observables (i.e., *global expectation values*) are exponentially concentrated to their mean value, e.g., $\mathrm{Var}[\mathrm{Tr}(Z^{\otimes n}\Phi(\rho))] = \exp(-\Omega(n))$.

Moreover, based on the results shown in Subsection III C and Subsection III D, we establish the following theorem.

Theorem 10 (Average distance between two quantum states)**.** *Let* $P \in \{I,X,Y,Z\}^{\otimes n}$*. Let* ρ *and* σ *be any quantum states. Consider* Φ *as any noisy random quantum circuit of depth* L*, as defined in Eq. (55). Assume that the noise is not a unitary channel. Then, the following holds:*

$$\mathbb{E}_\Phi[|\,\mathrm{Tr}(P\Phi(\rho)) - \mathrm{Tr}(P\Phi(\sigma))|] \le \exp(-\Omega(L+|P|))\,, \tag{62}$$

and for all observables O*, we have*

$$\mathbb{E}_\Phi[|\,\mathrm{Tr}(O\Phi(\rho)) - \mathrm{Tr}(O\Phi(\sigma))|] \le \|O\|_\infty \exp(-\Omega(L))\,. \tag{63}$$

This further implies that for $L = \Omega(n)$*, we have*

$$\mathbb{E}_\Phi[\|\Phi(\rho) - \Phi(\sigma)\|_1] \le \exp(-\Omega(n)), \tag{64}$$

where the expected value is taken over the 2*-design distribution of the two-qubits gates that compose the circuit* Φ*.*

The average trace distance upper bound is proven in Subsection III D, where we prove also a worst-case trace distance upper bound (i.e., without the expected value) that holds in certain high-noise regime. Note that we cannot hope to prove a worst-case upper bound on the trace distance that is valid for every noise regime. This is because there are quantum error correction methods, such as the so-called *quantum refrigerator* construction [14], which can leverage non-unital noise to perform fault-tolerant quantum computation in a model similar to ours, up to depths that are exponential in the number of qubits. Thus, for these special classes of circuits, the trace distance remains of constant order. Moreover, it is known that this result is tight [13], as bounds on the worst-case convergence are known in the regime where the depth is exponential in the number of qubits.

From a direct application of Eq. (63), it follows that with high probability over the choice of the random circuit, considering only the last $O(\log(\varepsilon^{-1}))$ layers suffices for the estimation of expectation values with ε precision. In particular, we get the following statement.

Proposition 11 (Effective depth)**.** *Let* $\varepsilon, \delta > 0$*. Let* O *be any observable, and let* ρ_0 *be any initial state. Consider a noisy quantum circuit* Φ *of depth* L*, as defined in Eq. (55). With probability at least* $1-\delta$ *over the choice of the random circuit, we have*

$$|\,\mathrm{Tr}(O\Phi(\rho_0)) - \mathrm{Tr}\big(O\Phi_{[L-\ell,L]}(\sigma_0)\big)| \le \varepsilon, \tag{65}$$

where σ_0 *is any preferred initial state (e.g.,* $\sigma_0 := |0^n\rangle\langle 0^n|$*). Here,* $\Phi_{[L-\ell,L]}$ *denotes the channel* Φ *restricted to the last* ℓ *layers, where* $\ell := O(\log(\|O\|_\infty/(\delta\varepsilon^2)))$*.*

Note that if O is local and the desired accuracy ε is constant in the number of qubits, then $\mathrm{Tr}\big(O\Phi_{[L-\ell,L]}(\rho_0)\big)$ can be computed efficiently classically via light-cone arguments. Moreover, if $\Phi^*_{[L-\ell,L]}(O)$ is close to something proportional to the identity (which can be verified efficiently classically), then we can certify that our algorithm has succeeded. If O is a global Pauli observable, then we can just output zero for estimating the expectation value with inverse-polynomial precision (because of Eq. (62)). Collectively, these insights underpin a classical simulation algorithm capable of estimating Pauli expectation values of random quantum circuits affected by—possibly non-unital—noise, as explained in Subsection III E. Its runtime depends polynomially on the inverse of the precision for one-dimensional architectures and quasipolynomially for higher-dimensional ones.

A. Variance lower bound: Local expectation values with non-unital noise are not exponentially concentrated

In this subsection, we show that local expectation values of average quantum circuits with non-unital noise can be far from zero, in contrast to what happens with unital noise or in the noiseless case. Let us consider a circuit Φ as described in Subsection I D, where the local noise channel is characterized by the parameters of its normal form representation $\mathbf{t} := (t_X, t_Y, t_Z)$ and $\mathbf{D} := (D_X, D_Y, D_Z)$, and we consider any circuit depth $L \geq 1$.

Proposition 12 (Lower bound on the variance). *Let $H := \sum_{P\in\{I,X,Y,Z\}^{\otimes n}} a_P P$, with $a_P \in \mathbb{R}$ for all $P \in \{I, X, Y, Z\}^{\otimes n}$, be an arbitrary Hamiltonian. Let ρ be a quantum state. Then, for any depth of the noisy circuit Φ, we have*

$$\mathrm{Var}[\mathrm{Tr}(H\Phi(\rho))] \geq \sum_{P\in\{I,X,Y,Z\}^{\otimes n}\setminus I^{\otimes n}} a_P^2 \left(\frac{\|\mathbf{t}\|_2^2}{3}\right)^{|P|}, \tag{66}$$

where we note that $\|\mathbf{t}\|_2$ is non-zero if the noise channel is non-unital.

Proof. Because our circuit ends with a layer of random single qubit gates $\otimes_{i=1}^n u_i$, it holds that $\mathbb{E}[\mathrm{Tr}(P\Phi(\rho))] = 0$ for any $P \in \{I, X, Y, Z\}^{\otimes n} \setminus I^{\otimes n}$, which follows from Lemma 4. We, therefore, have that

$$\mathbb{E}[\mathrm{Tr}(H\Phi(\rho))] = a_{I^{\otimes n}}. \tag{67}$$

We now focus on $\mathbb{E}[\mathrm{Tr}(H\Phi(\rho))^2]$. First of all, using point 1 of Lemma 5, we have

$$\mathbb{E}[\mathrm{Tr}(H\Phi(\rho))^2] = \sum_{P\in\{I,X,Y,Z\}^{\otimes n}} a_P^2 \mathbb{E}[\mathrm{Tr}(P\Phi(\rho))^2] = a_{I^{\otimes n}}^2 + \sum_{P\in\{I,X,Y,Z\}^{\otimes n}\setminus I^{\otimes n}} a_P^2 \mathbb{E}[\mathrm{Tr}(P\Phi(\rho))^2]. \tag{68}$$

Let us now analyze each term $\mathbb{E}[\mathrm{Tr}(P\Phi(\rho))^2]$ in the sum above separately. Using point 2 of Lemma 5, we obtain

$$\mathbb{E}[\mathrm{Tr}(P\Phi(\rho))^2] = \frac{1}{3^{|P|}} \sum_{\substack{Q\in\{I,X,Y,Z\}^{\otimes n}:\\ \mathrm{supp}(Q)=\mathrm{supp}(P)}} \mathbb{E}[\mathrm{Tr}(Q\Phi'(\rho))^2], \tag{69}$$

which corresponds to 'removing' the last layer of single-qubit gates and using the Pauli mixing property. Recall that Φ' denotes the noisy circuit channel without the last layer of single qubit gates, while Φ'' denotes the noisy circuit channel without the last layer of single qubit gates and last layer of noise, i.e., $\Phi' = \mathcal{N}^{\otimes n} \circ \Phi''$. Taking the adjoint of the noise, and using the fact that $\mathcal{N}^*$ is a unital channel, we obtain

$$\begin{aligned}
\mathbb{E}[\mathrm{Tr}(Q\Phi'(\rho))^2] &= \mathbb{E}\left[\mathrm{Tr}\big(\mathcal{N}^{*\otimes n}(Q)\Phi''(\rho)\big)^2\right] \\
&= \mathbb{E}\left[\mathrm{Tr}\left(\left(\bigotimes_{j\in\mathrm{supp}(Q)} \mathcal{N}^*(Q_j)\right)\Phi''(\rho)\right)^2\right] \\
&= \mathbb{E}\left[\mathrm{Tr}\left(\left(\bigotimes_{j\in\mathrm{supp}(Q)} (t_{Q_j} I_j + D_{Q_j} Q_j)\right)\Phi''(\rho)\right)^2\right] \\
&= \mathbb{E}\left[\mathrm{Tr}\left(\left(\sum_{a\in\{0,1\}^{|Q|}} \bigotimes_{j\in\mathrm{supp}(Q)} (t_{Q_j}^{a_j} D_{Q_j}^{1-a_j} Q_j^{1-a_j})\right)\Phi''(\rho)\right)^2\right]
\end{aligned}$$

$$= \sum_{a\in\{0,1\}^{|Q|}} \prod_{j\in\text{supp}(Q)} (t_{Q_j}^{a_j} D_{Q_j}^{1-a_j})^2 \mathbb{E}\left[\text{Tr}\left(\left(\bigotimes_{k\in\text{supp}(Q)} Q_k^{1-a_k}\right)\Phi''(\rho)\right)^2\right] \tag{70}$$

where in the third step we have used the normal-form parametrization of the channel, specifically, Eq. (56). The fifth step follows by observing that we can apply point 1 of Lemma 5, which we can do because Φ'' ends with a 2-local 2-design unitary layer, hence we can single-out from it a layer of single qubit Haar random gates, due to the invariance of the Haar measure and because each qubit is a acted on by at least one of the 2-qubit 2-design gate.

Now, we are left with a sum of positive terms and from such sum we can keep only the term corresponding to identity term, and we lower bound the remaining terms with zero. This implies that

$$\begin{aligned}
&\sum_{a\in\{0,1\}^{|Q|}} \prod_{j\in\text{supp}(Q)} t_{Q_j}^{2a_j} D_{Q_j}^{2(1-a_j)} \mathbb{E}\left[\text{Tr}\left(\left(\bigotimes_{k\in\text{supp}(Q)} Q_k^{1-a_k}\right)\Phi''(\rho)\right)^2\right] \\
&\geq |t_X|^{2|Q|_X} |t_Y|^{2|Q|_Y} |t_Z|^{2|Q|_Z} \mathbb{E}\left[\text{Tr}(I_n\Phi''(\rho))^2\right] \\
&= |t_X|^{2|Q|_X} |t_Y|^{2|Q|_Y} |t_Z|^{2|Q|_Z},
\end{aligned} \tag{71}$$

where, in the last step, we have used simply that density matrices have unit trace. Substituting, we get

$$\mathbb{E}\left[\text{Tr}(P\Phi(\rho))^2\right] \geq \frac{1}{3^{|P|}} \sum_{\substack{Q\in\{I,X,Y,Z\}^{\otimes n}: \\ \text{supp}(Q)=\text{supp}(P)}} |t_X|^{2|Q|_X} |t_Y|^{2|Q|_Y} |t_Z|^{2|Q|_Z} = \frac{1}{3^{|P|}} \left(|t_X|^2 + |t_Y|^2 + |t_Z|^2\right)^{|P|} \tag{72}$$

where we have exploited the multinomial theorem in the last equality. By putting everything together and using the definition of variance, we can conclude. □

Note that if the noise is unital, i.e., $\|\mathbf{t}\|_2 = 0$, the previous lower bound becomes vacuous. As an immediate corollary of the previous inequality we have the following.

Corollary 13 (Local expectation values are not exponentially concentrated on average)**.** *Let $P \in \{I, X, Y, Z\}^{\otimes n}$ be a Pauli operator with weight $|P| = \Theta(1)$. Let us assume that the noise is non-unital, specifically that $\|\mathbf{t}\|_2 = \Theta(1)$. Then, we have*

$$\text{Var}[\text{Tr}(P\Phi(\rho))] = \Theta(1). \tag{73}$$

We can easily translate the fact that the variance is large into the fact that the probability of deviating from the mean is large. For example, let $C := \text{Tr}(P\Phi(\rho))$, with $\sup(|C|) \leq 1$. By using the following probability inequality (see Lemma 63 in the last *miscellaneous* section), we find that

$$\text{Prob}\left(|C - \mathbb{E}[C]| > \sqrt{\frac{\text{Var}[C]}{2}}\right) \geq \frac{1}{8}\text{Var}[C]. \tag{74}$$

Note that for an inverse polynomially small non-unital noise rate, i.e., $\|\mathbf{t}\|_2 = \Omega\left(\frac{1}{\text{poly}(n)}\right)$, we would get that

$$\text{Var}[\text{Tr}(P\Phi(\rho))] = \Omega\left(\frac{1}{\text{poly}(n)}\right), \tag{75}$$

which implies a lack of exponential concentration of local expectation values even for such small non-unital noise regime.

From a more technical perspective, we have shown that *random quantum circuits* with non-unital noise have local expectation values that are not exponentially concentrated. This is in stark contrast with the behavior of random quantum circuits in the noiseless regime or with local depolarizing noise [30], as summarized in Table I.

B. Variance upper bound: Global expectation values are exponentially concentrated

In this section, we show that expectation values of global observables are typically exponentially concentrated around their mean value. As we have previously done, we consider a circuit model as described in Subsection I D. We let

$$c := \frac{1}{3}\left(\|\mathbf{t}\|_2^2 + \|\mathbf{D}\|_2^2\right), \tag{76}$$

Table I. **Concentration of local expectation values for $\Omega(n)$-depth circuits**

**Noise model**	$\mathrm{Var}[\mathrm{Tr}(Z_1\rho)]$
Noiseless [25, 30]	$\exp(-\Theta(n))$
Unital noise [50]	$\exp(-\Theta(n))$
Non-unital noise [this work]	$\Theta(1)$

Table I illustrates that if a state is prepared by a non-unital noisy-random quantum circuit, the expectation value of local observables never exhibits exponential concentration at any depth around a fixed value. This stands in stark contrast to the noiseless and unital noise regimes.

where we recall that $\mathbf{t} := (t_X, t_Y, t_Z)$ and $\mathbf{D} := (D_X, D_Y, D_Z)$ are the local noise channel parameters of its normal form representation. We consider any circuit depth $L \geq 1$. From Lemma 6, we have that $c < 1$ if and only if the channel is not unitary.

Proposition 14 (Variance upper bound)**.** *Let $H := \sum_{P\in\{I,X,Y,Z\}^{\otimes n}} a_P P$, with $a_P \in \mathbb{R}$ for any $P \in \{I,X,Y,Z\}^{\otimes n}$ be an arbitrary Hamiltonian. Let ρ be any quantum state. Then, at any depth of the noisy circuit Φ, as defined in Eq. (55), we have*

$$\mathrm{Var}[\mathrm{Tr}(H\Phi(\rho))] \leq \sum_{P\in\{I,X,Y,Z\}^{\otimes n}\setminus I^{\otimes n}} a_P^2 c^{|P|}, \tag{77}$$

where the parameter c is defined in Eq. (76).

Proof. The proof follows the same initial steps of the proof of Proposition 12. In particular, the mean is $\mathbb{E}[\mathrm{Tr}(H\Phi(\rho))] = a_{I^{\otimes n}}$, and we have

$$\mathbb{E}[\mathrm{Tr}(H\Phi(\rho))^2] = a_{I^{\otimes n}}^2 + \sum_{P\in\{I,X,Y,Z\}^{\otimes n}\setminus I^{\otimes n}} a_P^2 \mathbb{E}[\mathrm{Tr}(P\Phi(\rho))^2], \tag{78}$$

with

$$\mathbb{E}[\mathrm{Tr}(P\Phi(\rho))^2] = \frac{1}{3^{|P|}} \sum_{\substack{Q\in\{I,X,Y,Z\}^{\otimes n}:\\ \mathrm{supp}(Q)=\mathrm{supp}(P)}} \mathbb{E}[\mathrm{Tr}\big(Q^{\otimes 2}\Phi'(\rho)^{\otimes 2}\big)], \tag{79}$$

Therefore, by taking the adjoint of the last layer of noise, as done in the proof of Proposition 12 (specifically, Eq. (70)), we find

$$\mathbb{E}\left[\mathrm{Tr}(Q\Phi'(\rho))^2\right] = \sum_{a\in\{0,1\}^{|Q|}} \prod_{j\in\mathrm{supp}(Q)} (t_{Q_j}^{a_j} D_{Q_j}^{1-a_j})^2 \mathbb{E}\left[\mathrm{Tr}\left(\left(\bigotimes_{j\in\mathrm{supp}(Q)} Q_j^{1-a_j}\right)\Phi''(\rho)\right)^2\right]. \tag{80}$$

Now, using the fact that $\mathrm{Tr}(P\sigma) \leq 1$ for every Pauli P and state σ, we have

$$\begin{aligned}\sum_{a\in\{0,1\}^{|Q|}} \prod_{j\in\mathrm{supp}(Q)} (t_{Q_j}^{a_j} D_{Q_j}^{1-a_j})^2 \mathbb{E}\left[\mathrm{Tr}\left(\left(\bigotimes_{j\in\mathrm{supp}(Q)} Q_j^{1-a_j}\right)\Phi''(\rho)\right)^2\right] &\leq \sum_{a\in\{0,1\}^{|Q|}} \prod_{j\in\mathrm{supp}(Q)} t_{Q_j}^{2a_j} D_{Q_j}^{2(1-a_j)} \\ &= \prod_{j\in\mathrm{supp}(Q)} (t_{Q_j}^2 + D_{Q_j}^2).\end{aligned} \tag{81}$$

Thus, by substituting, we find

$$\begin{aligned}\mathbb{E}[\mathrm{Tr}(P\Phi(\rho))^2] &\leq \frac{1}{3^{|P|}} \sum_{\substack{Q\in\{I,X,Y,Z\}^{\otimes n}:\\ \mathrm{supp}(Q)=\mathrm{supp}(P)}} \prod_{j\in\mathrm{supp}(Q)} (t_{Q_j}^2 + D_{Q_j}^2) \\ &= \frac{1}{3^{|P|}}(t_X^2 + D_X^2 + t_Y^2 + D_Y^2 + t_Z^2 + D_Z^2)^{|P|} \\ &= c^{|P|},\end{aligned} \tag{82}$$

where we have used the multinomial theorem. □

As an immediate corollary of the previous inequality, we find the following corollary.

Corollary 15 (Global expectation values are exponentially concentrated on average)**.** *Let $P \in \{I, X, Y, Z\}^{\otimes n}$ be a Pauli operator with weight $|P| = \Theta(n)$. Then, at any depth of the noisy circuit Φ defined in Eq. (55), and for any constant noise parameters, we have*

$$\mathrm{Var}[\mathrm{Tr}(P\Phi(\rho))] = \exp(-\Theta(n)). \tag{83}$$

By combining the lower bound on the variance derived in the previous section (Proposition 12) with the matching upper bound derived in this section (Proposition 14), we get a proof of Theorem 9.

C. Effective shallow circuits

In the previous Subsection III A, we have shown that local expectation values can have a large variance in the presence of non-unital noise. In this subsection, we identify even more compelling consequences of this feature. We show that such large variance can only be due to the last few layers of the circuit. Specifically, we prove that the layers preceding the last $\Theta(\log(n))$ do not significantly affect observable expectation values. Let us start with proving the following proposition valid for Pauli expectation values.

Proposition 16 (Decay in Pauli expectation values)**.** *Let $P \in \{I, X, Y, Z\}^{\otimes n}$, let ρ and σ be quantum states, and let L be depth of the noisy circuit Φ defined in Eq. (55). Then, we have*

$$\mathbb{E}[\mathrm{Tr}(P\Phi(\rho - \sigma))^2] \leq 4c^{|P|+L-1}, \tag{84}$$

where the parameter c is defined in Eq. (76).

Proof. By removing the last layer of single qubits gates and using Lemma 5, we have

$$\mathbb{E}[\mathrm{Tr}(P\Phi(\rho - \sigma))^2] = \frac{1}{3^{|P|}} \sum_{\substack{Q\in\{I,X,Y,Z\}^{\otimes n}:\\ \mathrm{supp}(Q)=\mathrm{supp}(P)}} \mathbb{E}[\mathrm{Tr}(Q\Phi'(\rho - \sigma))^2]. \tag{85}$$

Now using the exactly the same argument used in Eq. (70) in the proof of Proposition 12, we obtain

$$\mathbb{E}\left[\mathrm{Tr}(Q\Phi'(\rho-\sigma))^2\right] = \sum_{a\in\{0,1\}^{|Q|}} \prod_{j\in\mathrm{supp}(Q)} (t_{Q_j}^{a_j} D_{Q_j}^{1-a_j})^2 \mathbb{E}\left[\mathrm{Tr}\left(\left(\bigotimes_{j\in\mathrm{supp}(Q)} Q_j^{1-a_j}\right)\Phi''(\rho-\sigma)\right)^2\right]. \tag{86}$$

Note that the expected value on the right-hand side can be bounded from above by $\max_{Q\in\{I,X,Y,Z\}^{\otimes n}} \mathbb{E}[\mathrm{Tr}(Q\Phi''(\rho - \sigma))^2]$. Thus, we have

$$\begin{aligned}
\mathbb{E}[\mathrm{Tr}(P\Phi(\rho - \sigma))^2] &\leq \frac{1}{3^{|P|}} \sum_{\substack{Q\in\{I,X,Y,Z\}^{\otimes n}:\\ \mathrm{supp}(Q)=\mathrm{supp}(P)}} \sum_{a\in\{0,1\}^{|Q|}} \prod_{j\in\mathrm{supp}(Q)} (t_{Q_j}^{a_j} D_{Q_j}^{1-a_j})^2 \max_{Q\in\{I,X,Y,Z\}^{\otimes n}} \mathbb{E}[\mathrm{Tr}(Q\Phi''(\rho - \sigma))^2] \\
&= \frac{1}{3^{|P|}} \sum_{\substack{Q\in\{I,X,Y,Z\}^{\otimes n}:\\ \mathrm{supp}(Q)=\mathrm{supp}(P)}} \prod_{j\in\mathrm{supp}(Q)} (t_{Q_j}^2 + D_{Q_j}^2) \max_{Q\in\{I,X,Y,Z\}^{\otimes n}} \mathbb{E}[\mathrm{Tr}(Q\Phi''(\rho - \sigma))^2] \\
&= \frac{1}{3^{|P|}} (\|\mathbf{D}\|_2^2 + \|\mathbf{t}\|_2^2)^{|P|} \max_{Q\in\{I,X,Y,Z\}^{\otimes n}} \mathbb{E}[\mathrm{Tr}(Q\Phi''(\rho - \sigma))^2] \\
&= c^{|P|} \max_{Q\in\{I,X,Y,Z\}^{\otimes n}} \mathbb{E}[\mathrm{Tr}(Q\Phi''(\rho - \sigma))^2],
\end{aligned} \tag{87}$$

where we have used once again the multinomial theorem. Moreover, we can assume that the maximum over the Pauli operators is not achieved by the identity, otherwise the right-hand side of the above inequality would be zero, because Φ'' is trace preserving and $\rho - \sigma$ is traceless. Thus, we have

$$\mathbb{E}[\mathrm{Tr}(P\Phi(\rho - \sigma))^2] \leq c^{|P|} \max_{Q\in\{I,X,Y,Z\}^{\otimes n}\setminus I_n} \mathbb{E}[\mathrm{Tr}(Q\Phi''(\rho - \sigma))^2]. \tag{88}$$

We can assume now that all the two-qubit gates in the circuit are Clifford (see Definition 1), as we are computing a second moment and the Cliffords form a 2-design (Lemma 2). Thus, the two qubit gates of the circuit will also map Paulis to Paulis. Moreover, we assume without loss of generality that before each layer of noise there is a layer of single-qubit 2-design unitaries, as we are computing a second moment over 2-design quantities and we can use the invariance of the Haar measure to do so. Therefore, the Pauli $Q \neq I_n$ above will be mapped by the two-qubits Clifford to another Pauli still different from the identity. Since now we have a circuit that ends with a layer of single qubits 2-design unitaries, which are preceded by a noise layer and a layer of two-qubits 2-design gates, we are in the same situation we faced at the beginning of the proof. So reiterating the argument to the next layer, we have

$$\begin{aligned}\mathbb{E}[\mathrm{Tr}(Q\Phi''(\rho-\sigma))^2] &\leq \max_{R\in\{I,X,Y,Z\}^{\otimes n}\setminus I_n} c^{|R|}\mathbb{E}\left[\mathrm{Tr}\left(R\Phi''_{[1,L-1]}(\rho-\sigma)\right)^2\right] \\ &\leq c \max_{R\in\{I,X,Y,Z\}^{\otimes n}\setminus I_n} \mathbb{E}\left[\mathrm{Tr}\left(R\Phi''_{[1,L-1]}(\rho-\sigma)\right)^2\right],\end{aligned} \tag{89}$$

where we have used the notation $\Phi''_{[1,k]} := \mathcal{U}_k \circ \cdots \circ \mathcal{N}^{\otimes n} \circ \mathcal{U}_1$ and used the fact that the Pauli weight of R is at least one.

Recursively applying the above reasoning to all of the remaining layers of the circuit, and using the fact that for any Pauli operator P we have $|\mathrm{Tr}(P(\rho-\sigma))| \leq \|\rho-\sigma\|_1$ (because of the Hölder inequality), we obtain

$$\mathbb{E}[\mathrm{Tr}(Q\Phi''(\rho-\sigma))^2] \leq c^{L-1}\|\rho-\sigma\|_1^2 \leq 4c^{L-1}, \tag{90}$$

where in the last step we have used using triangle inequality and the fact that quantum states have one-norm equal to one. Substituting back in Eq. (88), we conclude the proof. □

Proposition 16 implies that if ρ and σ are states created by the 'first' part of the same circuit architecture with different parameters of the gates, then if we implement on them a noisy quantum circuit of depth $L = \omega(\log(n))$, on average we have that the influence on the expectation value of the different gates in the first part of the circuit will be super-polynomially small. We now show that the previous bounds for Pauli expectation values imply a bound for any observable O.

Proposition 17 (Bounds applicable for general observables)**.** *Let O be an observable, ρ and σ be quantum states, and let L be the depth of the noisy circuit Φ. Then, we have*

$$\mathbb{E}[|\mathrm{Tr}(O\Phi(\rho)) - \mathrm{Tr}(O\Phi(\sigma))|^2] \leq 4\left(\frac{\|O\|_2^2}{2^n}\right)c^L \leq 4\|O\|_\infty^2 c^L, \tag{91}$$

where the parameter c is defined as in Eq. (76).

Proof. Let $O = \sum_{P\in\{I,X,Y,Z\}^{\otimes n}} c_P P$ be the Pauli decomposition of O. Then, we find

$$\begin{aligned}\mathbb{E}[\mathrm{Tr}(O\Phi(\rho-\sigma))^2] &= \mathbb{E}\left[\left(\sum_{P\in\{I,X,Y,Z\}^{\otimes n}} c_P \mathrm{Tr}(P\Phi(\rho-\sigma))\right)^2\right] \\ &= \sum_{P\in\{I,X,Y,Z\}^{\otimes n}} |c_P|^2 \mathbb{E}[\mathrm{Tr}(P\Phi(\rho-\sigma))^2],\end{aligned} \tag{92}$$

where in the second step we have used the fact that the cross-terms vanish due to Lemma 5. Using the bound from Proposition 16, we have

$$\begin{aligned}\mathbb{E}[\mathrm{Tr}(O\Phi(\rho-\sigma))^2] &\leq \sum_{P\in\{I,X,Y,Z\}^{\otimes n}} |c_P|^2 \cdot 4c^L \\ &= \frac{\|O\|_2^2}{2^n} 4c^L,\end{aligned} \tag{93}$$

where we have used the fact that $\sum_{P\in\{I,X,Y,Z\}^{\otimes n}} |c_P|^2 = \frac{\|O\|_2^2}{2^n}$. Finally, using the inequality $\|O\|_2 \leq \sqrt{2^n}\|O\|_\infty$, we get

$$\mathbb{E}[\mathrm{Tr}(O\Phi(\rho-\sigma))^2] \leq 4\|O\|_\infty^2 c^L. \tag{94}$$

□

Corollary 18. *Let O be an observable, and let ρ and σ be quantum states. Consider a noisy circuit Φ with depth L. Then, the bound*

$$\mathbb{E}_\Phi\left[|\mathrm{Tr}(O\Phi(\rho)) - \mathrm{Tr}(O\Phi(\sigma))|\right] \le 2\left(\frac{\|O\|_2}{\sqrt{2^n}}\right)c^{L/2} \le 2\|O\|_\infty c^{L/2} \tag{95}$$

holds, where the parameter c is defined as in Eq. (76).

This corollary immediately proves Theorem 1 of the main text through a straightforward relabeling of terms.

N otably, from the proof of the previous proposition, it is evident that the assumption that the circuit must terminate with a layer of single-qubit random gates is not essential. The circuit could instead conclude with a layer of noise, as this noise can be effectively absorbed into the observable within the Heisenberg picture.

D. Indistinguishability of quantum states affected by noisy quantum circuits

We now translate the results in the previous section in terms of the trace distance.

1. Indistinguishability in terms of the trace distance

Proposition 19 (Average distance between two quantum states)**.** *Let Φ be a noisy random quantum circuit with of depth L, as defined in Eq. (55). Then, the average trace distance between $\Phi(\rho)$ and $\Phi(\sigma)$, where ρ and σ are two arbitrary quantum states, decays exponentially in L as*

$$\mathbb{E}[\|\Phi(\rho) - \Phi(\sigma)\|_1] \le 2^{n+1}c^{\frac{L-1}{2}}, \tag{96}$$

where we recall the definition of the parameter c in Eq. (76). Thus, for any $\varepsilon > 0$, assuming that $L \ge \frac{1}{\log(c^{-1})}\Omega\big(n + \log\big(\frac{1}{\varepsilon}\big)\big)$, we have that $\mathbb{E}[\|\Phi(\rho) - \Phi(\sigma)\|_1] \le \varepsilon$.

Proof. We have

$$\begin{aligned}(\mathbb{E}[\|\Phi(\rho) - \Phi(\sigma)\|_1])^2 &\le \mathbb{E}[\|\Phi(\rho) - \Phi(\sigma)\|_1^2] \\ &\le 2^n\,\mathbb{E}[\|\Phi(\rho) - \Phi(\sigma)\|_2^2] \\ &= \sum_{P\in\{I,X,Y,Z\}^{\otimes n}} \mathbb{E}[\mathrm{Tr}(P(\Phi(\rho) - \Phi(\sigma)))^2] \\ &\le 4\sum_{P\in\{I,X,Y,Z\}^{\otimes n}} c^{|P|+L-1} \\ &= 4(1+3c)^n c^{L-1}.\end{aligned} \tag{97}$$

In the first step, we have used Jensen's inequality, in the second step we have used the fact that $\|\cdot\|_1 \le 2^{\frac{n}{2}}\|\cdot\|_2$, and in the third step we expressed $\Phi(\rho) - \Phi(\sigma)$ in the Pauli basis and used the fact that $\|A\|_2^2 = \mathrm{Tr}\big(A^\dagger A\big)$ for any matrix A. This, in particular, implies that

$$\|A\|_2^2 = \frac{1}{2^n}\sum_{P\in\{I,X,Y,Z\}^{\otimes n}} \mathrm{Tr}(PA)^2 \tag{98}$$

for every Hermitian matrix A. Then, in the fourth step, we have used Proposition 16, and in the final step the binomial theorem. Finally, from the fact that $1 + 3c \le 4$, on account of the fact that $c \le 1$, we obtain

$$\mathbb{E}[\|\Phi(\rho) - \Phi(\sigma)\|_1] \le 2(1+3c)^{\frac{n}{2}}c^{\frac{L-1}{2}} \le 2^{n+1}c^{\frac{L-1}{2}}. \tag{99}$$

The right-most expression in the above chain of inequalities is bounded from above by ε if

$$L \ge \frac{1}{\log(c^{-1})}\left(2n + 2\log\left(\frac{1}{\varepsilon}\right) + 3\right), \tag{100}$$

which implies the desired result. □

The previous proposition implies that for most of noisy circuit of depth larger than $L \geq \Omega(n)$, the trace distance between the two output states is bounded from above by $\exp(-\Theta(n))$, which means that the two output states cannot be distinguished between each other efficiently by performing arbitrary measurements on polynomially many copies of the state, because of the Holevo-Helstrom theorem [68]. It seems plausible that the previous assumption on the depth $L \geq \Omega(n)$ is an artifact of our proof technique, and we conjecture that this assumption might be relaxed to smaller depths with a more fine-grained analysis; we leave this for future work.

One may wonder if it is possible to prove an upper bound on the worst-case trace distance (i.e., with no expected value) that decreases exponentially with the number of layers. Although this is possible by making strong structural assumption on the noise, namely being unital and with the maximally mixed state being their unique fixed point (e.g., depolarizing noise) [52, 53]; this is not possible for arbitrary noise regime in general. In fact, as an easy example, if the noise is dephasing, the circuit is made only by Toffoli gates, and the input states ρ and σ are two different computational basis states, then the trace distance between the two output states must remain constant for any depth (since Toffoli maps computational basis states in computational basis states and dephasing noise acts trivially on computational basis states). More interestingly, the so-called *quantum refrigerator* construction [14] shows surprisingly how non-unital noise can be exploited to perform fault-tolerant quantum computations in a model similar to ours, up to exponential depth. Therefore, for these special classes of circuits, the trace distance remains of constant order. However, we show below that in a certain high noise regime, we can find a worst-case upper bound on the trace distance that decays exponentially in the number of qubits.

2. *Worst-case upper bound on the trace distance*

In this section we give a worst-case bound for the trace distance that holds whenever the noise parameters exceed certain thresholds. We first introduce a some technical tools before proving our worst-case trace distance upper bound. Our argument is based on the contraction coefficients of the quantum Wasserstein distance of order 1 (W_1 distance) [16].

Let $O_n^T \subset \mathcal{L}(\mathbb{C}^{2^n})$ be the subset of traceless self-adjoint linear operators. The W_1 distance is induced by the quantum W_1 norm, which is defined as follows [16]:

$$\|X\|_{W_1} = \frac{1}{2}\min\left\{\sum_{i=1}^{n}\|X^{(i)}\|_1 : X^{(i)} \in O_n^T, \operatorname{Tr}_i X^{(i)} = 0, X = \sum_{i=1}^{n} X^{(i)}\right\}. \tag{101}$$

Hence, for two arbitrary states ρ, σ, the W_1 distance is defined as

$$W_1(\rho,\sigma) := \|\rho - \sigma\|_{W_1}. \tag{102}$$

The quantum W_1 norm and the trace norm are always within a factor of n, since

$$\frac{1}{2}\|X\|_1 \leq \|X\|_{W_1} \leq \frac{n}{2}\|X\|_1. \tag{103}$$

We will employ the *contraction coefficient* of a channel Φ with respect to the quantum W_1 distance, defined as

$$\|\Phi\|_{W_1\to W_1} := \max_{\rho\neq\sigma\in\mathcal{S}(\mathbb{C}^{2^n})} \frac{\|\Phi(\rho)-\Phi(\sigma)\|_{W_1}}{\|\rho-\sigma\|_{W_1}} = \max_{\substack{X\in O_n^T,\\ \|X\|_{W_1}=1}} \|\Phi(X)\|_{W_1}. \tag{104}$$

The contraction coefficient is not in general bounded by 1, as the W_1 does not satisfy a data-processing inequality for all channels. Importantly, as showed in Ref. [16], if Φ is a layer of k-qubit gates, the contraction coefficient of Φ can be bounded by light-cone argument as follows

$$\|\Phi\|_{W_1\to W_1} \leq \begin{cases} 1 & \text{if } k=1, \\ \frac{3}{2}k & \text{if } k>1 \ \text{ ([16], Proposition 13).}\end{cases} \tag{105}$$

And thus a layer of two qubit gates has contraction coefficient at most 3. If $\mathcal{N}$ is a single-qubit channel, the contraction coefficient of the tensor power channel $\mathcal{N}^{\otimes n}$ can be upper bounded by the diamond distance between $\mathcal{N}$ and a suitable 1-qubit channel $\mathcal{E}$ [16], as follows.

Proposition 20 (Proposition 11, [16])**.** *Let Φ be a single qubit quantum channel with fixed point a quantum state τ and let $\mathcal{E}$ the single-qubit quantum channel that replaces any state with τ. Then,*

$$\frac{1}{2}\|\Phi-\mathcal{E}\|_{1\to1} \leq \|\Phi^{\otimes n}\|_{W_1\to W_1} \leq \|\Phi-\mathcal{E}\|_{\diamond} \leq 2\|\Phi-\mathcal{E}\|_{1\to1}, \tag{106}$$

where we recall that for any single-qubit Hermitian-preserving linear map $\mathcal{F}$,

$$\|\mathcal{F}\|_{1\to 1} = \max_{\rho\in\mathcal{S}(\mathbb{C}^2)} \|\mathcal{F}(\rho)\|_1, \tag{107}$$

$$\|\mathcal{F}\|_{\diamond} = \max_{\rho\in\mathcal{S}(\mathbb{C}^2\otimes\mathbb{C}^2)} \|\mathcal{F}\otimes\mathcal{I}(\rho)\|_1. \tag{108}$$

By exploiting the above result, we give an explicit upper bound of the contraction coefficient in terms of the parameters of the noise channel $\mathcal{N}$ expressed in the normal form. We remark that the adoption of the normal form comes without loss of generality: as discussed in Section I C 2, we can always write a single-qubit channel $\mathcal{M}$ as $\mathcal{M}(\cdot) = U\mathcal{N}(V^\dagger(\cdot)V)U^\dagger$, where U, V are suitable single-qubit unitaries. By Eq. (105), U and V do not alter the W_1 norm, thus they can be neglected in our analysis.

Lemma 21. *Let $\mathcal{N}$ be a single-qubit channel that acts as $\mathcal{N}(I + \mathbf{w}\cdot\boldsymbol{\sigma}) = I + (\mathbf{t} + D\mathbf{w})\cdot\boldsymbol{\sigma}$. Then, $\mathcal{N}$ has a unique fixed point and it satisfies*

$$\|\mathcal{N}^{\otimes n}\|_{W_1\to W_1} \le \max_{P\in\{X,Y,Z\}} \frac{2D_P}{1-D_P}\|\mathcal{N}-\mathcal{I}\|_{1\to 1}. \tag{109}$$

Proof. A fixed point $\tau = (I + \omega\cdot\boldsymbol{\sigma})/2$ of $\mathcal{N}$ should satisfy $\mathcal{N}(\tau) = \tau$, where $\omega = (\omega_X, \omega_Y, \omega_Z)$ is a unit vector in $\mathbb{R}^3$. By definition of the channel, this implies that $(\mathbf{t} + D\omega)\cdot\boldsymbol{\sigma} = \omega\cdot\boldsymbol{\sigma}$. Thus, we have

$$t_P + D_P\omega_P = \omega_P, \tag{110}$$

for all $P \in \{X, Y, Z\}$. From which follows that τ is a fixed point of $\mathcal{N}$ if and only if $\omega_P = \frac{t_P}{1-D_P}$ for all $P \in \{X, Y, Z\}$. Thus, the only fixed state of $\mathcal{N}$ is

$$\tau = \frac{I}{2} + \sum_{P\in\{X,Y,Z\}} \frac{t_P}{2(1-D_P)} P. \tag{111}$$

We consider a single-qubit state parametrized as

$$\rho = \frac{I}{2} + \frac{1}{2}\sum_{P\in\{X,Y,Z\}} w_P P, \tag{112}$$

where $\mathbf{w} = (w_X, w_Y, w_Z)$ is a unit vector in $\mathbb{R}^3$. We will upper bound the Schatten 1-distance between $\mathcal{N}(\rho)$ and τ. To this end, we will employ the fact that the 1-distance and the 2-distance coincide up to a factor $\sqrt{2}$ for single-qubit states. We have

$$\begin{aligned}
\|\mathcal{N}(\rho) - \tau\|_1 &= \sqrt{2}\|\mathcal{N}(\rho) - \tau\|_2 \\
&= \sqrt{\sum_{P\in\{I,X,Y,Z\}} \mathrm{Tr}[P(\mathcal{N}(\rho)-\tau)]^2} \\
&= \sqrt{\sum_{P\in\{X,Y,Z\}} \left(t_P + D_P w_P - \frac{t_P}{1-D_P}\right)^2} \\
&= \sqrt{\sum_{P\in\{X,Y,Z\}} \left(\frac{D_P}{1-D_P}\right)^2 (w_P - (w_P D_P + t_P))^2} \\
&\le \max_{Q\in\{X,Y,Z\}} \left|\frac{D_Q}{1-D_Q}\right| \times \sqrt{\sum_{P\in\{X,Y,Z\}} (w_P - (w_P D_P + t_P))^2} \\
&= \max_{Q\in\{X,Y,Z\}} \left|\frac{D_Q}{1-D_Q}\right| \times 2\|\rho - \mathcal{N}(\rho)\|_1,
\end{aligned} \tag{113}$$

where in the only inequality we upper bounded each $\left(\frac{D_P}{1-D_P}\right)^2$ with $\max_{Q\in\{X,Y,Z\}}\left(\frac{D_Q}{1-D_Q}\right)^2$. Hence, the contraction coefficient of the quantum W_1 distance can be bounded via Proposition 20 as

$$\|\mathcal{N}^{\otimes n}\|_{W_1\to W_1} \le 2\|\mathcal{N}-\mathcal{E}\|_{1\to 1} = 2\max_{\rho\in\mathcal{S}} \|\mathcal{N}(\rho) - \tau\|_1 \tag{114}$$

$$\leq 2 \max_{\rho \in \mathcal{S}} \max_{P\in\{X,Y,Z\}} \frac{2D_P}{1-D_P} \|\rho - \mathcal{N}(\rho)\|_1$$
$$= \max_{P\in\{X,Y,Z\}} \frac{4D_P}{1-D_P} \|\mathcal{N} - \mathcal{I}\|_{1\to 1},$$

as required. □

The above bound applies to any local noise channel expressed in its normal form. This can be used to argue that, if the noise strength exceeds a given threshold, we witness a logarithmic effective depth for any fixed circuit. This results complements the findings of the previous section, which held for average-case circuits. The following proof extends to the non-unital case a result (Proposition IV.8) that was proven in Ref. [69] for the case of local depolarizing noise.

Proposition 22. *Let $\mathcal{N}$ be a single-qubit channel that acts as $\mathcal{N}(I+\mathbf{w}\cdot\boldsymbol{\sigma}) = I+(\mathbf{t}+D\mathbf{w})\cdot\boldsymbol{\sigma}$, and let $b := 24 \max_{P\in\{X,Y,Z\}} \frac{D_P}{1-D_P}$. Let Φ be a noisy quantum circuit with depth L. Then, the W_1 distance between $\Phi(\rho)$ and $\Phi(\sigma)$, where ρ and σ are two arbitrary quantum states, decays exponentially in L as*

$$\|\Phi(\rho) - \Phi(\sigma)\|_{W_1} \leq b^L \|\rho - \sigma\|_{W_1}. \tag{115}$$

Furthermore, we have the upper bound

$$\|\Phi(\rho) - \Phi(\sigma)\|_1 \leq n b^L \|\rho - \sigma\|_1 \tag{116}$$

on the trace distance. Thus, for any $\varepsilon > 0$, assuming that $b < 1$ (that is, $D_P < \frac{1}{25}$ for all $P \in \{X,Y,Z\}$) and $L \geq \frac{1}{\log(b^{-1})}\Omega\big(\log\big(\frac{n}{\varepsilon}\big)\big)$, we have that $\|\Phi(\rho) - \Phi(\sigma)\|_1 \leq \varepsilon$.

Proof. Let $\mathcal{U} = U(\cdot)U^\dagger$ be a layer of 2-qubit unitaries. Then, by Eq. (105), we have $\|\mathcal{U}\|_{W_1\to W_1} \leq 3$. Moreover, Lemma 21 yields

$$\begin{aligned} \|\mathcal{U} \circ \mathcal{N}^{\otimes n}\|_{W_1\to W_1} &\leq \|\mathcal{U}\|_{W_1\to W_1} \|\mathcal{N}^{\otimes n}\|_{W_1\to W_1} \\ &\leq 3 \max_{P\in\{X,Y,Z\}} \frac{4D_P}{1-D_P} \|\mathcal{N} - \mathcal{I}\|_{1\to 1} \\ &\leq 24 \max_{P\in\{X,Y,Z\}} \frac{D_P}{1-D_P} \\ &= b, \end{aligned} \tag{117}$$

where in the first inequality we have used the submultiplicativity property of $\|\cdot\|_{W_1\to W_1}$, in the third inequality we have used the fact that $\|\mathcal{N} - \mathcal{I}\|_{1\to 1} \leq 2$, due to the triangle inequality. Iterating over all the layers of the noisy circuit Φ, we obtain

$$\|\Phi\|_{W_1\to W_1} \leq b^L, \tag{118}$$

which directly implies Eq. (115). Furthermore, Eq. (116) follows from the fact that the W_1 distance and the trace distance are within a factor of n, i.e., $\frac{1}{2}\|X\|_1 \leq \|X\|_{W_1} \leq \frac{n}{2}\|X\|_1$. □

In particular, assuming that b is a constant less than one (that is, $D_P < \frac{1}{25}$ for all $P \in \{X,Y,Z\}$), we have

$$\|\Phi(\rho - \sigma)\|_1 \leq n 2^{-\Omega(L)}. \tag{119}$$

E. Classical simulation of Pauli expectation values of noisy random quantum circuits

In this section, we address the problem of estimating Pauli expectation values of noisy random quantum circuits under arbitrary noise. Specifically, given an instance of a noisy circuit Φ in which the two-qubit gates are sampled uniformly at random within a fixed architecture, our goal is to estimate $\mathrm{Tr}(P\Phi(\rho_0))$ to accuracy ε with high probability over the choice of the random circuit, where P is a prescribed Pauli operator and ρ_0 is an initial state. Without loss of generality, we focus on the case where the Pauli operator is local, since contributions from large Pauli weights are exponentially suppressed and, therefore, negligible.

We have seen that the presence of any non-unitary noise in the circuit renders the circuit effectively shallow for the purpose of estimating expectation values. In particular, for any inverse polynomial precision, the last logarithmically-many layers suffice. Specifically, a direct consequence of Proposition 16 implies the following:

Corollary 23 (Effective-depth picture)**.** *Let $P \in \{I, X, Y, Z\}^{\otimes n}$, and ρ_0 any input state. Let L be depth of the noisy circuit Φ. Then, we have*

$$\mathbb{E}_{\Phi_{[L-m,L]}}[|\operatorname{Tr}(P\Phi(\rho_0)) - \operatorname{Tr}\big(P\Phi_{[L-m,L]}(\sigma_0)\big)|^2] \leq 4c^{|P|+m-1}, \tag{120}$$

where σ_0 is any preferred state (e.g., $\sigma_0 := |0^n\rangle\langle 0^n|$). Here, $\Phi_{[L-m,L]}(\cdot)$ refers to the noisy circuit where only the last m layers are considered.

This yields the following simple algorithm for estimating local expectation values: work in the Heisenberg picture and 'propagate' the local Pauli P only a few number of layers backwards; compute classically the matrix $P_m := \Phi^*_{[L-m,L]}(P)$, and then evaluate $\operatorname{Tr}(P_m\sigma_0)$. Due to standard light-cone arguments, for any product state σ_0 (e.g., $\sigma_0 := |0^n\rangle\langle 0^n|$), the time-complexity of this algorithm is exponential in the number of qubits over which P_m is supported. If the circuit is a D-dimensional geometrical local circuit, P_m is supported on at most $|P|(2m)^{\mathrm{D}}$. If the circuit architecture instead does not possess any geometrical locality, i.e., it has all-to-all connectectivity, then P_m is supported on at most $|P|2^m$. Thus, for D-dimensional geometrical local circuit architectures, this algorithm incurs a total time complexity bounded from above by $\exp\big(O(|P|m^{\mathrm{D}})\big)$, while for all-to-all connected architectures, the time complexity is $\exp(O(|P|2^m))$. We refer to Sec. I A for a formal definition of the light-cone of an observable and geometrical locality.

Proposition 24 (Average classical simulation of local expectation values)**.** *Let $\varepsilon, \delta > 0$. Consider a Pauli operator P and any initial state ρ_0. For a noisy quantum circuit Φ of depth L, sampled according to the described circuit distribution, there exists a classical algorithm that outputs a value $\hat{C}$ satisfying*

$$|\hat{C} - \operatorname{Tr}(P\Phi(\rho_0))| \leq \varepsilon \tag{121}$$

with success probability at least $1-\delta$ over the choice of the random circuit. Specifically, the classical algorithm involves computing $\hat{C} := \operatorname{Tr}\big(P\Phi_{[L-m,L]}(|0^n\rangle\langle 0^n|)\big)$ with

$$m := \left\lceil \frac{1}{\log(c^{-1})} \log\left(\frac{4}{\delta\varepsilon^2}\right)\right\rceil, \tag{122}$$

where c is the noise parameter defined in Lemma 6. The time complexity of this algorithm is given by

$$\textit{Runtime} \leq \begin{cases} \exp\big(O(|P|m^{\mathrm{D}})\big) = \exp\Big(O\Big(\log^{\mathrm{D}}(\varepsilon^{-1})\Big)\Big), & \textit{for D-geometrically-local architectures,} \\ \exp(|P|\exp(O(m))) = \exp\big(\mathrm{poly}(\varepsilon^{-1})\big), & \textit{for all-to-all connected architectures.} \end{cases} \tag{123}$$

where in the last equation we assumed constant noise rate c, constant failure probability δ and $|P| = O(1)$.

Proof. Because of the Markov inequality, we have

$$\begin{aligned} \operatorname{Prob}\big(|\operatorname{Tr}(P\Phi(\rho_0)) - \operatorname{Tr}\big(P\Phi_{[L-m,L]}(\rho_0)\big)| > \varepsilon\big) &\leq \frac{1}{\varepsilon^2}\mathbb{E}[|\operatorname{Tr}(P\Phi(\rho_0)) - \operatorname{Tr}\big(P\Phi_{[L-m,L]}(\rho_0)\big)|^2] \\ &\leq \frac{4}{\varepsilon^2}c^m, \end{aligned} \tag{124}$$

where we have used Corollary 23 with $|P| \geq 1$. The right-hand side of this inequality is at most δ if we choose

$$m = \lceil \frac{1}{\log(c^{-1})} \log\Big(\frac{4}{\delta\varepsilon^2}\Big)\rceil. \tag{125}$$

The algorithm consists of computing classically the matrix $P_m := \Phi^*_{[L-m,L]}(P)$ and then evaluate $\operatorname{Tr}(P_m\rho_0)$. As we have previously described, this can be done via standard light-cone arguments, with a time complexity exponential in the number of qubits over which P_m is supported. □

Note that for one-dimensional circuits $\mathrm{D} = 1$ and local Pauli, the runtime depends only polynomially by ε^{-1}, while for higher dimension the time complexity depends quasi-polynomially by ε^{-1}.

Moreover, if $\Phi^*_{[L-m,L]}(P)$ is close to something proportional to the identity (which can be verified classically with the same time-complexity of above), then we can certify that our algorithm has succeeded. The intuition about this is that if $\Phi^*_{[L-m,L]}(P)$ were proportional to the identity, then keeping adding (adjoint) layers does not change the matrix because of the unitality of the adjoint channel. Specifically, at the end of the previous algorithm, we can check (efficiently in the effective dimension of the propagated observable) if the condition

$$E := \min_{q\in\mathbb{R}} \left\|\Phi^*_{[L-m,L]}(P) - qI\right\|_\infty \leq \varepsilon/2 \tag{126}$$

is satisfied. If it is, then the previous algorithm succeeded with unit probability, as we are going to show in the next observation.

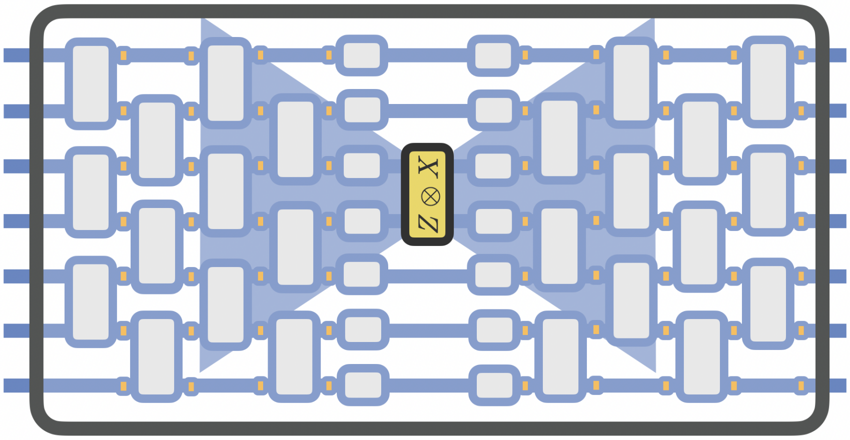

Figure 2. A graphical representation of $\Phi^*_{[L-2,L]}(P)$ with respect to the local Pauli observable P represented by the blue shaded area. The (noisy) gates outside the blue shaded area are contracted trivially due to the fact that the adjoint of every channel is unital, and thus cannot influence the expectation value of the Pauli. Even if the qubits in the system are n, the computation of $\Phi^*_{[L-2,L]}(P)$ is restricted to only a constant number of qubits.

Observation 25 (Verification guarantees)**.** *After running the algorithm described in Proposition 24, if such condition is true:*

$$E := \min_{q\in\mathbb{R}} \left\|P_m - qI\right\|_\infty \leq \varepsilon/2, \tag{127}$$

where $P_l := \Phi^*_{[L-m,L]}(P)$, then we can conclude that the algorithm in Proposition 24 succeeded with unit probability. Furthermore, such condition can be verified with the same time complexity of the algorithm in Proposition 24. In particular, it holds that

$$E = \frac{1}{2}\left(\lambda_{\max}(P_m) - \lambda_{\min}(P_m)\right), \tag{128}$$

where $\lambda_{\min}(P_m)$ and $\lambda_{\max}(P_m))$ are respectively the minimum and the maximum eigenvalue of P_m.

Proof. Assuming that $E := \min_{q\in\mathbb{R}} \left\|P_m - qI\right\|_\infty \leq \varepsilon/2$, we have

$$\begin{aligned} |\operatorname{Tr}(P\Phi(\rho_0)) - \operatorname{Tr}\big(P\Phi_{[L-m,L]}(\rho_0)\big)| &\leq |\operatorname{Tr}(P\Phi(\rho_0)) - q| + |q - \operatorname{Tr}\big(P\Phi_{[L-m,L]}(\rho_0)\big)| \\ &= |\operatorname{Tr}\big(P_l\Phi_{[1,L]}(\rho_0)\big) - q| + |\operatorname{Tr}(P_m\rho_0) - q| \\ &= |\operatorname{Tr}\big((P_m - qI)\Phi_{[1,L]}(\rho_0)\big)| + |\operatorname{Tr}((P_m - qI)\rho_0)| \\ &\leq \left\|P_m - qI\right\|_\infty + \left\|P_m - qI\right\|_\infty \\ &\leq \varepsilon, \end{aligned} \tag{129}$$

where in the second to last step we have used Hölder inequality. This shows that if $E \leq \varepsilon/2$, then the algorithm in Proposition 24 succeeds with unit probability.

Moreover, E can be computed in polynomial time in the effective dimension of the observable P_m. This can be seen for example by noting that E depends only by the eigenvalues of P_l, since it suffices to compute its spectrum. In particular, we have

$$\min_{q\in\mathbb{R}} \left\|P_m - qI\right\|_\infty = \min_{q\in\mathbb{R}} \max(|\lambda_{\min}(P_m) - q|, |\lambda_{\max}(P_m) - q|) = \frac{1}{2}\left(\lambda_{\max}(P_m) - \lambda_{\min}(P_m)\right). \tag{130}$$

This completes the proof. □

We note that such verification step can be also inserted at each step in which we take the adjoint of each of the last unitary layer. We refer to Algorithm 1 for a summary of the described steps.

Algorithm 1 Computing local expectation values on noisy circuit

Parameters: $\varepsilon, \delta > 0$ (desired precision and success probability).
Input: Classical descriptions of $\mathcal{C}$ (noiseless circuit), noise channel $\mathcal{N}$ with parameter c and observable of interest P.

Initialize: $P_0 = P$.
$l := \lceil \frac{1}{\log(c^{-1})} \log\left(\frac{4}{\delta\varepsilon^2}\right) \rceil$.
for $t = 1$ to m, **do**
    $P_t \leftarrow \Phi^*_{[L-t,L]}(P_{t-1})$.
    $E_t \leftarrow \frac{1}{2}\left(|\lambda_{\max}(P_t) - \lambda_{\min}(P_t)|\right)$ ▷ Check early-break condition
    if $2E_t \leq \varepsilon$ **then**
        Output $\mathrm{Tr}(P_t \,|0^n\rangle\langle 0^n|)$.
        Break
    end if
end for
Output $\mathrm{Tr}(P_m \,|0^n\rangle\langle 0^n|)$

The above algorithm is efficient if $|P| = O(\log(n))$ (i.e., its time complexity runs polynomially in the number of qubits), and it is no longer efficient if $|P| = \omega(\log(n))$. However, in this high Pauli-weight regime, we do not need to run any algorithm, since we can just output zero and this succeeds with high probability over the choice of the circuit and with an inverse-polynomial accuracy, due to the following observation.

Observation 26 (Output zero if the Pauli is Global)**.** *Let $\varepsilon = \Theta(1/\mathrm{poly}(n))$, $P \in \{I, X, Y, Z\}^{\otimes n}$, ρ_0 an arbitrary initial state, and let Φ be a noisy quantum circuit of any depth sampled according to the described circuit distribution. If $|P| = \omega(\log(n))$, then the probability that expectation value $\mathrm{Tr}(P\Phi(\rho_0))$ is larger than ε is negligible:*

$$\mathrm{Prob}\left(|\,\mathrm{Tr}(P\Phi(\rho_0))| \geq \varepsilon\right) \leq \mathrm{negl}(n), \tag{131}$$

where $\mathrm{negl}(n)$ denotes a negligible function, i.e., a function that grows more slowly than any inverse polynomial in the number of qubits n.

Proof. Recalling that $\mathbb{E}[\mathrm{Tr}(P\Phi(\rho_0))] = 0$, the Chebyshev inequality implies that

$$\mathrm{Prob}\left(|\,\mathrm{Tr}(P\Phi(\rho_0))| \geq \varepsilon\right) \leq \frac{1}{\varepsilon^2}\mathrm{Var}\left[\mathrm{Tr}(P\Phi(\rho_0))\right] \leq \frac{c^{|P|}}{\varepsilon^2}, \tag{132}$$

where we have used Proposition 14. If ε is at most inverse-polynomially small and $c^{|P|}$ with $|P| = \omega(\log(n))$ is super-polynomially small, then $c^{|P|}/(\varepsilon^2)$ will be negligible. □

Taken together, the results of this subsection give a classical simulation algorithm for estimating Pauli expectation values of (possibly non-unital) noisy random quantum circuits. If the required precision is constant in the number of qubits n, then the running time of the algorithm is efficient for any circuit architecture. If the required precision scales inverse-polynomially, then the algorithm runs in polynomial time for 1-D architectures, while in quasi-polynomial time in higher constant dimensionality (e.g., 2-D).

F. Classical simulation via depth- and Pauli-weight-truncated light cones

In the previous section we introduced an algorithm that, for any fixed architecture in constant dimension D and any initial state ρ_0, has a runtime of $\exp\big(O(\log(\varepsilon^{-1}))^D\big)$, where ε is the desired accuracy of the system, whereas the runtime for all-to-all architectures was $\exp\big(O(\varepsilon^{-1})\big)$. Thus, this algorithm is always efficient if the required precision is constant with respect to the number of qubits n. However, if the desired precision scales inverse-polynomially with n, the algorithm has a runtime of $\exp\big(O(\log(n))^D\big) = n^{O(\log(n))^{D-1}}$ for any constant dimension D, and exponential time for all-to-all connected architectures.

Here, we present an algorithm that improves upon these runtimes. Specifically, the runtime of this algorithm is $n^{O((D-1)\,\mathrm{loglog}(n))}$ for geometrically local architectures in any dimension D, and $n^{O(\log(n))}$ for all-to-all connected architectures, independent of the depth and initial state. Since $\mathrm{loglog}\, n$ is extremely small for practically relevant n, the first runtime can be effectively regarded as polynomial in practice. The idea is simple: in addition to retaining only the last few layers and applying a light-cone argument as in the previous section, we can further restrict attention to Pauli strings of small weight inside the light cone. As we have already shown that high-weight Pauli terms contribute exponentially little to the expectation value, they can be safely discarded. Specifically, we combine the theorems proven in the previous section regarding effective depth and exponential suppression of Pauli weight with ideas from Ref. [70], originally developed for the noiseless case, and generalize them to the possibly noisy setting.

We first introduce some useful definitions and lemmas.

Definition 27 (Truncation map)**.** *We define the truncation map $T^{(>k)}: \mathcal{B}(\mathcal{H}_n) \to \mathcal{B}(\mathcal{H}_n)$ as the linear map that acts on any Pauli basis element $P \in \{I, X, Y, Z\}^{\otimes n}$ as follows:*

$$T^{(>k)}(P) := \begin{cases} P & \text{if } |P| \leq k, \\ 0 & \text{if } |P| > k, \end{cases} \tag{133}$$

where $|P|$ denotes the Pauli weight of P and $k \in \mathbb{R}$ is a specified threshold. This definition extends to possibly non-Pauli operators by linearity.

It is important to observe a few properties of the truncation map.

Lemma 28 (Basic properties of the truncation map)**.** *We have the following.*

1. $\|T^{(>k)}(O)\|_2 \leq \|O\|_2$*, for any observable O.*
2. *The truncation map commutes with any layer of single-qubit Clifford gates, i.e.,*
$$\mathcal{C}^{\text{single}} \circ T^{(>k)} = T^{(>k)} \circ \mathcal{C}^{\text{single}}, \tag{134}$$
where $\mathcal{C}^{\text{single}} := C(\cdot)C^{\dagger}$, with $C := \bigotimes_{i=1}^{n} C_i$, is a layer of single-qubit Clifford gates.

Proof. The inequality $\|T^{(>k)}(O)\|_2 \leq \|O\|_2$ can be shown by expanding O in the Pauli basis. The commutativity property can be verified by explicitly applying the linear maps on both sides to an operator expanded in the Pauli basis, and using that single qubits Clifford layers do not change the Pauli weight of a given Pauli. □

We now present a Lemma, which is shown in Ref. [71].

Lemma 29 (Low-degree approximation, Corollary 13 in Ref. [71] (Restated))**.** *Let O be an operator and $k \in \mathbb{R}$. Let $\mathcal{D}$ be a distribution over quantum states that is invariant under single-qubit Clifford gates. Then, we have*

$$\mathbb{E}_{\rho\sim\mathcal{D}} \left|\operatorname{Tr}(O\rho) - \operatorname{Tr}\left(O^{(k)}\rho\right)\right|^2 \leq \left(\frac{2}{3}\right)^k \frac{\|O\|_2^2}{2^n}, \tag{135}$$

where $O^{(k)} := T^{(>k)}(O)$.

Lemma 30 (Linear map 2-norm bound)**.** *Let ν be a distribution over the unitary group, satisfying*

$$\forall P, Q \in \{I, X, Y, Z\}^{\otimes n} \text{ such that } P \neq Q : \mathbb{E}_{U\sim\nu}[U^{\dagger\otimes 2}(P \otimes Q)U^{\otimes 2}] = 0. \tag{136}$$

For any linear map Φ and any Hermitian operator O, we have

$$\mathbb{E}_{U\sim\nu}\|\Phi^{\dagger}(UOU^{\dagger})\|_2^2 \leq \left(\max_{s\in\mathcal{P}_n} \|\Phi^{\dagger}(s)\|_2^2\right) \|O\|_2^2. \tag{137}$$

In particular, if Φ is a quantum channel, we have

$$\mathbb{E}_{U\sim\nu}\|\Phi^{\dagger}(UOU^{\dagger})\|_2^2 \leq \|O\|_2^2. \tag{138}$$

Proof. Expanding O and $\Phi^{\dagger}(UOU^{\dagger})$ in the Pauli basis $\mathcal{P}_n := \left\{\frac{I}{\sqrt{2}}, \frac{X}{\sqrt{2}}, \frac{Y}{\sqrt{2}}, \frac{Z}{\sqrt{2}}\right\}^{\otimes n}$, we find

$$\begin{aligned} \mathbb{E}_{U\sim\nu}\|\Phi^{\dagger}(UOU^{\dagger})\|_2^2 &= \sum_{t\in\mathcal{P}_n} \mathbb{E}_{U\sim\nu}\left[\operatorname{Tr}\left(t\Phi^{\dagger}(UOU^{\dagger})\right)\right]^2 \\ &= \sum_{t\in\mathcal{P}_n} \mathbb{E}_{U\sim\nu}\left[\operatorname{Tr}\left(\Phi(t)UOU^{\dagger}\right)\right]^2 \\ &= \sum_{s,t\in\mathcal{P}_n} \operatorname{Tr}(s\Phi(t))^2 \mathbb{E}_{U\sim\nu}\left[\operatorname{Tr}\left(sUOU^{\dagger}\right)\right]^2 \\ &= \sum_{s\in\mathcal{P}_n} \left(\sum_{t\in\mathcal{P}_n} \operatorname{Tr}(s\Phi(t))^2\right) \mathbb{E}_{U\sim\nu}\left[\operatorname{Tr}\left(sUOU^{\dagger}\right)\right]^2 \end{aligned} \tag{139}$$

$$
\begin{aligned}
&\le \sum_{s\in\mathcal{P}_n}\left(\max_{s'\in\mathcal{P}_n}\sum_{t\in\mathcal{P}_n}\operatorname{Tr}(s'\Phi(t))^2\right)\mathbb{E}_{U\sim\nu}\left[\operatorname{Tr}\!\left(sUOU^\dagger\right)\right]^2\\
&\le \left(\max_{s'\in\mathcal{P}_n}\sum_{t\in\mathcal{P}_n}\operatorname{Tr}\!\left(\Phi^\dagger(s')t\right)^2\right)\sum_{s\in\mathcal{P}_n}\mathbb{E}_{U\sim\nu}\left[\operatorname{Tr}\!\left(sUOU^\dagger\right)\right]^2\\
&= \left(\max_{s'\in\mathcal{P}_n}\|\Phi^\dagger(s')\|_2^2\right)\mathbb{E}_{U\sim\nu}\|UOU^\dagger\|_2^2\\
&= \left(\max_{s\in\mathcal{P}_n}\|\Phi^\dagger(s)\|_2^2\right)\mathbb{E}_{U\sim\nu}\|O\|_2^2.
\end{aligned}
$$

Here, we have used Lemma 5 in the third step and the unitary invariance of the 2-norm in the last step. Therefore,

$$
\begin{aligned}
\mathbb{E}_{U\sim\nu}\|\Phi^\dagger(UOU^\dagger)\|_2^2 &= \left(\max_{s\in\mathcal{P}_n}\|\Phi^\dagger(s)\|_2^2\right)\|O\|_2^2 \qquad (140)\\
&\le \left(\max_{s\in\mathcal{P}_n}2^n\|\Phi^\dagger(s)\|_\infty^2\right)\|O\|_2^2\\
&= \left(\max_{P\in\{I,X,Y,Z\}^{\otimes n}}\|\Phi^\dagger(P)\|_\infty^2\right)\|O\|_2^2\\
&\le \left(\max_{P\in\{I,X,Y,Z\}^{\otimes n}}\|P\|_\infty^2\right)\|O\|_2^2\\
&= \|O\|_2^2,
\end{aligned}
$$

where in the second step we have used the fact that $\|A\|_2 \le \sqrt{\operatorname{rank}(A)}\|A\|_\infty$, in the fourth step we applied the Russo-Dye Theorem [7] (which states $\|\Phi^\dagger(A)\|_\infty \le \|A\|_\infty$ for any quantum channel Φ), and in the final step we noted that the operator norm of any Pauli matrix is 1. □

Note that if we remove the single-qubit random layers, the previous inequality becomes false, i.e., $\|\Phi^\dagger(O)\|_2 \le \|O\|_2$ does not hold in general, as can be verified by taking the single-qubit channel $\Phi(\cdot) := \operatorname{Tr}(\cdot)\,|0\rangle\!\langle 0|$ and $O := |0\rangle\!\langle 0|$ [72]. We now recall our definition of noisy random circuit.

Definition 31 (Noisy circuit model)**.** *We consider n-qubit quantum circuits Φ consisting of layers of two-qubit gates interleaved by local (single-qubit) noise, with a final layer of single-qubit gates. All gates are assumed to be drawn from a 2-design, and we make no assumptions about geometric locality, except where explicitly mentioned. We express our circuits as*

$$
\Phi := \mathcal{V}^{\mathrm{single}} \circ \mathcal{N}^{\otimes n} \circ \mathcal{U}_L \circ \cdots \circ \mathcal{N}^{\otimes n} \circ \mathcal{U}_1, \qquad (141)
$$

where $\mathcal{V}^{\mathrm{single}} := V(\cdot)V^\dagger$, with $V := \bigotimes_{i=1}^n U_i$, is a layer of single-qubit gates, L represents the number of layers (also referred to as circuit depth), $\mathcal{U}_i$ corresponds to the channel associated with the i-th unitary circuit layer for $i \in [L] := \{1, 2, \ldots, L\}$, and $\mathcal{N}$ is a single-qubit quantum channel.

Definition 32 (Weight and depth truncated adjoint circuit)**.** *Let $\Phi := \Phi_L \circ \cdots \circ \Phi_1$ be a noisy quantum circuit, where $\{\Phi_j\}_{j=1}^L$ represents the sequence of noisy circuit layers, as for the previous definition. Let $\Phi_{[L-m,L]}(\cdot)$ be the noisy circuit where only the last m layers are considered.*

We define the k-weight truncated adjoint circuit restricted to the last m-layers $(\Phi_{[L-m,L]})^\dagger_{k-\mathrm{trunc}}$ as

$$
(\Phi_{[L-m,L]})^\dagger_{k-\mathrm{trunc}} := (T^{(>k)} \circ \Phi^\dagger_{L-m}) \circ \cdots \circ (T^{(>k)} \circ \Phi^\dagger_L), \qquad (142)
$$

where $T^{(>k)}$ is the truncation map defined in Definition 27.

We are now ready to state the main results of this section. We begin with the following proposition, which combines the effective depth of noisy random circuits with Pauli-weight truncation. While the m factor multiplying the second term on the RHS can be removed using a refinement of the techniques in Ref. [70], this improvement does not change the asymptotic scaling of our classical simulation result and is therefore omitted here for simplicity.

Proposition 33 (Truncation in depth and weight approximation)**.** *Let ρ_0 be an initial state, O an observable, and $\Phi := \Phi_L \circ \cdots \circ \Phi_1$ a noisy quantum circuit, where $\{\Phi_j\}_{j=1}^L$ represents the sequence of noisy circuit layers as in Definition 31. Define*

$O_{\Phi}^{(k,m)} := (\Phi_{[L-m,L]})^{\dagger}_{k-\mathrm{trunc}}(O)$, *which represents the Heisenberg-evolved observable O with the last m noisy circuit layers k-weight truncated (layer-by-layer), as in Definition 32. We have*

$$\mathbb{E}_{\Phi}\left[\left|\mathrm{Tr}(O\Phi(\rho_0)) - \mathrm{Tr}\left(O_{\Phi}^{(k,m)}\sigma_0\right)\right|\right] \leq 2\|O\|_{\infty}\exp(-\alpha m) + m\left(\frac{2}{3}\right)^{k/2}\|O\|_{\infty}, \tag{143}$$

where σ_0 is any preferred initial state. Here, the average $\mathbb{E}_{\Phi}$ is taken with respect to the 2-design distribution of every two-qubit gate that composes the circuit, and $\alpha > 0$ is a quantity that depends only on the noise parameters.

Proof. We have

$$\begin{aligned}\mathbb{E}_{\Phi}\left[\left|\mathrm{Tr}(O\Phi(\rho_0)) - \mathrm{Tr}\left(O_{\Phi}^{(k,m)}\sigma_0\right)\right|\right] &\leq \mathbb{E}_{\Phi}\left[\left|\mathrm{Tr}(O\Phi(\rho_0)) - \mathrm{Tr}(O\Phi_{[L-m]}(\rho_0))\right|\right] + \mathbb{E}_{\Phi}\left[\left|\mathrm{Tr}(O\Phi_{[L-m]}(\rho_0)) - \mathrm{Tr}\left(O_{\Phi}^{(k,m)}\sigma_0\right)\right|\right]\\ &\leq 2\|O\|_{\infty}\exp(-\alpha m) + \mathbb{E}_{\Phi}\left[\left|\mathrm{Tr}(O\Phi_{[L-m]}(\rho_0)) - \mathrm{Tr}\left(O_{\Phi}^{(k,m)}\sigma_0\right)\right|\right],\end{aligned} \tag{144}$$

where we have used triangle inequality and Proposition 17. We now focus on the second term. We have

$$\begin{aligned}\mathbb{E}_{\Phi}\left[\left|\mathrm{Tr}(O\Phi_{[L-m]}(\rho_0)) - \mathrm{Tr}\left(O_{\Phi}^{(k,m)}\sigma_0\right)\right|\right] &= \mathbb{E}_{\Phi}\left[\left|\mathrm{Tr}(O\Phi_{[L-m]}(\rho_0)) - \mathrm{Tr}\left((\Phi_{[L-m,L]})^{\dagger}_{k-\mathrm{trunc}}(O)\rho_0\right)\right|\right]\\ &= \mathbb{E}_{\Phi}\left[\left|\mathrm{Tr}\left(\Phi^{\dagger}_{[L-m]}(O)\rho_0\right) - \mathrm{Tr}\left((\Phi_{[L-m,L]})^{\dagger}_{k-\mathrm{trunc}}(O)\rho_0\right)\right|\right]\\ &= \mathbb{E}_{\Phi}\left[\left|\mathrm{Tr}\left(\left(\Phi^{\dagger}_{[L-m]}(O) - (\Phi_{[L-m,L]})^{\dagger}_{k-\mathrm{trunc}}(O)\right)\rho_0\right)\right|\right].\end{aligned} \tag{145}$$

We notice that we can rewrite $\Phi^{\dagger}_{[L-m]}(O) - (\Phi_{[L-m,L]})^{\dagger}_{k-\mathrm{trunc}}(O)$ using a telescopic sum as

$$\begin{aligned}&\Phi^{\dagger}_{[L-m]}(O) - (\Phi_{[L-m,L]})^{\dagger}_{k-\mathrm{trunc}}(O)\\ &= \Phi^{\dagger}_{L-m}\circ\cdots\circ\Phi^{\dagger}_{L}(O) - (T^{(>k)}\circ\Phi^{\dagger}_{L-m})\circ\cdots\circ(T^{(>k)}\circ\Phi^{\dagger}_{L})(O)\\ &= \bigcirc_{a=L-m}^{L}\Phi^{\dagger}_{a}(O) - \bigcirc_{b=L-m}^{L}(T^{(>k)}\circ\Phi^{\dagger}_{b})(O)\\ &= \sum_{j=0}^{m-1}\left(\Phi^{\dagger}_{[L-m,L-j]}\circ\bigcirc_{b=L-j+1}^{L}(T^{(>k)}\circ\Phi^{\dagger}_{b})(O) - \Phi^{\dagger}_{[L-m,L-j-1]}\circ\bigcirc_{b=L-j}^{L}(T^{(>k)}\circ\Phi^{\dagger}_{b})(O)\right).\end{aligned} \tag{146}$$

Here, the notation $\bigcirc_{a=L-m}^{L}$ denotes the composition of maps from $a = L-m$ to L in the forward direction, while the notation $\Phi^{\dagger}_{[a,b]}$ with $a \leq b$ means $\Phi^{\dagger}_{a}\circ\cdots\circ\Phi^{\dagger}_{b}$. Substituting the telescoping sum into Eq. (145) and applying the triangle inequality, we get

$$\begin{aligned}&\mathbb{E}_{\Phi}\left[\left|\mathrm{Tr}(O\Phi_{[L-m]}(\rho_0)) - \mathrm{Tr}\left(O_{\Phi}^{(k,m)}\sigma_0\right)\right|\right]\\ &= \mathbb{E}_{\Phi}\left[\left|\mathrm{Tr}\left(\left(\Phi^{\dagger}_{[L-m]}(O) - (\Phi_{[L-m,L]})^{\dagger}_{k-\mathrm{trunc}}(O)\right)\rho_0\right)\right|\right]\\ &= \mathbb{E}_{\Phi}\left[\left|\sum_{j=0}^{m}\mathrm{Tr}\left(\left(\Phi^{\dagger}_{[L-m,L-j]}\circ\bigcirc_{b=L-j+1}^{L}(T^{(>k)}\circ\Phi^{\dagger}_{b})(O) - \Phi^{\dagger}_{[L-m,L-j-1]}\circ\bigcirc_{b=L-j}^{L}(T^{(>k)}\circ\Phi^{\dagger}_{b})(O)\right)\rho_0\right)\right|\right]\\ &\leq \sum_{j=0}^{m-1}\mathbb{E}_{\Phi}\left[\left|\mathrm{Tr}\left(\left(\Phi^{\dagger}_{[L-m,L-j]}\circ\bigcirc_{b=L-j+1}^{L}(T^{(>k)}\circ\Phi^{\dagger}_{b})(O) - \Phi^{\dagger}_{[L-m,L-j-1]}\circ\bigcirc_{b=L-j}^{L}(T^{(>k)}\circ\Phi^{\dagger}_{b})(O)\right)\rho_0\right)\right|\right]\\ &= \sum_{j=0}^{m-1}\mathbb{E}_{\Phi}\left[\left|\mathrm{Tr}\left(\left(\Phi^{\dagger}_{L-j}\circ\bigcirc_{b=L-j+1}^{L}(T^{(>k)}\circ\Phi^{\dagger}_{b})(O) - (T^{(>k)}\circ\Phi^{\dagger}_{L-j})\bigcirc_{b=L-j+1}^{L}(T^{(>k)}\circ\Phi^{\dagger}_{b})(O)\right)\Phi_{[L-m,L-j-1]}(\rho_0)\right)\right|\right]\\ &= \sum_{j=0}^{m-1}\mathbb{E}_{\Phi}\left[\left|\mathrm{Tr}\left(\left(\Phi^{\dagger}_{L-j}(O_{\Phi}^{(k,L-j+1)}) - (T^{(>k)}\circ\Phi^{\dagger}_{L-j})(O_{\Phi}^{(k,L-j+1)})\right)\Phi_{[L-m,L-j-1]}(\rho_0)\right)\right|\right]\\ &\leq \sum_{j=0}^{m-1}\left(\mathbb{E}_{\Phi}\left[\left|\mathrm{Tr}\left(\left(\Phi^{\dagger}_{L-j}(O_{\Phi}^{(k,L-j+1)}) - (T^{(>k)}\circ\Phi^{\dagger}_{L-j})(O_{\Phi}^{(k,L-j+1)})\right)\Phi_{[L-m,L-j-1]}(\rho_0)\right)\right|^2\right]\right)^{1/2},\end{aligned} \tag{147}$$

where in the last step we have used Jensen's inequality.

We now focus on each term in the sum. Since we are dealing with second moment quantities for each unitary layer, we can simplify the expression by adding independent single-qubit Clifford gates layers before and after each layer. Moreover, by Lemma 28 (point 2), the truncation map $T^{(>k)}$ commutes with any single-qubit Clifford layer. Thus, we the distribution associated to the state $\Phi_{[L-m,L-j-1]}(\rho_0)$ is invariant under single-qubit Clifford rotations. Applying Lemma 29, we obtain

$$\mathbb{E}_\Phi\left[\left|\mathrm{Tr}\Big(\Big(\Phi^\dagger_{L-j}(O_\Phi^{(k,L-j+1)}) - (T^{(>k)}\circ\Phi^\dagger_{L-j})(O_\Phi^{(k,L-j+1)})\Big)\,\Phi_{[L-m,L-j-1]}(\rho_0)\Big)\right|^2\right] \leq \left(\frac{2}{3}\right)^k \frac{1}{2^n}\mathbb{E}_\Phi\left\|O_\Phi^{(k,L-j+1)}\right\|_2^2. \tag{148}$$

Here, the expected value is taken only over the layers that appear in $O_\Phi^{(k,L-j+1)}$. We recall the definition of this term. For each $j\in\{1,\dots,m-1\}$, we have

$$\begin{aligned} O_\Phi^{(k,L-j+1)} &= (T^{(>k)}\circ\Phi^\dagger_{L-j})\circ\cdots\circ(T^{(>k)}\circ\Phi^\dagger_{L})(O) \\ &= T^{(>k)}\circ\Phi^\dagger_{L-j}(O_\Phi^{(k,L-j+2)}), \end{aligned} \tag{149}$$

where $O_\Phi^{(k,L+1)} := O$. Thus, we have

$$\begin{aligned} \mathbb{E}_\Phi\left\|O_\Phi^{(k,L-j+1)}\right\|_2^2 &= \mathbb{E}_\Phi\left\|T^{(>k)}\circ\Phi^\dagger_{L-j}(O_\Phi^{(k,L-j+1)})\right\|_2^2 \\ &\leq \mathbb{E}_\Phi\left\|\Phi^\dagger_{L-j}(O_\Phi^{(k,L-j-1)})\right\|_2^2 \\ &\leq \mathbb{E}_\Phi\left\|O_\Phi^{(k,L-j-2)}\right\|_2^2 \\ &\leq \|O\|_2^2, \end{aligned} \tag{150}$$

where in the second step we have used that the truncation map always contracts the Frobenius norm (Lemma 28), in the third step we have used the invariance of the circuit layer distribution under single-qubit Clifford layers and used Lemma 30, and in the last step we applied this argument for every layer. Thus, we obtain

$$\mathbb{E}_\Phi\left[\left|\mathrm{Tr}\Big(\Big(\Phi^\dagger_{L-j}(O_\Phi^{(k,L-j+1)}) - (T^{(>k)}\circ\Phi^\dagger_{L-j})(O_\Phi^{(k,L-j+1)})\Big)\,\Phi_{[L-m,L-j-1]}(\rho_0)\Big)\right|^2\right] \leq \left(\frac{2}{3}\right)^k \frac{1}{2^n}\|O\|_2^2. \tag{151}$$

Therefore, we get

$$\begin{aligned} \mathbb{E}_\Phi\left[\left|\mathrm{Tr}\big(O\Phi_{[L-m]}(\rho_0)\big) - \mathrm{Tr}\Big(O_\Phi^{(k,m)}\sigma_0\Big)\right|\right] &\leq \sum_{j=0}^{m-1}\left(\frac{2}{3}\right)^{k/2}\frac{1}{2^{n/2}}\|O\|_2 \\ &\leq m\left(\frac{2}{3}\right)^{k/2}\|O\|_\infty, \end{aligned} \tag{152}$$

where in the last step we have used the fact that $\|A\|_2 \leq \sqrt{\mathrm{rank}(A)}\|A\|_\infty$. Thus, we can conclude the proof. □

Corollary 34 (Error scaling). *Let $\varepsilon_A,\varepsilon_B>0$ be accuracy parameters and let $\delta\in(0,1)$ be the failure probability. Consider an initial state ρ_0, an observable O, and a noisy quantum circuit architecture $\Phi:=\Phi_L\circ\cdots\circ\Phi_1$, where $\{\Phi_j\}_{j=1}^L$ represents the sequence of noisy circuit layers. Here, $\alpha>0$ is a quantity that depends only on the noise parameters (see Proposition 17 for details). Define $O_\Phi^{(k,m)} := (\Phi_{[L-m,L]})^\dagger_{k\text{-trunc}}(O)$, which represents the Heisenberg-evolved observable O with the last m noisy circuit layers k-weight truncated (layer-by-layer), as described in Definition 32. Assume*

$$m := \left\lceil \alpha^{-1}\log\left(\frac{4\|O\|_\infty}{\delta\varepsilon_A}\right)\right\rceil,\ \ k := \left\lceil \frac{2}{\log(2/3)}\log\left(\frac{m\|O\|_\infty}{\delta\varepsilon_B}\right)\right\rceil, \tag{153}$$

where m and k satisfy $L\geq m$ and $n\geq k$ (i.e., the truncated depth is less than the entire circuit depth and the truncated weight is less than the number of qubits). Let σ_0 be any preferred initial state. Given a randomly chosen instance of the circuit Φ where the two-qubit gates in each layer are distributed according to a 2-design distribution, we can guarantee that the quantity $\mathrm{Tr}\Big(O_\Phi^{(k,m)}\sigma_0\Big)$ is close to the expectation value $\mathrm{Tr}(O\Phi(\sigma_0))$ by $\varepsilon_A+\varepsilon_B$, i.e.,

$$\left|\mathrm{Tr}(O\Phi(\rho_0)) - \mathrm{Tr}\Big(O_\Phi^{(k,m)}\sigma_0\Big)\right| \leq \varepsilon_A+\varepsilon_B, \tag{154}$$

with probability at least $1-\delta$ over the random choice of Φ.

Proof. By the previous theorem, with k and m chosen as defined, we have

$$\mathbb{E}_\Phi \left[\left| \mathrm{Tr}(O\Phi(\rho_0)) - \mathrm{Tr}\left(O_\Phi^{(k,m)}\sigma_0\right) \right| \right] \leq (\varepsilon_A + \varepsilon_B)\delta. \tag{155}$$

Applying Markov's inequality, we obtain

$$\mathrm{Prob}\left(\left| \mathrm{Tr}(O\Phi(\rho_0)) - \mathrm{Tr}\left(O_\Phi^{(k,m)}\sigma_0\right) \right| > \varepsilon_A + \varepsilon_B \right) \leq \frac{(\varepsilon_A + \varepsilon_B)\delta}{\varepsilon_A + \varepsilon_B} = \delta. \tag{156}$$

Therefore, with probability at least $1 - \delta$, the inequality

$$\left| \mathrm{Tr}(O\Phi(\rho_0)) - \mathrm{Tr}\left(O_\Phi^{(k,m)}\sigma_0\right) \right| \leq \varepsilon_A + \varepsilon_B \tag{157}$$

holds. This completes the proof. □

Remark 35. *If we set the accuracy $\varepsilon_A = \mathrm{poly}(n^{-1})$, $\varepsilon_B = \mathrm{poly}(n^{-1})$ and the failure probability $\delta = \mathrm{poly}(n^{-1})$, then the parameters m and k grow logarithmically with the number of qubits n, i.e., $m = O(\log(n))$ and $k = O(\log(n))$.*

Theorem 36 (Classical estimation of expectation values)**.** *Consider a quantum circuit Φ sampled uniformly at random with respect to a fixed architecture, which may include any type of local (non-unitary) noise and any number of layers. Given a Pauli operator $P \in \{I, X, Y, Z\}^{\otimes n}$ with Pauli weight $|P| = O(1)$, and an initial state ρ_0, we aim to estimate the expectation value $\mathrm{Tr}(P\Phi(\rho_0))$ with accuracy ε, where ε is an inverse-polynomial in n, specifically $\varepsilon = \mathrm{poly}(n^{-1})$.*

There exists a classical algorithm that accomplishes this estimation with a success probability of at least $1 - \delta$ over the choice of the random circuit Φ, where $\delta > 0$ is a constant or an inverse-polynomial in n. The runtime of this algorithm is:

- $n^{O((D-1)\,\mathrm{loglog}(n))}\mathrm{poly}(n)$ *for D-dimensional geometrically local architectures.*
- $n^{O(\log(n))}$ *for all-to-all connected architectures.*

Proof. To apply Corollary 34, we need to determine the time complexity of computing the estimator $\mathrm{Tr}\left(O_\Phi^{(k,m)}\sigma_0\right)$, where we choose σ_0 to be the zero state $|0^n\rangle\langle 0^n|$. Specifically, we first want to find the time complexity of evaluating $O_\Phi^{(k,m)}$, which is the Pauli P (with $|P| = O(1)$) Heisenberg-evolved with the last m layers of the circuit and k-truncated,

$$P_\Phi^{(k,m)} = (\Phi_{[L-m,L]})^\dagger_{k\text{-trunc}}(P) = (T^{(>k)} \circ \Phi_{L-m}^\dagger) \circ \cdots \circ (T^{(>k)} \circ \Phi_L^\dagger)(P) = T^{(>k)} \circ \Phi_{L-m}^\dagger(P_\Phi^{(k,L-m+1)}). \tag{158}$$

First, note that such Heisenberg-evolved local Pauli is supported on at most $n_{\mathrm{eff}} := |P|m^D$ qubits due to light-cone arguments, where D is the dimension of the circuit (for all-to-all connectivity, this would be $n_{\mathrm{eff}} := |P|2^m$). The operator $P_\Phi^{(k,m)}$ is supported only on Pauli operators in the light cone with Pauli weight at most k (this is true not only for $P_\Phi^{(k,m)}$ but also for $P_\Phi^{(k,1)}, \ldots, P_\Phi^{(k,m-1)}$). Let $\mathcal{P}_{\mathrm{light}}^{(\leq k)}$ denote this set of Pauli operators. The number of such Pauli operators is upper bounded by

$$|\mathcal{P}_{\mathrm{light}}^{(\leq k)}| = \sum_{w=0}^{k} \binom{n_{\mathrm{eff}}}{w} 3^w \leq \sum_{w=0}^{k} \frac{n_{\mathrm{eff}}^w}{w!} 3^w \leq n_{\mathrm{eff}}^k \sum_{w=0}^{k} \frac{3^w}{w!} \leq n_{\mathrm{eff}}^k \sum_{w=0}^{\infty} \frac{3^w}{w!} = e^3 n_{\mathrm{eff}}^k, \tag{159}$$

where e is the base of the natural logarithm.

The operator $T^{(>k)} \circ \Phi_L^\dagger(P) = T^{(>k)}(\Phi_L^\dagger(P))$ is supported in the light cone and, in particular, on Pauli operators with weight at most k. Thus, it can be expressed as

$$P_\Phi^{(k,1)} = T^{(>k)}(\Phi_L^\dagger(P)) = \frac{1}{d} \sum_{Q \in \{I,X,Y,Z\}^{\otimes n_{\mathrm{eff}}}} \mathrm{Tr}\left(Q\Phi_L^\dagger(P)\right) T^{(>k)}(Q) = \frac{1}{d} \sum_{Q \in \mathcal{P}_{\mathrm{light}}^{(\leq k)}} \mathrm{Tr}\left(Q\Phi_L^\dagger(P)\right) Q. \tag{160}$$

Since Φ_L is a circuit layer where each qubit is acted upon by at most one gate in Φ_L, the term $\mathrm{Tr}\left(Q\Phi_L^\dagger(P)\right)$ can be computed in $O(n)$ time, leveraging the tensor product structure of the Pauli matrices and the locality of the noise/gates. Therefore, computing all such coefficients $\mathrm{Tr}\left(Q\Phi_L^\dagger(P)\right)$ takes $O(n|\mathcal{P}_{\mathrm{light}}^{(\leq k)}|)$ time. We then evaluate

$$P_\Phi^{(k,2)} = \frac{1}{d} \sum_{Q \in \mathcal{P}_{\mathrm{light}}^{(\leq k)}} \mathrm{Tr}\left(Q\Phi_L^\dagger(P)\right) T^{(>k)}(\Phi_{L-1}^\dagger(Q)). \tag{161}$$

For each term $T^{(>k)}(\Phi_{L-1}^{\dagger}(Q))$, we repeat the previous process. Once computed, these terms can be rearranged in the Pauli basis of $\mathcal{P}_{\text{light}}^{(\leq k)}$ to find coefficients c_Q such that

$$P_{\Phi}^{(k,2)} = \sum_{Q \in \mathcal{P}_{\text{light}}^{(\leq k)}} c_Q Q. \tag{162}$$

This procedure takes $O(n|\mathcal{P}_{\text{light}}^{(\leq k)}|^2)$ time.

Repeating this procedure for all m layers results in an overall time complexity of $O(nm|\mathcal{P}_{\text{light}}^{(\leq k)}|^2)$. Now we can estimate for each of this Pauli the expectation value with the state $|0^n\rangle\langle 0^n|$, which takes additional $|\mathcal{P}_{\text{light}}^{(\leq k)}|$ time complexity.

For $m = O(\log(n))$ and $k = O(\log(n))$ (which is the interesting regime since the desired accuracy is inverse-polynomially small), we have

$$|\mathcal{P}_{\text{light}}^{(\leq k)}| \leq O(n_{\text{eff}}^k) = O(m^{Dk}) = O(\log(n)^{D\log(n)}) = n^{O(D\log(\log(n)))}. \tag{163}$$

The $D = 1$ case can be analyzed separately, yielding $|\mathcal{P}_{\text{light}}^{(\leq k)}| = \text{poly}(n)$. For all-to-all connectivity, we have

$$|\mathcal{P}_{\text{light}}^{(\leq k)}| = O(n_{\text{eff}}^k) = O(2^{mk}) \leq n^{O(\log n)}. \tag{164}$$

The time complexity is dominated by $|\mathcal{P}_{\text{light}}^{(\leq k)}|$, so we can conclude the proof. $\square$

IV. Quantum machine learning under non-unital noise: Barren plateaus

In this section, we rigorously show that non-unital noise induces absence of barren plateaus for local cost functions, in contrast to the unital scenario [50]. Specifically, in Subsection IV C we establish that the gates in the last $\Theta(\log(n))$ layers are trainable, whereas those preceding them are not. This complements the results presented in the previous section by rigorously showing the significance of the last $\Theta(\log(n))$ layers. Moreover, we establish that global cost functions exhibit barren plateaus. In Subsection IV D, we also present an improved upper bound on the onset of barren plateaus in the unital noise scenario compared to the one shown in Ref. [50].

The results we show in this section are in stark contrast with the behavior of quantum circuits in the noiseless regime or with local depolarizing noise [30, 73], as summarized in Table II.

Table II. **Trainability w.r.t. the last $g(n)$-layers**

**Noise model**	$g(n) = \omega(\log(n))$	$\Theta(\log(n))$	$\Theta(1)$
Noiseless [25, 30]	✗	✗	✗
Unital noise [50]	✗	✗	✗
Non-unital noise [This work]	✗	✓	✓

Table II shows that the last $\Theta(\log(n))$ layers of a non-unital noise circuit are the only trainable layers. This behavior is notably absent in the unital and noiseless noise regime for circuits with depth $\omega(\log(n))$: in these cases the gates in all the layers are not trainable.

A. Preliminaries on barren plateaus

1. *Cost functions*

In this section, we introduce concepts that will be crucial for our discussion. We use an analogous circuit model described in Subsection I D, namely we consider n-qubit quantum circuits Φ of the form

$$\Phi = (\mathcal{V}_L^{\text{single}} \circ \mathcal{N}^{\otimes n} \circ \mathcal{U}_L) \circ \cdots \circ (\mathcal{V}_1^{\text{single}} \circ \mathcal{N}^{\otimes n} \circ \mathcal{U}_1), \tag{165}$$

where L represents the number of layers, also referred to as circuit depth, $\{\mathcal{V}_k^{\text{single}}\}_{k=1}^{L}$ are layers of single-qubit gates distributed according a single-qubit 2-design, $\mathcal{U}_i := U_i(\cdot)U_i^{\dagger}$ corresponds to the n-qubit unitary channel associated with the unitary layer U_i for $i \in [L]$ which is formed by two-qubits gates, and $\mathcal{N}$ is a single-qubit quantum channel. Recall that we assume that the two-qubit gates in the circuit are distributed according to a two-qubit 2-design (see Definition 8). For example, our model encompasses the brickwork architecture in Fig. 1. Remember that, because of the unitary invariance of the two-qubit 2-design layers, one can add 'for free' layers of single-qubit Haar random gates, since we will be considering only up to second moment quantities. Thus, in the above equation, the layer of single-qubit gates (apart from the last one, $\mathcal{V}_L^{\text{single}}$) can be removed.

We now assume that the circuit is also also dependent on variational parameters $\boldsymbol{\theta} := (\theta_1, \dots, \theta_m) \in \mathbb{R}^m$, which parameterize some of the two-qubit gates, which come from the set $\{\exp(-i\theta_\mu H_\mu)\}_{\mu=1}^{m}$, where H_μ are two-local Hermitian operators with $\|H_\mu\|_\infty \leq 1$. Specifically, we assume for simplicity that these parameterized gates are positioned at the start of the unitary layer $\mathcal{U}_i$ for $i \in [L]$. It is important to note that while we introduce these parameterized gates, they do not impact our model due to left-right invariance of the two-qubit 2-design layers we consider, and so they can be considered part of one of the unitary layers $\mathcal{U}_i$ for $i \in [L]$; their introduction is primarily to facilitate the discussion on partial derivatives and barren plateaus.

In quantum machine learning jargon, the term *cost function* is usually referred to as an expectation value of an Hermitian operator over a 'parameterized' quantum state.

Definition 37 (Cost function)**.** *Let H be an Hermitian operator. Let ρ_0 be a quantum state and Φ be a noisy quantum circuit as defined previously. We define the cost function $C(\boldsymbol{\theta})$ associated with H and $\Phi(\rho_0)$ as*

$$C(\boldsymbol{\theta}) := \mathrm{Tr}(H\Phi(\rho_0)). \tag{166}$$

We will often omit the $\boldsymbol{\theta}$-dependence and write simply C instead of $C(\boldsymbol{\theta})$. As usual, when we write expected values or variances, it will always be with respect to the distribution from which we sample the gates that compose our quantum circuit.

Next, we introduce the notion of lack of barren plateaus.

Definition 38 (Lack of barren plateaus)**.** *We say a cost function C lacks barren plateaus if and only if*

$$\mathbb{E}\left[\|\nabla_{\boldsymbol{\theta}} C\|_2^2\right] = \Omega\left(\frac{1}{\mathrm{poly}(n)}\right), \tag{167}$$

where $\nabla_{\boldsymbol{\theta}} C \coloneqq (\frac{\partial C}{\partial \theta_1}, \cdots, \frac{\partial C}{\partial \theta_m})$ is the gradient of the cost function.

Hence, we assert that a cost function has barren plateaus if and only if the variance of the 2-norm of the gradient is at least super-polynomially small. We define now the notion of *trainability* of a parametrized gate, which is useful to identify which gate in the circuit influences significantly the cost function (on average).

Definition 39 (Trainability of the cost function with respect to a parameter)**.** *We say that a cost function C is trainable with respect the parameter θ_μ if and only if*

$$\mathrm{Var}[\partial_\mu C] = \Omega\left(\frac{1}{\mathrm{poly}(n)}\right), \tag{168}$$

where we have denoted $\partial_\mu C \coloneqq \frac{\partial C}{\partial \theta_\mu}$.

We point out that partial derivatives of expectation values are not only important for the consideration of barren plateaus, but also to understand which gate in the circuit has significant influence on the expectation value.

2. Review of previous results

In the noiseless scenario, initial observations by McClean *et al.* [25] pointed out that if the parameter distribution underlying the parametrized quantum circuit forms a global 2-design with respect to the Haar measure of n-qubit unitaries, then any associated cost function exhibits barren plateaus. Furthermore, when modeling a noiseless parametrized quantum circuit (often referred to as an *ansatz*) as a 'local random quantum circuit,' composed of geometrically local two-qubit gates, where each gate is distributed according to the Haar measure, barren plateaus start to manifest at $O(n)$ depth. This is because studies by Brandão *et al.* [27] have demonstrated that at linear $O(n)$ depth in one-dimensional architectures, the distribution over such circuits becomes 'approximately' a 2-design. Similar results have been extended to higher-dimensional quantum circuit architectures. Specifically, it has been shown by Harrow *et al.* [74] that the 'approximate' 2-design property emerges at $O(n^{1/\mathrm{D}})$, where D represents the dimension of the lattice of the circuit. Discussions concerning the relationship between barren plateaus and approximate notions of 2-design can be found in Ref. [26]. The influence of the locality of observables on the onset of barren plateaus has been explored in Ref. [28].

In one-dimensional architectures, it has been observed that while cost functions associated with $O(1)$-local observables do not exhibit barren plateaus at logarithmic depth, cost functions associated with global observables manifest barren plateaus even at constant depth. Furthermore, these results have been generalized in Ref. [30], where it has been noted that the gradient of the cost function decays exponentially with respect to the circuit depth and the Hamiltonian locality. These findings were established under the assumption that the 2-qubit gates composing the circuits are distributed according to a unitary 2-design. Methods for avoiding or mitigating barren plateaus in noiseless scenarios have been proposed, primarily relying on specific heuristic-based initialization strategies [26, 31–37], as well as by constraining the expressibility of the ansatz [38–44, 47, 48]. This constraint can be achieved, for instance, through the utilization of symmetries [43, 45]—from an intuitive perspective, these strategies aim to limit the expressiveness of the ansatz, rendering it less akin to a global 2-design with respect to the Haar measure over the full n-qubit unitary group. Furthermore, it has been argued/conjectured that if one can prove absence of barren plateaus, then one should also be able to classically simulate the ansatz class [49], either with purely classical resources or after an initial data acquisition phase, which may require a quantum computer.

In the context of noisy scenarios, an important observation has been pointed out in Ref. [50], revealing that both expectation values and gradients experience exponential decay in the circuit depth. Consequently, at linear depth, the expectation values and gradients of cost functions decay exponentially with respect to the number of qubits. This phenomenon has been dubbed 'noise-induced barren plateaus'. The results hold even without using randomness of the gates, i.e., for any fixed circuit. Significantly, this latter study assumed the presence of a local depolarizing noise model, which is unital in nature. Strikingly, even when employing error mitigation strategies, it appears challenging to effectively counteract the emergence of noise-induced barren plateaus, as argued in Ref. [75].

A concurrent and independent work [59] has studied the impact of noise beyond unital on the barren plateaus phenomenon. However, this study is restricted to the so-called HS-contractive noise, whereas, to the best of our knowledge, our work is the first to rigorously address a general kind of possibly non-unital local noise.

As we will prove, the last $O(\log(n))$ layers of the circuit are trainable, meaning they do not suffer from (sub-)exponentially vanishing partial derivatives. This results in the norm of the gradient of the cost function being sufficiently large. However, we will also show that the partial derivatives taken before these logarithmic many layers in the circuit are negligible, which is why we refer to this as an 'effective log-depth circuit'.

B. Gradients: useful lemmas

We now give a formula to compute directly the partial derivative, which can be useful to handle calculations. However, one might also take it as an equivalent definition of partial derivative.

Lemma 40 (Partial derivative)**.** *Let $\mu \in [m]$. Consider a parameterized 2-qubit gate $\exp(-i\theta_\mu H_\mu)$, positioned at the start of unitary layer $\mathcal{U}_k$, where $k \in [L]$ is the index of the layer where the gate is positioned in the circuit. We have*

$$\partial_\mu C = i\,\mathrm{Tr}\Big(\Phi_{[1,k-1]}(\rho_0)\,\Big[H_\mu, \Phi^*_{[k,L]}(H)\Big]\Big)\,, \tag{169}$$

where we have denoted

$$\Phi_{[a,b]} := (\mathcal{V}_b^{\text{single}} \circ \mathcal{N}^{\otimes n} \circ \mathcal{U}_b) \circ \cdots \circ (\mathcal{V}_a^{\text{single}} \circ \mathcal{N}^{\otimes n} \circ \mathcal{U}_a), \tag{170}$$

for $a \le b \in [L]$.

Proof. We can write the cost function as

$$C = \mathrm{Tr}(\Phi(\rho_0)H) = \mathrm{Tr}\big(\Phi_{[1,k]} \circ \Phi_{[k,L]}(\rho_0)H\big) = \mathrm{Tr}\Big(\Phi_{[k,L]}(\rho_0)\Phi^*_{[1,k]}(H)\Big)\,. \tag{171}$$

By taking the partial derivative with respect the parameter θ_μ, we have

$$\begin{aligned}\partial_\mu C &= \mathrm{Tr}\Big(\Phi_{[1,k]}(\rho_0)\partial_\mu(\Phi^*_{[k,L]}(H))\Big) \\ &= i\,\mathrm{Tr}\Big(\Phi_{[1,k]}(\rho_0)H_\mu\Phi^*_{[k,L]}(H)\Big) - i\,\mathrm{Tr}\Big(\Phi_{[1,k]}(\rho_0)\Phi^*_{[k,L]}(H)H_\mu\Big) \\ &= i\,\mathrm{Tr}\Big(\Phi_{[1,k]}(\rho_0)\,\Big[H_\mu, \Phi^*_{[k,L]}(H)\Big]\Big)\,,\end{aligned} \tag{172}$$

where we have used the fact that $\partial_\mu \exp(-i\theta_\mu H_\mu) = -iH_\mu \exp(-i\theta_\mu H_\mu)$. ☐

From now on, when using the above formula for the partial derivatives, since we are considering second moment quantities, we will ignore the parametrized gates in the circuit, since they can be absorbed in the two-qubit 2-design layers. We now show that the expected value of the partial derivative with respect any parameter is zero.

Lemma 41 (Expected value of the partial derivative of the cost function)**.** *The expected value of the partial derivative of the cost function is* 0 *with respect any parameter, i.e.,*

$$\mathbb{E}[\partial_\mu C] = 0. \tag{173}$$

Proof. Due to left and right invariance, the 2-qubit parameterized unitaries can be absorbed in the 2-design unitaries. Moreover, by Eq. (169), we have

$$\partial_\mu C = i\,\mathrm{Tr}\Big(\Phi_{[1,k]}(\rho_0)\,\Big[H_\mu, \Phi^*_{[k,L]}(H)\Big]\Big)\,. \tag{174}$$

Since $\Phi^*_{[k,L]}(H)$ ends with a layer of single-qubits 2-design gates and these form a 1-design (Lemma 4) by taking the expected value only over that layer, we have

$$\begin{aligned}\mathbb{E}\left[\partial_\mu C\right] &= i\,\mathrm{Tr}\Big(\Phi_{[k,L]}(\rho_0)\,\Big[H_\mu, \mathbb{E}\left(\Phi^*_{[k,L]}(H)\right)\Big]\Big) \\ &= i\,\mathrm{Tr}\left(\Phi_{[k,L]}(\rho_0)\left[H_\mu, \mathrm{Tr}\Big(\Phi^*_{[k,L]}(H)\Big)\frac{I_n}{2^n}\right]\right) \\ &= 0,\end{aligned} \tag{175}$$

where we have used the first moment formula (Eq. (12)) and the fact that any operator commutes with the identity. ☐

The previous Lemma implies that $\mathrm{Var}[\partial_\mu C] = \mathbb{E}[(\partial_\mu C)^2]$, so we care only about the latter quantity from now on. We now present a lemma, similar in spirit to Lemma 5, which will be useful to deal with upper and lower bounds of partial derivatives.

Lemma 42 (Pauli mixing to gradients)**.** *Let H_μ be a 2-local Hamiltonian. Let $f(\cdot) := i\,\mathrm{Tr}\Big(\Phi_{[1,k]}(\rho_0)\,\Big[H_\mu, \Phi^*_{[k,L]}(\cdot)\Big]\Big)$ be an operator function. Then we have*

- *Let* $H \coloneqq \sum_{P\in\{I,X,Y,Z\}^{\otimes n}} a_P P$*, with* $a_P \in \mathbb{R}$ *for any* $P \in \{I,X,Y,Z\}^{\otimes n}$*. We have*

$$\mathbb{E}[(f(H))^2] = \sum_{P\in\{I,X,Y,Z\}^{\otimes n}} a_P^2 \mathbb{E}[(f(P))^2], \tag{176}$$

- *Moreover, for any* $P \in \{I,X,Y,Z\}^{\otimes n}$*, we have*

$$\mathbb{E}[(f(P))^2] = \frac{1}{3^{|P|}} \sum_{\substack{Q\in\{I,X,Y,Z\}^{\otimes n}:\\ \mathrm{supp}(Q)=\mathrm{supp}(P)}} \mathbb{E}[(f(Q))^2]. \tag{177}$$

Proof. We have

$$\begin{aligned}
(f(H))^2 &= \left(i\,\mathrm{Tr}\left(\Phi_{[1,k]}(\rho_0)\left[H_\mu, \Phi^*_{[k,L]}(H)\right]\right)\right)^2 \\
&= \left(\sum_{P\in\{I,X,Y,Z\}^{\otimes n}} a_P\,\mathrm{Tr}\left(\Phi_{[1,k]}(\rho_0)\left[\Phi^*_{[k,L]}(P), H_\mu\right]\right)\right)^2 \\
&= \sum_{P,Q\in\{I,X,Y,Z\}^{\otimes n}} a_P a_Q\,\mathrm{Tr}\left(\Phi_{[1,k]}(\rho_0)\left[\Phi^*_{[k,L]}(P), H_\mu\right]\right)\mathrm{Tr}\left(\Phi_{[1,k]}(\rho_0)\left[\Phi^*_{[k,L]}(Q), H_\mu\right]\right) \\
&= \sum_{P,Q\in\{I,X,Y,Z\}^{\otimes n}} a_P a_Q\,\mathrm{Tr}\left(\Phi_{[1,k]}(\rho_0)^{\otimes 2}\left(\left[\Phi^*_{[k,L]}(P), H_\mu\right]\otimes\left[\Phi^*_{[k,L]}(Q), H_\mu\right]\right)\right).
\end{aligned} \tag{178}$$

We now consider the expected value of this quantity with respect the final unitary layer in $\Phi^*_{[k,L]}$ (specifically, the layer that acts directly on P and Q). Such expected value reduces to computing the expected value

$$\mathbb{E}\left(\left[\Phi^*_{[k,L]}(P), H_\mu\right]\otimes\left[\Phi^*_{[k,L]}(Q), H_\mu\right]\right). \tag{179}$$

By expanding the two commutators, we have

$$\begin{aligned}
&\left[\Phi^*_{[k,L]}(P), H_\mu\right]\otimes\left[\Phi^*_{[k,L]}(Q), H_\mu\right] \\
&= (\Phi^*_{[k,L]}(P)\otimes\Phi^*_{[k,L]}(Q))(H_\mu\otimes H_\mu) - (I_n\otimes H_\mu)(\Phi^*_{[k,L]}(P)\otimes\Phi^*_{[k,L]}(Q))(H_\mu\otimes I_n) \\
&\quad - (H_\mu\otimes I_n)(\Phi^*_{[k,L]}(P)\otimes\Phi^*_{[k,L]}(Q))(I_n\otimes H_\mu) + (H_\mu\otimes H_\mu)(\Phi^*_{[k,L]}(P)\otimes\Phi^*_{[k,L]}(Q)).
\end{aligned} \tag{180}$$

Consequently, our attention can be directed solely towards the expression

$$\begin{aligned}
\mathbb{E}(\Phi^*_{[k,L]}(P)\otimes\Phi^*_{[k,L]}(Q)) &= \mathbb{E}\left(\Phi^{*\otimes 2}_{[k,L]}\left(\mathbb{E}_{\mathcal{V}}(\mathcal{V}^{\mathrm{single}}(P)\otimes\mathcal{V}^{\mathrm{single}}(Q))\right)\right) \\
&= \delta_{P,Q}\mathbb{E}\left(\Phi^{*\otimes 2}_{[k,L]}\left(\mathbb{E}_{\mathcal{V}}(\mathcal{V}^{\mathrm{single}}(P)\otimes\mathcal{V}^{\mathrm{single}}(P))\right)\right) \\
&= \delta_{P,Q}\frac{1}{3^{|P|}}\sum_{\substack{R\in\{I,X,Y,Z\}^{\otimes n}:\\ \mathrm{supp}(R)=\mathrm{supp}(P)}}\mathbb{E}\left(\Phi^{*\otimes 2}_{[k,L]}(R\otimes R)\right) \\
&= \delta_{P,Q}\frac{1}{3^{|P|}}\sum_{\substack{R\in\{I,X,Y,Z\}^{\otimes n}:\\ \mathrm{supp}(R)=\mathrm{supp}(P)}}\mathbb{E}\left(\Phi^*_{[k,L]}(R)\otimes\Phi^*_{[k,L]}(R)\right),
\end{aligned} \tag{181}$$

where in the first step we singled out 'for free' a layer of Haar random gates from $\Phi^*_{[k,L]}$, in the second step we applied the Pauli mixing formula Eq. (17) for each of the single qubits gates (similarly as done in Lemma 5). Therefore, by substituting in Eq. (180) and repeating the steps backwards, we can conclude. □

Because of the previous lemma, computing the variance of a cost function partial derivative defined with respect an Hermitian operator reduces to computing the variance of a cost function partial derivative defined with respect a Pauli operator. Consequently, we have the following corollary.

Corollary 43 (Partial derivative variance of an Hamiltonian)**.** *Let $\mu \in [m]$ be the index of the parameter θ_μ which parametrize a gate in the k-th layer. Let $H := \sum_{P\in\{I,X,Y,Z\}^{\otimes n}} a_P P$, with $a_P \in \mathbb{R}$ for any $P \in \{I,X,Y,Z\}^{\otimes n}$. We have*

$$\mathrm{Var}[\partial_\mu C] = \sum_{P\in\{I,X,Y,Z\}^{\otimes n}} a_P^2 \mathrm{Var}[\partial_\mu C_P], \tag{182}$$

where $C_P := \mathrm{Tr}(P\Phi(\rho_0))$ with Φ and ρ_0 are respectively the noisy quantum circuit and the initial state.

Proof. This follows immediately from Lemma 41 and Lemma 42. □

We now show a worst-case upper bound on the α-th order partial derivative that will be useful later.

Lemma 44 (α-th order partial derivative upper bound)**.** *The α-th order partial derivative with respect the parameter θ_μ is upper bounded by*

$$\left|\partial_\mu^\alpha C\right| \leq 2^\alpha \|H\|_\infty \|H_\mu\|_\infty^\alpha \tag{183}$$

Proof. We have $C = \mathrm{Tr}(\Phi(\rho_0)H) = \mathrm{Tr}\left(\Phi_{[1,k]}(\rho_0)\Phi^*_{[k,L]}(H)\right)$. Thus

$$\partial_\mu^\alpha C = \mathrm{Tr}\left(\Phi_{[1,k]}(\rho_0)\,\partial_\mu^\alpha \Phi^*_{[k,L]}(H)\right). \tag{184}$$

Because of Hölder inequality, we have $|\partial_\mu^\alpha C| \leq \|\partial_\mu^\alpha \Phi^*_{[k,L]}(H)\|_\infty$, where we also used that the one-norm of a quantum state is one. We now prove by induction that

$$\|\partial_\mu^\alpha \Phi^*_{[k,L]}(H)\|_\infty \leq 2^\alpha \|H\|_\infty \|H_\mu\|_\infty^\alpha. \tag{185}$$

For $\alpha = 1$, we have

$$\partial_\mu \Phi^*_{[k,L]}(H) = i\left[H_\mu, \Phi^*_{[k,L]}(H)\right], \tag{186}$$

where we have used the fact that $\partial_\mu \exp(-i\theta_\mu H_\mu) = -iH_\mu \exp(-i\theta_\mu H_\mu)$ as done in the proof of Lemma 40. Thus,

$$\|\partial_\mu \Phi^*_{[k,L]}(H)\|_\infty \leq 2\|H_\mu\|_\infty \|\Phi^*_{[k,L]}(H)\|_\infty \leq 2\|H_\mu\|_\infty \|H\|_\infty, \tag{187}$$

where we have used the triangle inequality, submultiplicativity of the p-norms and in the last step we have used the inequality $\|\Phi^*(O)\|_\infty \leq \|O\|_\infty$ (see, e.g., Ref. [7]) valid for all operators O. This shows the base case. For $\alpha > 1$, we have

$$\|\partial_\mu^\alpha \Phi^*_{[k,L]}(H)\|_\infty = \|\partial_\mu^{\alpha-1} i\left[H_\mu, \Phi^*_{[k,L]}(H)\right]\|_\infty = \|\left[H_\mu, \partial_\mu^{\alpha-1}\Phi^*_{[k,L]}(H)\right]\|_\infty \leq 2\|H_\mu\|_\infty \|\partial_\mu^{\alpha-1}\Phi^*_{[k,L]}(H)\|_\infty, \tag{188}$$

where in the last step we have used triangle inequality and submultiplicativity. We can conclude by the using the induction step. □

We introduce a precise definition of the standard notion of the light-cone of an observable with respect to a quantum channel (typically representing a quantum circuit).

Definition 45 (Light cone)**.** *Let H be an Hermitian operator and Φ be a quantum channel. The light-cone of H with respect to Φ is defined as*

$$\mathrm{Light}(\Phi, H) := \mathrm{supp}(\Phi^*(H)), \tag{189}$$

where $\mathrm{supp}(\cdot)$ is defined in our notation section.

In the subsequent subsection, we need to consider the light cone not with respect to only a specific quantum circuit, but with respect to a family of quantum circuits provided by the support of a considered random quantum circuits probability distribution, denoted as $\mathcal{F}$ (recall that the support of a random variable is defined as the set of all values for which the probability density function is strictly greater than zero). Formally, we define:

$$\mathrm{Light}_{\mathcal{F}}(H) := \bigcup_{\Phi\in\mathcal{F}} \mathrm{supp}(\Phi^*(H)). \tag{190}$$

In particular, we consider the family $\mathcal{F}_k$ of quantum circuits corresponding to the support of the probability distribution associated with $\Phi_{[k,L]}$, where $k \in [L]$. To streamline the notation, we refer to $\mathrm{Light}_{\mathcal{F}_k}(H)$ as the 'light-cone of H with respect to $\Phi_{[k,L]}$'. In Fig. 3, we provide a graphical example for a one-dimensional geometrical local quantum circuit. We now give the following Lemma, which will be useful later on.

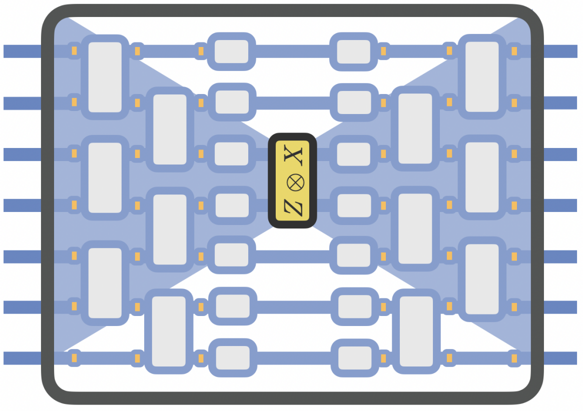

Figure 3. The light-cone of a local Pauli observable with respect to $\Phi_{[1,L]}$ is the set of qubits within the shaded area. The (noisy) gates outside the blue shaded area are contracted trivially due to the fact that any adjoint channel is unital, and thus cannot influence the expectation value of the Pauli.

Lemma 46 (Partial derivative is zero outside the light cone)**.** *Let H be an Hermitian operator. Let $\mu \in [m]$. Consider a parameterized 2-qubit gate $\exp(-i\theta_\mu H_\mu)$, positioned in the k-th layer, such that its Hamiltonian generator H_μ has support outside the light cone of $\Phi_{[k,L]}$ with respect to H. Then, the partial derivative is zero $\partial_\mu C = 0$..*

Proof. In words, the partial derivative must be zero since the cost function does not depend effectively by the gates outside the light-cone (because they contract trivially). However, formally this can be seen as follows. By using Eq. (169), we have

$$\partial_\mu C = i\,\mathrm{Tr}\Big(\Phi_{[1,k]}(\rho_0)\left[H_\mu, \Phi^*_{[k,L]}(H)\right]\Big)\,. \tag{191}$$

Since H_μ has support outside the light cone of $\Phi_{[k,L]}$ with respect to H, by definition of light-cone it follows that $\left[H_\mu, \Phi^*_{[k,L]}(H)\right] = 0$. □

C. Absence of barren plateaus, but only few layers are trainable

In this subsection, we will show that the gates in the last $\Theta(\log(n))$ layers (in the light cone of a local observable) are the only trainable gates of the circuits. This also implies that the expectation value of an observable can be significantly influenced only by such gates in the last layers. We first state our main claims here, which will then be detailed further. Leveraging Corollary 43, we focus on cost functions associated with Pauli observables instead of general Hermitian operator, without loss of generality. In Subsection IV C 1, we present the following upper bound assuming constant noise parameters:

Theorem 47 (Layers before $\Theta(\log(n))$ layers are not trainable)**.** *Let $C := \mathrm{Tr}(P\Phi(\rho_0))$ be the cost function, where $P \in \{I, X, Y, Z\}^{\otimes n}$, ρ_0 is an arbitrary initial state, and Φ is a noisy quantum circuit of depth L. We assume that the noise is not a unitary channel. Let μ denote a parameter of a gate in the k-th layer of the circuit. Then, we have*

$$\mathrm{Var}[\partial_\mu C] \leq \exp(-\Omega(|P| + L - k)). \tag{192}$$

This result immediately implies that the gates before the last $\Theta(\log(n))$ layers are not trainable. Moreover, it directly implies barren plateaus for global cost functions.

Corollary 48 (Global cost function induced barren plateaus)**.** *Let C be the cost function associated with a Pauli P with $|P| = \Theta(n)$. Then, we have $\mathrm{Var}[\partial_\mu C] \leq \exp(-\Omega(n))$.*

In subsubsection IV C 1, we establish the following lower bound assuming constant noise parameters.

Theorem 49 (Last $\Theta(\log(n))$ layers do matter)**.** *Let $C := \mathrm{Tr}(P\Phi(\rho_0))$ be the cost function, where $P \in \{I, X, Y, Z\}^{\otimes n}$, ρ_0 is an arbitrary state, and Φ is a non-unital noisy quantum circuit of depth L . We assume noise is not a replacer channel. Let μ denote a parameter of a gate in the k-th layer of the circuit. Then, if the support of such a gate is contained in the light cone of $\Phi_{[k,L]}$ with respect to the Pauli P, we have*

$$\mathrm{Var}[\partial_\mu C] \geq \exp(-O(|P|(L-k)))\,, \tag{193}$$

otherwise, if the support of the parametrized gate is outside the light cone, we have $\mathrm{Var}[\partial_\mu C] = 0$.

Note that the variance upper and lower bounds are matched for local cost functions (i.e., $|P| = O(1)$). Moreover, the latter theorem leads to the following corollary affirming the absence of barren plateaus for local cost functions:

Corollary 50 (Absence of barren plateaus for local cost functions)**.** *Let C be a cost function associated with a local Pauli P. We assume non-unital noise and that is not a replacer channel. Then, we have*

$$\mathbb{E}\left[\|\nabla C\|_2^2\right] \geq \Omega(1). \tag{194}$$

However, this absence of barren plateaus in arbitrarily deep quantum circuit is only due to the last $\Theta(\log(n))$ layers which significantly influence the expectation value of local observables. Furthermore, in Subsection IV D, we show improved upper bound for the onset of barren plateaus in the unital noise scenario, improving upon previous works [50].

1. Partial derivative upper bound: Layers before the last $O(\log(n))$ are not trainable

We are now going to show the upper bound on the partial derivative. Here, we do not make any assumption on the geometrical locality of the circuit.

Proposition 51 (Partial derivative upper bound)**.** *Let $C := \mathrm{Tr}(P\Phi(\rho_0))$ be the cost function, where $P \in \{I, X, Y, Z\}^{\otimes n}$, ρ_0 is an arbitrary initial state, and Φ is a noisy quantum circuit of depth L. We assume that the noise is not a unitary channel. Let μ denote a parameter of a gate in the k-th layer of the circuit. Then, we have*

$$\mathrm{Var}[\partial_\mu C] \leq 4c^{(|P|+L-k-1)}. \tag{195}$$

Proof. **Proof method 1:** We show first a shorter and more immediate proof method, which yields a slightly worse upper bound $O(c^{(|P|+L-k-1)/3})$ albeit always with the desired exponential scaling. Let $K := L - k$. We have $\mathrm{Var}[\partial_\mu C] = \mathbb{E}[(\partial_\mu C)^2]$ due to Eq. (183). Due to the Taylor remainder theorem and for any three differentiable functions $f(x)$, this relationship can be expressed using standard finite-difference formulas (see, e.g., here):

$$|\partial f(x)| \leq \left|\frac{f(x+h) - f(x-h)}{2h}\right| + \frac{h^2}{6}|\sup(\partial^3 f)|, \tag{196}$$

for any $h \in [0, \infty)$. Hence

$$\begin{aligned}(\partial f(x))^2 &\leq \left|\frac{f(x+h) - f(x-h)}{2h}\right|^2 + \frac{h^4}{36}|\sup(\partial^3 f)|^2 + 2\left|\frac{f(x+h) - f(x-h)}{2h}\right|\frac{h^2}{3}|\sup(\partial^3 f)| \\ &\leq \left|\frac{f(x+h) - f(x-h)}{2h}\right|^2 + \frac{h^4}{36}|\sup(\partial^3 f)|^2 + \frac{h}{3}\,|\sup(f)|\,|\sup(\partial^3 f)|.\end{aligned} \tag{197}$$

By using this for our function C with respect the parameter θ_μ, taking the expected values both terms and using Lemma 44, we have

$$\begin{aligned}\mathbb{E}(\partial_\mu C)^2 &\leq \mathbb{E}\left|\frac{\mathrm{Tr}\big(P\Phi_{[L-K+1,L]}(\rho)\big) - \mathrm{Tr}\big(P\Phi_{[L-K+1,L]}(\sigma)\big)}{2h}\right|^2 + 2^6\frac{h^4}{36}\|H_\mu\|_\infty^6 + 2^3\frac{h}{3}\|H_\mu\|_\infty^3 \\ &\leq \frac{1}{4h^2}c^{K+|P|-1} + 4h^4\|H_\mu\|_\infty^6 + 4h\|H_\mu\|_\infty^3 \\ &\leq \frac{1}{4}c^{(K+|P|-1)/3} + 8c^{(K+|P|-1)/3} \\ &\leq 9c^{(K+|P|-1)/3},\end{aligned} \tag{198}$$

where we have defined ρ and σ to be defined as the state $\mathrm{Tr}\big(P\Phi_{[1,L-K]}(\rho_0)\big)$ computed respectively respectively in $\theta_\mu + h$ and $\theta_\mu - h$, and we have chosen $h := c^{(K+|P|)/3}$.

Proof method 2: We now establish a tighter upper bound using the partial derivative formula involving the commutator (Eq. (169)). The proof follows a similar spirit to the one used in Proposition 16 (i.e., effective depth). Instead of using Lemma 5 (as in the effective depth proof), we employ the analogous Lemma 42. First, we have

$$\mathrm{Var}[\partial_\mu C] = \mathbb{E}[(\partial_\mu C)^2] = \mathbb{E}[f_0(P)^2], \tag{199}$$

where we define the function

$$f_j(\cdot) := i\,\mathrm{Tr}\Big(\Phi_{[1,k]}(\rho_0)\,\Big[H_\mu, \Phi^*_{[k,L-j]}(\cdot)\Big]\Big)\,. \tag{200}$$

Using Lemma 42 and averaging over the last layer of single qubit gates in $\Phi^*_{[k,L]}$, we have

$$\mathbb{E}[(f_0(P)^2] = \frac{1}{3^{|P|}} \sum_{\substack{Q\in\{I,X,Y,Z\}^{\otimes n}:\\ \mathrm{supp}(Q)=\mathrm{supp}(P)}} \mathbb{E}[(f_0(Q))^2]. \tag{201}$$

Let us focus on $\mathbb{E}[(f_0(Q))^2]$. Taking the adjoint of the last layer of noise on Q, we get

$$\mathcal{N}^{*\otimes n}(Q) = \bigotimes_{j\in\mathrm{supp}(Q)} (t_{Q_j} I_j + D_{Q_j} Q_j) = \sum_{a\in\{0,1\}^{|Q|}} \bigotimes_{j\in\mathrm{supp}(Q)} (t_{Q_j}^{a_j} D_{Q_j}^{1-a_j} Q_j^{1-a_j}). \tag{202}$$

We define the function $f'_j(\cdot)$ as

$$f'_j(\cdot) := i\,\mathrm{Tr}\Big(\Phi_{[1,k]}(\rho_0)\,\Big[H_\mu, \Phi'^*_{[k,L-j]}(\cdot)\Big]\Big)\,, \tag{203}$$

where $\Phi'_{[k,L-j]}$ is equal to $\Phi_{[k,L-j]}$ but without the last layer of single qubit gates and noise. Applying Lemma 42 again, we have

$$\begin{aligned}\mathbb{E}[(f_0(Q))^2] &= \sum_{a\in\{0,1\}^{|Q|}} \prod_{j\in\mathrm{supp}(Q)} t_{Q_j}^{2a_j} D_{Q_j}^{2(1-a_j)} \mathbb{E}[(f'_0(\bigotimes_{j\in\mathrm{supp}(Q)} Q_j^{1-a_j}))^2] \\ &\le \sum_{a\in\{0,1\}^{|Q|}} \prod_{j\in\mathrm{supp}(Q)} t_{Q_j}^{2a_j} D_{Q_j}^{2(1-a_j)} \max_{R\in\{I,X,Y,Z\}^{\otimes n}} \mathbb{E}[(f'_0(R))^2].\end{aligned} \tag{204}$$

Substituting, we arrive at

$$\begin{aligned}\mathbb{E}[(f_0(P)^2] &= \frac{1}{3^{|P|}} \sum_{\substack{Q\in\{I,X,Y,Z\}^{\otimes n}:\\ \mathrm{supp}(Q)=\mathrm{supp}(P)}} \mathbb{E}[(f_0(Q))^2] \\ &\le \frac{1}{3^{|P|}} \sum_{\substack{Q\in\{I,X,Y,Z\}^{\otimes n}:\\ \mathrm{supp}(Q)=\mathrm{supp}(P)}} \sum_{a\in\{0,1\}^{|Q|}} \prod_{j\in\mathrm{supp}(Q)} t_{Q_j}^{2a_j} D_{Q_j}^{2(1-a_j)} \max_{R\in\{I,X,Y,Z\}^{\otimes n}} \mathbb{E}[(f'_0(R))^2] \\ &= c^{|P|} \max_{R\in\{I,X,Y,Z\}^{\otimes n}} \mathbb{E}[(f'_0(R))^2],\end{aligned} \tag{205}$$

where we have used the multinomial theorem together with the fact that

$$c = \frac{1}{3^{|P|}}(\|\mathbf{D}\|_2^2 + \|\mathbf{t}\|_2^2). \tag{206}$$

As in the proof of Proposition 16, we can assume that the maximum over Pauli in the latter equation is achieved by a Pauli different from the identity (otherwise the RHS would be zero). Moreover, we can assume now that all the two-qubit gates in the circuit are Clifford, as we are computing a second moment, and the Cliffords form a 2-design. Thus, the two qubit gates of the circuit will also map Pauli to Pauli. Therefore, the Pauli above will be mapped by the two-qubits Clifford to another Pauli still different from the identity. Since now we have a circuit that ends with a layer of single qubits 2-design unitaries, which are preceded by a noise layer and a layer of two-qubits 2-design gates, we are in the same situation we faced at the beginning of the proof with

$$\mathbb{E}[(f_0(P)^2] \le c^{|P|} \max_{Q\in\{I,X,Y,Z\}^{\otimes n}\setminus I_n} \mathbb{E}[(f_1(Q)^2]. \tag{207}$$

So reiterating the argument to the next layers, and using that the Pauli weight of the considered Pauli at each iteration is at least one, we have

$$\mathbb{E}[(f_0(P)^2] \le c^{|P|+L-k-1} \max_{Q\in\{I,X,Y,Z\}^{\otimes n}\setminus I_n} \mathbb{E}[(f_{L-k}(Q)^2]. \tag{208}$$

By using the definition of $f_{L-k}(\cdot)$, we have

$$\begin{aligned}\mathbb{E}[(f_0(P)^2] &\leq c^{|P|+L-k-1}\mathbb{E}\max_{Q\in\{I,X,Y,Z\}^{\otimes n}\setminus I_n}\left(i\operatorname{Tr}\left(\Phi_{[1,k]}(\rho_0)\,[H_\mu,Q]\right)\right)^2 \\ &\leq c^{|P|+L-k-1}\max_{Q\in\{I,X,Y,Z\}^{\otimes n}\setminus I_n}\mathbb{E}\|\Phi_{[1,k]}(\rho_0)\|_1^2\|[H_\mu,Q]\|_\infty^2 \\ &\leq 4c^{|P|+L-k-1}\max_{Q\in\{I,X,Y,Z\}^{\otimes n}\setminus I_n}\|H_\mu\|_\infty^2\|Q\|_\infty^2 \\ &\leq 4c^{|P|+L-k-1},\end{aligned} \tag{209}$$

where we have used the Hölder inequality in the second step, submultiplicativity of the infinity norm in the third step, and in the last step the fact that all the involved norms are ≤ 1.

□

We point out that the previous statement could also have been proved using the Parameter Shift Rule [76] assuming a restricted class of parameterized gates, that is, of the form $\exp(i\theta_\mu H_\mu)$, with H_μ such that $H_\mu^2 = I$. But for the sake of generality we decided to use the proof methods presented. In this connection, note that the parameter shift rule also applies to noisy circuits, as can be seen, e.g., by making use standard Stinespring dilation arguments.

We point out that by applying Chebyshev's inequality, the upper bound on the variance can be translated into the probability statement as

$$\operatorname{Prob}\left(|\partial_\mu C| > \varepsilon\right) \leq \frac{\operatorname{Var}[\partial_\mu C]}{\varepsilon^2} \leq \frac{4}{\varepsilon^2}c^{|P|+L-k-1}. \tag{210}$$

This equation implies that the probability of sampling a point in the parameters space such that the absolute value of the partial derivative is greater than ε decays exponentially with both the Pauli weight $|P|$ and $L-k$, which is the distance from the end of the circuit to the layer where the partial derivative is taken.

The previous proposition also directly implies that cost functions associated with global Pauli operators (i.e., $\Theta(n)$ Pauli weight) have all partial derivatives exponentially vanishing in the number of qubits.

Corollary 52 (Global cost function induced barren plateaus)**.** *Let C be a cost function associated with a global Pauli operator, i.e., with $|P| = \Theta(n)$. Let μ denote a parameter of a gate in any of the layers. Then, we have*

$$\operatorname{Var}[\partial_\mu C] \leq \exp(-\Omega(n)). \tag{211}$$

2. *Partial derivative lower bound: the last $\Theta(\log(n))$ layers are the only trainable*

Here, we establish a lower bound on the partial derivative variance, valid any fixed circuit architecture (e.g., in constant dimension or all-to-all connectivity). The proof technique employed here is novel and may be of independent interest, allowing lower bounds on other second or third moment quantities of noisy random quantum circuits. In summary, dealing with a second-moment quantity and considering all 2-qubit gates in the circuit as local 2-designs (effectively Clifford gates), we condition on specific *Clifford choices* among various combinations to obtain a non-trivial lower bound.

Here, we assume that the noise is non-unital (i.e., $\|\mathbf{t}\|_2 = \Theta(1)$) and that the noise is not a replacer channel (i.e., the noise parameter $\|\mathbf{D}\|_2$ is a non-zero constant).

Proposition 53 (Partial derivative lower bound)**.** *Let $C := \operatorname{Tr}(P\Phi(\rho_0))$ be the cost function, where $P \in \{I, X, Y, Z\}^{\otimes n}$, ρ_0 is an arbitrary initial state, and Φ is a non-unital noisy quantum circuit of depth L. We also assume that the noise is not a replacer channel (otherwise, any partial derivative would be zero). Let μ denote the parameter θ_μ of the gate $\exp(-i\theta_\mu H_\mu)$ in the k-th layer of the circuit. Then, if the support of such a gate is contained in the light cone of $\Phi_{[k,L]}$ with respect to the Pauli P, we have*

$$\operatorname{Var}[\partial_\mu C] \geq \exp(-\Theta(|P|(L-k)))\,, \tag{212}$$

otherwise, if the support of the parametrized gate is outside the light cone, we have $\operatorname{Var}[\partial_\mu C] = 0$.

Proof. If the support of the parametrized gate is outside the light cone, $\partial_\mu C = 0$, as stated in Lemma 46. Therefore, we focus on the case in which the gate is within the light cone. By employing Lemma 41 and 40 to express the variance of the partial derivative, we have

$$\operatorname{Var}[\partial_\mu C] = \mathbb{E}[(\partial_\mu C)^2] = \mathbb{E}[f_0(P)^2]. \tag{213}$$

Here, $f_j(\cdot) := i\,\mathrm{Tr}\Big(\Phi_{[1,k]}(\rho_0)\left[H_\mu, \Phi^*_{[k,L-j]}(\cdot)\right]\Big)$. By applying Lemma 42 and averaging over the last layer of single-qubit gates in $\Phi^*_{[k,L]}$, we arrive at

$$\mathrm{Var}[\partial_\mu C] = \frac{1}{3^{|P|}} \sum_{\substack{R\in\{I,X,Y,Z\}^{\otimes n}:\\ \mathrm{supp}(R)=\mathrm{supp}(P)}} \mathbb{E}[(f_0(R))^2] \geq \frac{1}{3^{|P|}}\left(\mathbb{E}[(f_0(P_X))^2] + \mathbb{E}[(f_0(P_Y))^2] + \mathbb{E}[(f_0(P_Z))^2]\right), \tag{214}$$

where $P_X := \bigotimes_{j\in\mathrm{supp}(P)} X_j$, and P_Y, P_Z are similarly defined. Focusing on $\mathbb{E}[(f_0(P_X))^2]$, we define the function $f'_j(\cdot)$ as

$$f'_j(\cdot) := i\,\mathrm{Tr}\Big(\Phi_{[1,k]}(\rho_0)\left[H_\mu, \Phi'^*_{[k,L-j]}(\cdot)\right]\Big), \tag{215}$$

with $\Phi'_{[k,L-j]}$ identical to $\Phi_{[k,L-j]}$ but lacking the last layer of single-qubit gates and noise. Taking the adjoint of the last layer of noise on P_X and applying Lemma 42 again, we obtain

$$\begin{aligned}\mathbb{E}[(f_0(P_X))^2] &= \sum_{a\in\{0,1\}^{|P_X|}} \prod_{j\in\mathrm{supp}(P_X)} t^{2a_j}_{(P_X)_j} D^{2(1-a_j)}_{(P_X)_j} \mathbb{E}[(f'_0(\bigotimes_{j\in\mathrm{supp}(P_X)} (P_X)_j^{1-a_j}))^2] \\ &\geq D_X^{2|P|}\mathbb{E}[(f'_0(P_X)^2].\end{aligned} \tag{216}$$

Now, we delve into the technical part of this proof. As is customary when dealing with second-moment quantities, we treat our circuits as random Clifford circuits. The ultimate goal is to ensure that the commutator $\left[H_\mu, \Phi^*_{[k,L-j]}(\cdot)\right]$ is non-zero for some Clifford gate instances. To achieve this, we fix *some* of the 2-qubit Clifford gates in the circuit. However, caution is required not to fix all the Clifford gates, as this would result in unfavorable scaling. Specifically, each 2-qubit Clifford C_{fixed} that we fixed in the circuit contributes a factor of $|\mathrm{C}_2|^{-1}$, where $|\mathrm{C}_2|$ is the size of the 2-qubit Clifford group C_2. This is expressed by the lower bound

$$\mathbb{E}_{C\sim\mathrm{C}_2}[g(C)] = \frac{1}{|\mathrm{C}_2|}\sum_{C\in\mathrm{C}_2}(g(C))^2 \geq \frac{1}{|\mathrm{C}_2|}(g(C_{\text{fixed}}))^2, \tag{217}$$

for any real function $g(\cdot)$. Now, we proceed to fix the Cliffords in the circuit. The strategy involves fixing a few Cliffords such that: 1) one of the single-qubit Pauli in the Pauli decomposition of P_X is connected with H_μ by a path of Clifford gates (which will be responsible for making the commutator non-zero), 2) the chosen Cliffords 'protect the Pauli', i.e., they ensure the Pauli weight $|P|$ does not increase throughout the application of the unitary layer, 3) at each layer iteratively, as done in Eq. (216), we select only the Pauli operators that have all X in their support that arises when we take the adjoint of the noise. Thus, this would give rise to the lower bound

$$\mathbb{E}[(f_0(P_X))^2] \geq \frac{1}{|\mathrm{C}_2|^{\#\,\text{fixed clifford}}} D_X^{2|P|(L-k+1)}\mathbb{E}[(f_k(\mathcal{V}^{\text{single}}(\tilde{P}_X))^2], \tag{218}$$

where $\tilde{P}_X$ represents the P_X Pauli operator that has been mapped by all the Clifford circuit, and $\mathcal{V}^{\text{single}}$ is a single-qubit layer of random gates. The term $(D_X^{2|P|})^{(L-k+1)}$ represents the factor obtained at each of the $L-k+1$ layers when encountering a layer of noise, applying Eq. (216), and utilizing the fact that the Pauli weight does not increase in the 'Clifford path'. Note that

$$f_k(\mathcal{V}^{\text{single}}(\tilde{P}_X)) = \Big(\Phi_{[1,k]}(\rho_0) i\left[H_\mu, \mathcal{V}^{\text{single}}(\tilde{P}_X)\right]\Big). \tag{219}$$

We now show the existence of this particular Clifford gates choice that satisfy the listed desiderata, by fixing some of the 2-qubit gates to be the identity gate or the SWAP gate (noting that the SWAP gate is Clifford, as it can be expressed as a combination of 3 CNOT gates).

Since H_μ is in the light-cone with respect to $\Phi_{[k,L]}$, by definition, there must exist a path of 2-qubit (Clifford) gates that connects H_μ with one of the single-qubit Paulis X appearing in the tensor product decomposition of P_X. Let us choose one such path connecting H_μ with a specific single-qubit Pauli X. We can fix each 2-qubit gate in this path to be the SWAP gate or the identity gate, in such a way that the X gate has now support overlapping with H_μ. The number of gates in this Clifford path is $L-k+1$. Next, we fix other Cliffords in the circuit to be trivial Identity I Clifford gates, specifically those connecting with the remaining Paulis X in the tensor product decomposition of P_X. See Figure 4 for an example. Consequently, the non-trivial Pauli $\tilde{P}_X$ remains the same up to permutations of its tensor factor: in particular one of its X Paulis is now swapped to a position where it acts non-trivially with H_μ. The count of fixed Cliffords in the circuit is then given by

$$\#\,\text{fixed Cliffords} \leq |P|(L-k+1), \tag{220}$$

since, at each layer (of which there are $L-k+1$), we fix at most one gate for each of the single-qubit Paulis in the Pauli decomposition of P_X. Now, we utilize the last layer of single-qubit random gates to map the resulting Pauli, which now shares support with H_μ, to a Pauli that does not commute with H_μ. Note that such a Pauli exists, as any operator commuting with all the Paulis should be the identity. However, H_μ cannot be the identity because that would contradict the assumption that the support of H_μ is in the light-cone. We denote such new resulting Pauli as Q. Thus, by repeating the same for P_Y and P_Z, and

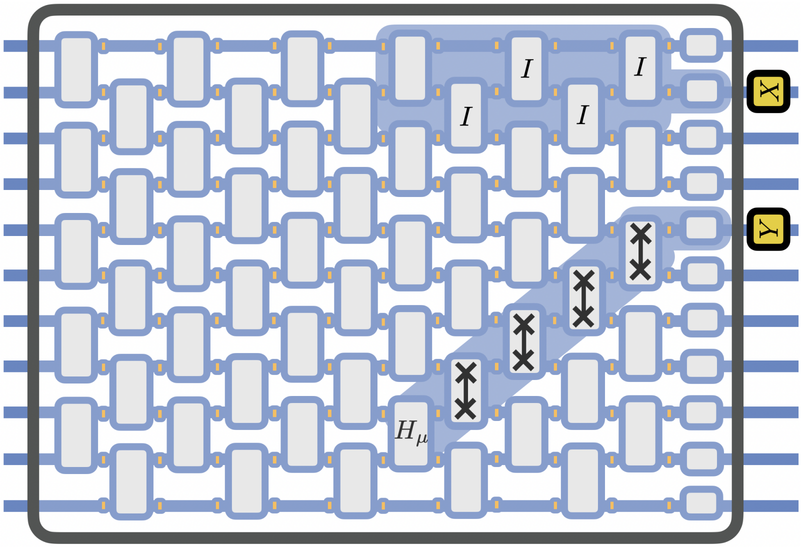

Figure 4. Example of Clifford path choices. The shaded region indicates the fixed Clifford gates. Note that we choose some Clifford gates to be SWAP gates so that they connect one of the Paulis to H_μ. We protect the other remaining Pauli from spreading across the circuit with identity Clifford gates.

mapping them at the end to Q, we have the lower bound

$$\mathrm{Var}[\partial_\mu C] \geq \frac{1}{|\mathrm{C}_2|^{|P|(L-k+1)}} \frac{1}{3^{|P|}} \left(D_X^{2|P|(L-k+1)} + D_Y^{2|P|(L-k+1)} + D_Z^{2|P|(L-k+1)} \right) \mathbb{E}(\mathrm{Tr}\big(\Phi_{[1,k]}(\rho_0) i\,[H_\mu, Q]\big))^2. \tag{221}$$

Since H_μ is a 2-qubit gate, it can be expanded in its 2-qubits Pauli decomposition, as

$$H_\mu = \sum_{R\in\{I,X,Y,Z\}^{\otimes 2}} b_R R, \tag{222}$$

Thus, substituting and using Lemma 5, we have

$$\mathbb{E}(\mathrm{Tr}\big(\Phi_{[1,k]}(\rho_0) i\,[H_\mu, Q]\big))^2 = \sum_{R\in\{I,X,Y,Z\}^{\otimes 2}} b_R^2 \mathbb{E}(\mathrm{Tr}\big(\Phi_{[1,k]}(\rho_0) Q_R\big))^2, \tag{223}$$

where we defined $Q_R := i\,[R, Q]$. Here we note that Q_R is a Hermitian operator, and in particular, it is a Pauli operator (or the zero operator). Thus, we have

$$\begin{aligned}
\mathbb{E}(\mathrm{Tr}\big(\Phi_{[1,k]}(\rho_0) i\,[H_\mu, Q]\big))^2 &= \sum_{R\in\{I,X,Y,Z\}^{\otimes 2}} b_R^2 \mathbb{E}(\mathrm{Tr}\big(\Phi_{[1,k]}(\rho_0) Q_R\big))^2 \\
&\geq \sum_{R\in\{I,X,Y,Z\}^{\otimes 2}} b_R^2 (1-\delta_{[Q,R],\mathbf{0}}) \|\mathbf{t}\|_2^{2|Q_R|}, \\
&\geq \|\mathbf{t}\|_2^{2(|P|+1)} \sum_{R\in\{I,X,Y,Z\}^{\otimes 2}} b_R^2 (1-\delta_{[Q,R],\mathbf{0}}) \\
&\geq \|\mathbf{t}\|_2^{2(|P|+1)} b_{\tilde{R}}^2
\end{aligned} \tag{224}$$

where in the second step we have used the lower bound on variance expectation values derived in Proposition 12 and introduced $\delta_{[Q,R],\mathbf{0}}$ which is one if Q and R commute and zero otherwise, in the third step we observed that $|Q_R| \leq |P|+1$ and used

that $\|\mathbf{t}\|_2 \leq 1$ (Lemma 6). In the last step we have used that, since H_μ does not commute with Q, there must exists $\tilde{R} \in \{I, X, Y, Z\}^{\otimes 2}$ such that $b_{\tilde{R}} \neq 0$ and $[\tilde{R}, Q] \neq 0$. Now we observe that

$$b_{\tilde{R}}^2 \geq \frac{1}{16} \sum_{R \in \{I,X,Y,Z\}^{\otimes 2}} b_R^2 = \frac{1}{64} \|H_\mu\|_2^2 \geq \frac{1}{64} \|H_\mu\|_\infty^2. \tag{225}$$

Putting everything together, we have

$$\mathrm{Var}[\partial_\mu C] \geq \frac{1}{|\mathrm{C}_2|^{|P|(L-k+1)}} \frac{1}{3^{|P|}} \left(D_X^{2|P|(L-k+1)} + D_Y^{2|P|(L-k+1)} + D_Z^{2|P|(L-k+1)} \right) \frac{1}{64} \|H_\mu\|_\infty^2 \|\mathbf{t}\|_2^{2(|P|+1)}.$$

We note that if all the entries of $\mathbf{D} = (D_X, D_Y, D_Z)$ are equal to zero, then the noise is a replacer channel. This because it holds that

$$\mathcal{N}\left(\frac{I + \mathbf{w} \cdot \boldsymbol{\sigma}}{2}\right) = \frac{I}{2} + \frac{1}{2}(\mathbf{t} + D\mathbf{w}) \cdot \boldsymbol{\sigma}, \tag{226}$$

where $D = \mathrm{diag}(\mathbf{D})$, $\mathcal{N}$ is a single-qubit noise channel, and $\frac{I + \mathbf{w} \cdot \boldsymbol{\sigma}}{2}$ represents a density matrix for $\|w\|_2 \leq 1$ (see Eq. (44)). However for assumption, the noise cannot be a replacer channel and so at least one of the entries of $\mathbf{D}$, say D_X, should be nonzero. Further lower bounding, we get

$$\mathrm{Var}[\partial_\mu C] \geq \sum_{Q \in \{X,Y,Z\}} \frac{1}{64} \left(\frac{D_Q^2 \|\mathbf{t}\|_2^2}{3|\mathrm{C}_2|} \right)^{|P|(L-k+1)} \|H_\mu\|_\infty^2. \tag{227}$$

This quantity has the claimed scaling, so we can conclude the proof. $\square$

It might be useful to give the same scaling without the asymptotic notation.

Remark 54 (Scaling without asymptotic notation)**.** *The lower bound (without the asymptotic notation) we found in the previous Proposition 53 on the partial derivative with respect the parameter θ_μ of the gate $\exp(-i\theta_\mu H_\mu)$ in the k-layer of a L-depth circuit is*

$$\mathrm{Var}[\partial_\mu C] \geq \sum_{Q \in \{X,Y,Z\}} \frac{1}{64} \left(\frac{D_Q^2 \|\mathbf{t}\|_2^2}{3|\mathrm{C}_2|} \right)^{|P|(L-k+1)} \|H_\mu\|_\infty^2, \tag{228}$$

where $\mathbf{D}$ and $\mathbf{t}$ are the noise parameters (see Lemma 6), $|\mathrm{C}_2|$ is the size of the 2-qubits Clifford group.

We note that even if the non-unital noise rate $\|\mathbf{t}\|_2$ is polynomially small in the number of qubits, then the derived lower bound still indicates absence of barren plateaus (due to the last few layers). Proposition 53 readily leads to the following conclusion regarding a lower bound on the expected value of the 2-norm of the gradient.

Corollary 55 (Lower bound on the expected value of the 2-norm of the gradient)**.** *Let us consider a cost function associated to a local Pauli P with $|P| = \Theta(1)$, and the same assumption as in Proposition 53. Then, we have*

$$\mathbb{E}[\|\nabla C\|_2^2] \geq \Omega(1). \tag{229}$$

Proof. The proof follows immediately by focusing only on the last parameter θ_m of the last layer (which gate is in the light cone of P). In particular, we have

$$\mathbb{E}[\|\nabla C\|_2^2] \geq \sum_{\mu=1}^{m} \mathbb{E}[\partial_\mu C^2] \geq \mathbb{E}[\partial_m C^2] = \mathrm{Var}[\partial_m C^2] \geq \Omega(1), \tag{230}$$

where we have used the fact that the partial derivative has zero mean and Proposition 53. $\square$

We can now rephrase our previous result in terms of a probability statement.

Corollary 56 (Probability statement)**.** *Assuming that the cost function has a number of free parameters upper bounded by $O(\mathrm{poly}(n))$, we have*

$$\mathrm{Prob}\left(\|\nabla C\|_2^2 > \Omega(1)\right) \geq \Omega(1/\mathrm{poly}(n)). \tag{231}$$

Proof. By applying the probability inequality in Lemma 64 with $f := \|\nabla C\|_2^2$ and utilizing Corollary 55, we arrive at

$$\mathrm{Prob}\left(\|\nabla C\|_2^2 > \Omega(1)\right) \geq \frac{\Omega(1)}{\sup(|\|\nabla C\|_2^2|)}. \tag{232}$$

In order to conclude, we need to establish an upper bound for $\sup(|\|\nabla C\|_2^2|)$. This upper bound can be derived as

$$\sup(|\|\nabla C\|_2^2|) \leq m \max_{\mu\in[m]} (\partial_\mu C)^2 \leq 4m \max_{\mu\in[m]} \|H_\mu\|_\infty^2 \|P\|_\infty^2 \leq 4m. \tag{233}$$

In this equation, m represents the number of parameters, and we employ Lemma 44 in the final step. □

In summary, we have shown that the probability of sampling an instance of a circuit in which the gradient is larger than a constant is not exponentially small. However, it is important to stress that achieving a large average gradient norm can be accomplished by focusing on the last layers. In fact, the components corresponding to the initial layers of a linear depth circuit are exponentially small.

D. Improved upper bounds for unital noise

In this section, we present improved upper bounds on the barren plateaus phenomenon in the context of random quantum circuit ansatz with unital noise. Our derived bounds are tighter compared to those presented in Ref. [50]. Notably, our approach leverages the randomness of the circuit, whereas Ref. [50] relies solely on the contraction property of the unital noise channel analyzed. We start by showing the variance of expectation values in the case of random quantum circuits with unital noise. Up to our knowledge, this was not known before. The noiseless case was instead addressed in Ref. [30]. In the following, due to Lemma 5, we can focus on Pauli observables without loss of generality.

Proposition 57 (Improved expectation values concentration for unital noise)**.** *Let $P \in \{I,X,Y,Z\}^{\otimes n}$, ρ_0 be any quantum state, and L be the depth of the noisy circuit Φ defined in Eq. (55) in arbitrary dimension. Specifically, we assume that the noise is unital. Then, we have*

$$\mathrm{Var}[\mathrm{Tr}(P\Phi(\rho_0))] \leq 4c^{|P|+L-1}, \tag{234}$$

where the parameter c is defined in Eq. (76).

Proof. Because our circuit ends with a layer of random single-qubit gates, it holds that $\mathbb{E}[\mathrm{Tr}(P\Phi(\rho))] = 0$, following from Lemma 4. Thus, we focus on $\mathbb{E}[\mathrm{Tr}(P\Phi(\rho_0))^2]$. We have

$$\mathbb{E}[\mathrm{Tr}(P\Phi(\rho_0))^2] = \mathbb{E}\left[\mathrm{Tr}\left(P\Phi\left(\rho_0 - \frac{I_n}{2^n}\right)\right)^2\right], \tag{235}$$

where we have used the unitality of the noise channels to get $\Phi(I_n) = I_n$, and the fact that the Pauli operators are traceless. The claim follows by applying the effective depth Theorem 16. □

We now show an upper bound on the partial derivative variance in the case of unital noise. Due to Lemma 42, we can focus on Pauli observables without loss of generality.

Proposition 58 (Improved upper bound on the partial derivative for unital noise)**.** *Let $C := \mathrm{Tr}(P\Phi(\rho_0))$ be the cost function, where $P \in \{I,X,Y,Z\}^{\otimes n}$, ρ_0 is an arbitrary initial state, and Φ is a quantum circuit of depth L in arbitrary dimension. We assume that the noise is unital. Let μ denote a parameter of any 2-qubit gate $\exp(-i\theta_\mu H_\mu)$ in the circuit such that $\|H_\mu\|_\infty \leq 1$. Then, we have*

$$\mathrm{Var}[\partial_\mu C] \leq 4c^{|P|+L-1}. \tag{236}$$

Proof. By repeating the same steps of the proof of Proposition 14, namely the upper bound on the variance for non-unital noise, we get

$$\mathrm{Var}[\partial_\mu C] = \mathbb{E}[(\partial_\mu C)^2] \leq c^{|P|+L-k-1} \max_{Q\in\{I,X,Y,Z\}^{\otimes n}\setminus I_n} \mathbb{E}(\mathrm{Tr}(\Phi_{[1,k]}(\rho_0)i[H_\mu, Q]))^2. \tag{237}$$

Since H_μ is a 2-qubit gate, it can be expanded in its 2-qubits Pauli decomposition, as

$$H_\mu = \sum_{R\in\{I,X,Y,Z\}^{\otimes 2}} b_R R. \tag{238}$$

Thus, substituting and using Lemma 5, we have

$$\mathbb{E}(\mathrm{Tr}\big(\Phi_{[1,k]}(\rho_0) i\,[H_\mu, Q]\big))^2 = \sum_{R\in\{I,X,Y,Z\}^{\otimes 2}} b_R^2 \mathbb{E}(\mathrm{Tr}\big(\Phi_{[1,k]}(\rho_0) Q_R\big))^2. \tag{239}$$

Here, we note that $Q_R := i\,[R, Q]$ is a Hermitian operator, and in particular, it is a Pauli operator (or the zero operator). Thus, we have

$$\begin{aligned}\mathbb{E}(\mathrm{Tr}\big(\Phi_{[1,k]}(\rho_0) i\,[H_\mu, Q]\big))^2 &= \sum_{R\in\{I,X,Y,Z\}^{\otimes 2}} b_R^2 \mathbb{E}(\mathrm{Tr}\big(\Phi_{[1,k]}(\rho_0) Q_R\big))^2 \\ &\le c^k \sum_{R\in\{I,X,Y,Z\}^{\otimes 2}} b_R^2,\end{aligned} \tag{240}$$

where in the last step we have used Proposition 57 and that the Pauli weight of the non-zero Pauli is lower bounded by one. Now we observe that

$$\sum_{R\in\{I,X,Y,Z\}^{\otimes 2}} b_R^2 = \frac{1}{4}\|H_\mu\|_2^2 \le \|H_\mu\|_\infty^2 \le 1, \tag{241}$$

which concludes the proof. □

This result improves upon the partial derivative variance upper bound presented in Ref. [50], where the upper bound scaled as

$$\mathrm{Var}[\partial_\mu C] = O(n^{1/2} 2^{-\alpha L}), \tag{242}$$

for some positive constant α. It is noteworthy that this latter upper bound has no dependence on the Pauli weight, unlike ours. Furthermore, it includes a $n^{1/2}$ factor in front of the exponential, making it meaningful only at depths $\Omega(\log(n))$. Moreover, our result is more general than that shown in Ref. [50] also because it extends to any unital noise, whereas the results shown in Ref. [50] apply only to primitive unital noise, which is only a particular type of unital noise (e.g., dephasing is not included in this class).

V. Purity and kernel methods under non-unital noise

When a circuit is interspersed with primitive, unital noise, the decay in purity can be investigated by employing well-known entropy accumulation techniques (see, for instance, Refs. [15, 77–80]). However, this approach has limited applicability under non-unital noise, as the noise channel can potentially decrease the entropy of the system. Here, we address this gap in the literature by providing upper and lower bounds on the purity of a noisy circuit, leveraging prior techniques along with the tools developed in the present work. As an application, we employ our upper bounds to investigate the limitations of quantum kernel methods under non-unital noise.

A. Purity of average and worst-case circuits

In this section, we explore the decay in purity under non-unital noise. We propose two distinct approaches: first, we provide upper and lower bounds for average-case circuits under possibly non-unital noise; second, we provide upper bounds for worst-case circuits, under the further assumption that the noise channel can be decomposed into a depolarizing channel followed by an arbitrary channel.

1. *Purity of an average-case noisy circuit*

We now upper and lower bound the expected purity of the output state of a noisy circuit, as defined in Eq. (55).

Proposition 59 (Average-case upper and lower bounds on the purity)**.** *Let ρ be a quantum state. Then, at any depth of the noisy circuit Φ, we have*

$$\left(\frac{1+\|\mathbf{t}\|_2^2}{2}\right)^n \leq \mathbb{E}\,\mathrm{Tr}\left[\Phi(\rho)^2\right] \leq \left(\frac{1+\|\mathbf{t}\|_2^2+\|\mathbf{D}\|_2^2}{2}\right)^n. \tag{243}$$

Proof. We first recall that the purity can be expressed in the Pauli basis as

$$\mathrm{Tr}\left[\Phi(\rho)^2\right] = \mathrm{Tr}\left[\mathbb{F}\Phi(\rho)^{\otimes 2}\right] = \frac{1}{2^n}\sum_{P\in\{I,X,Y,Z\}^{\otimes n}} \mathrm{Tr}\left[P^{\otimes 2}\Phi(\rho)^{\otimes 2}\right] = \frac{1}{2^n}\sum_{P\in\{I,X,Y,Z\}^{\otimes n}} \mathrm{Tr}[P\Phi(\rho)]^2. \tag{244}$$

Hence, plugging the upper and lower bound on the expected second moments (Eqs. 66, 82) yields the desired results

$$\begin{aligned}\mathbb{E}\,\mathrm{Tr}\left[\Phi(\rho)^2\right] &= \frac{1}{2^n}\sum_{P\in\{I,X,Y,Z\}^{\otimes n}} \mathbb{E}\,\mathrm{Tr}[P\Phi(\rho)]^2 \leq \sum_{P\in\{I,X,Y,Z\}^{\otimes n}} \left(\frac{\|\mathbf{t}\|_2^2+\|\mathbf{D}\|_2^2}{3}\right)^{|P|} \\ &= \frac{1}{2^n}\sum_{k=0}^{n}\binom{n}{k}\left(\|\mathbf{t}\|_2^2+\|\mathbf{D}\|_2^2\right)^k = \left(\frac{1+\|\mathbf{t}\|_2^2+\|\mathbf{D}\|_2^2}{2}\right)^n,\end{aligned} \tag{245}$$

and

$$\begin{aligned}\mathbb{E}\,\mathrm{Tr}\left[\Phi(\rho)^2\right] &= \frac{1}{2^n}\sum_{P\in\{I,X,Y,Z\}^{\otimes n}} \mathbb{E}\,\mathrm{Tr}[P\Phi(\rho)]^2 \geq \sum_{P\in\{I,X,Y,Z\}^{\otimes n}} \left(\frac{\|\mathbf{t}\|_2^2}{3}\right)^{|P|} \\ &= \frac{1}{2^n}\sum_{k=0}^{n}\binom{n}{k}\|\mathbf{t}\|_2^{2k} = \left(\frac{1+\|\mathbf{t}\|_2^2}{2}\right)^n,\end{aligned} \tag{246}$$

which ends the proof. □

In particular, if $\mathcal{N}$ is a unital non-unitary channel, we have $\|\mathbf{t}\|_2^2 = 0$ and $\|\mathbf{D}\|_2^2 < 1$, which implies

$$\mathbb{E}\,\mathrm{Tr}\left[\Phi(\rho)^2\right] \leq 2^{-\Omega(n)}. \tag{247}$$

We observe that the bounds given in Proposition 59 hold also for reduced states, that is states obtained by performing a partial trace on the output state of the circuit. In particular, for any arbitrary state ρ, we let $\rho_S := \mathrm{Tr}_{[n]\setminus S}[\rho]$ the reduced state on a subset S of the qubits of size $|S| = k$. Then we have

$$\left(\frac{1+\|\mathbf{t}\|_2^2}{2}\right)^k \leq \mathbb{E}\,\mathrm{Tr}\left[\Phi(\rho)_S^2\right] \leq \left(\frac{1+\|\mathbf{t}\|_2^2+\|\mathbf{D}\|_2^2}{2}\right)^k. \tag{248}$$

2. Purity of a worst-case noisy circuit

In this section, we consider a layered circuit $\mathcal{C}$ of the form

$$\mathcal{C} = \widetilde{\mathcal{N}}^{\otimes n} \circ \mathcal{U}_L \circ \cdots \circ \widetilde{\mathcal{N}}^{\otimes n} \circ \mathcal{U}_1, \tag{249}$$

where we do not make any assumption on the structure of each unitary layer $\mathcal{U}_i = U_i^\dagger(\cdot)U_i$. In contrast, we will make a further assumption on the noise channel. In particular, we will model the local noise as the composition of two single-qubit channels, namely a local depolarizing channel $\mathcal{N}_p^{(\mathrm{dep})}(X) = p\frac{I}{2}\operatorname{Tr}[X] + (1-p)X$ and arbitrary noise channel $\mathcal{N}$ expressed in the normal form, i.e.,

$$\widetilde{\mathcal{N}} = \mathcal{N} \circ \mathcal{N}_p^{(\mathrm{dep})}, \tag{250}$$

where $\mathcal{N}(I + \mathbf{w}\cdot\boldsymbol{\sigma}) = I + (\mathbf{t} + D\mathbf{w})\cdot\boldsymbol{\sigma}$. Under this stronger assumption, we provide two upper bounds on the purity of a worst-case noisy circuit. We also remark that order of $\mathcal{N}$ and $\mathcal{N}_p^{(\mathrm{dep})}(X)$ does not play a central role in our analysis, therefore the same results could be derived inverting their order.

Let us recall the definition of quantum relative entropy and quantum sandwiched Rényi divergence [81, 82]. Let ρ, σ be two quantum states. If $\operatorname{supp}(\rho) \subseteq \operatorname{supp}(\sigma)$, we define the quantum relative entropy as

$$D(\rho\|\sigma) := \operatorname{Tr}(\rho\log\rho) - \operatorname{Tr}(\rho\log\sigma). \tag{251}$$

For a parameter $\alpha \in (0,1)\cup(1,\infty)$, the quantum Rényi divergence of order α is defined as

$$D_\alpha(\rho\|\sigma) := \frac{1}{\alpha-1}\log\operatorname{Tr}\left[\left(\sigma^{\frac{1-\alpha}{2\alpha}}\rho\sigma^{\frac{1-\alpha}{2\alpha}}\right)^\alpha\right]. \tag{252}$$

This definition applies when $\operatorname{supp}(\rho) \subseteq \operatorname{supp}(\sigma)$, for $\alpha \in (1,\infty)$. In the limit $\alpha \to 1$, the quantum Rényi divergence reduces to the quantum relative entropy, i.e., $\lim_{\alpha\to1} D_\alpha(\rho\|\sigma) = D(\rho\|\sigma)$. The ∞-relative entropy is defined, for $\operatorname{supp}(\rho) \subseteq \operatorname{supp}(\sigma)$, as

$$D_\infty(\rho\|\sigma) := \inf\{\gamma : \rho \le 2^\gamma\sigma\}. \tag{253}$$

It is useful to recall that for $\alpha > \beta > 0$, the Rényi divergences satisfy the monotonicity property, i.e., $D_\alpha(\rho\|\sigma) \ge D_\beta(\rho\|\sigma)$.

By mean of the *data-processed triangle inequality* ([83], Theorem 3.1), the authors of Refs. [15, 79], obtained an upper bound on the purity of the output of a non-unital channel, which is exponentially small in n when the unital component of the noise 'dominates' the non-unital one. We rephrase such result within our model, giving an explicit expression in terms of p and $\mathbf{t}$.

Corollary 60 (Worst-case upper bound on the purity)**.** *Let ρ an arbitrary quantum state and assume $p > 0$ and $\|\mathbf{t}\|_2 \neq 1$. Then for any constant noise parameters, we have*

$$D_2\left(\mathcal{C}(\rho)\middle\|\frac{I}{2^n}\right) \le n\left((1-p)^{2L} + \|\mathbf{t}\|_2\frac{1-(1-p)^{2L}}{2p-p^2}\right) := n\cdot\delta_L. \tag{254}$$

This implies the following upper bound on the purity

$$\operatorname{Tr}\left[\mathcal{C}(\rho)^2\right] \le 2^{n(\delta_L-1)}. \tag{255}$$

Proof. We first recall that $\operatorname{Tr}\left[\rho^2\right] = 2^{-n+D_2(\rho\|I/2^n)}$, then first bound implies the second. We note the following

$$D_\infty\left(\mathcal{N}^{\otimes n}\left(\frac{I}{2^n}\right)\middle\|\frac{I}{2^n}\right) = nD_\infty\left(\mathcal{N}\left(\frac{I}{2}\right)\middle\|\frac{I}{2}\right) = n\log(1+\|\mathbf{t}\|_2) \le n\|\mathbf{t}\|_2, \tag{256}$$

where the second identity is a special case of Lemma 23 in Ref. [84]. Moreover, Lemma C.1 in Ref. [79] ensures

$$D_2\left(\mathcal{C}(\rho)\middle\|\frac{I}{2^n}\right) \le (1-p)^{2L}D_2\left(\rho\middle\|\frac{I}{2^n}\right) + \sum_{t=0}^{L}(1-p)^{2t}D_\infty\left(\mathcal{N}^{\otimes n}\left(\frac{I}{2^n}\right)\middle\|\frac{I}{2^n}\right). \tag{257}$$

Then the desired upper bound on $D_2\left(\mathcal{C}(\rho)\|I/2^n\right)$ immediately follows. □

Note that the term δ_L converges exponentially fast to $\|\mathbf{t}\|_2/(2p-p^2)$, and thus in this regime the bound is non-trivial if $\|\mathbf{t}\|_2 \le 2p - p^2$.

B. Quantum machine learning under non-unital noise: Kernel methods

Quantum kernel methods offer a hopeful avenue for advancing quantum machine learning. However, despite certain positive results, as documented in Ref. [85], these methods remain susceptible to trainability challenges. In particular, the work of Thanasilp *et al.* [86] has demonstrated that various factors, such as circuit randomness and unital noise, can potentially compromise their trainability, in analogy to the phenomenon of barren plateaus for cost functions. Here we incorporate both unital and non-unital noise in our analysis and we show that fidelity kernels exponentially concentrate even at constant depth. This starkly improves the result of Ref. ([86], Theorem 3) , which predicts exponential concentration at linear depth for unital noise.

1. *Preliminaries on quantum kernel methods*

Consider an n-qubit data-embedding channel $\Phi_{\boldsymbol{x}}$ parametrized by a point $\boldsymbol{x} \in \mathcal{X}$, so that

$$\rho(\boldsymbol{x}) = \Phi_{\boldsymbol{x}}(\rho_0), \tag{258}$$

where ρ_0 is the initial state of the circuit, usually set as $\rho_0 = |0^n\rangle\langle 0^n|$. A kernel $\kappa : \mathcal{X} \times \mathcal{X} \to \mathbb{R}^+$ is a similarity measure between pair of points $\boldsymbol{x}, \boldsymbol{y} \in \mathcal{X}$. In particular, quantum kernels rely on the quantum embedding scheme described in the Equation 258 above. We consider the fidelity quantum kernel [87, 88], defined as

$$\kappa^{FQ}(\boldsymbol{x}, \boldsymbol{y}) = \mathrm{Tr}[\rho(\boldsymbol{x})\rho(\boldsymbol{y})]. \tag{259}$$

Kernel-based learning methods are notable for their capacity to transform data from the original space $\mathcal{X}$ into a higher-dimensional feature space, which in our case coincides with the a 2^n-dimensional Hilbert space. In this new feature space, inner products are computed, enabling the training of decision boundaries like support vector machines, as explained in reference [88].

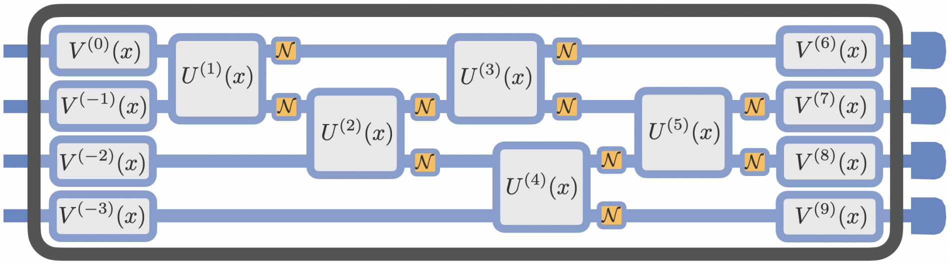

Figure 5. Example of a noisy quantum circuit on $n = 4$ qubits with two-qubit and single-qubit gates, parametrized by the input vector $\boldsymbol{x} \in \mathcal{X}$.

2. *Kernel-based supervised learning*

To better suite our results, we sketch how kernel methods can be used to perform supervised learning. We consider a training set of labelled inputs $\mathcal{S} = \left\{\boldsymbol{x}^{(i)}, f\left(\boldsymbol{x}^{(i)}\right)\right\}_{i\in[m]}$, where $f(\cdot)$ is some unknown function that we want to learn. Thus our goal is to find a function h approximating f. Thanks to the Representer Theorem (see, for instance, Ref. [89], Theorem 16.1), the optimal function can be expressed as

$$h(z) = \sum_{i=1}^{m} a_i \kappa\left(\boldsymbol{x}^{(i)}, z\right), \tag{260}$$

where the $\boldsymbol{a} = (a_1, a_2, \ldots, a_m)$ is a vector of parameters to be optimized with respect to a suitable loss function.

Then, to enable the implementation of kernel methods, it is necessary to estimate the Gram matrix. This matrix, denoted as $\mathcal{G}$, comprises the kernels derived from pairs of inputs within the training set $\boldsymbol{x}^{(1)}, \boldsymbol{x}^{(2)}, \ldots, \boldsymbol{x}^{(m)}$, and is defined as

$$\forall i \in [m] : \mathcal{G}[i,j] = \kappa\left(\boldsymbol{x}^{(i)}, \boldsymbol{x}^{(j)}\right). \tag{261}$$

We recall that kernels exhibit exponential concentration with respect to a distribution $\mathcal{D}$ over $\mathcal{X}$, if there exists a real number $\mu \in \mathbb{R}$ and a value $\delta \in 2^{-\Omega(n)}$ such that

$$\Pr_{\boldsymbol{x},\boldsymbol{y}\sim\mathcal{D}}[|\kappa(\boldsymbol{x},\boldsymbol{y}) - \mu| \geq \delta] \in 2^{-\Omega(n)}. \tag{262}$$

In this case, all the entries of the Gram matrix are exponentially close to μ with exponentially high probability, making the optimization of the vector $\boldsymbol{a}$ an information-theoretically hard task.

3. *Assumption on the training data distribution.*

We assume that each point in the training set is sampled from a distribution $\mathcal{D} : \mathcal{X} \to [0,1]$ and we denote by ν' the corresponding induced distribution over quantum channels. Moreover, we also assume that ν' satisfies Definition 8, i.e., it is 2-local 2-design layer distribution.

4. *Fidelity quantum kernels: Exponential concentration at any depth*

The Cauchy-Schwarz inequality implies that the fidelity quantum kernel can be upper bounded by the square root of the purities of the output states

$$\mathrm{Tr}[\rho(\boldsymbol{x})\rho(\boldsymbol{x'})] \leq \sqrt{\mathrm{Tr}[\rho(\boldsymbol{x})^2]\,\mathrm{Tr}[\rho(\boldsymbol{x'})^2]}. \tag{263}$$

By a direct application Proposition 59, we obtain the following result.

Corollary 61 (Exponential concentration of quantum kernels, average-case circuit)**.**

$$\mathbb{E}_{\boldsymbol{x},\boldsymbol{x'}}\kappa^{FQ}(\boldsymbol{x},\boldsymbol{x'}) \leq \left(\frac{1+\|\mathbf{t}\|_2^2+\|\mathbf{D}\|_2^2}{2}\right)^n. \tag{264}$$

In a similar fashion, we can derive a worst-case concentration bound by employing Corollary 60.

Corollary 62 (Exponential concentration of quantum kernels, worst-case circuit)**.** *Let $\mathcal{U}_x, \mathcal{U}_{x'}$ two noisy circuits interspersed by L layers of local noise, modeled by the channel $\mathcal{N}^{\otimes n}\circ\mathcal{N}_p^{(\mathrm{dep})\otimes n}$. Denote by $\rho(\boldsymbol{x}) = \mathcal{U}_{\boldsymbol{x}}(\rho_0)$ and $\rho(\boldsymbol{x'}) = \mathcal{U}_{\boldsymbol{x'}}(\rho_0)$ the output states of the noisy circuits. Assume that $p = \Theta(1)$ and $\|\mathbf{t}\|_2 \neq 1$. Then the fidelity quantum kernel $\kappa^{FQ}(\boldsymbol{x},\boldsymbol{x'}) = \mathrm{Tr}[\rho(\boldsymbol{x})\rho(\boldsymbol{x'})]$ satisfies the upper bound*

$$\kappa^{FQ}(\boldsymbol{x},\boldsymbol{x'}) \leq 2^{n(\delta_L-1)}, \tag{265}$$

where $\delta_L := (1-p)^{2L} + \|\mathbf{t}\|_2\frac{1-(1-p)^{2L}}{2p-p^2}$.

As mentioned in Subsection V A 2, this bound can be exponentially vanishing in the number of qubits for certain range of parameters.

We also emphasize when $\|\mathbf{t}\| = 0$, our bound predicts that the kernel $\kappa^{FQ}(\boldsymbol{x},\boldsymbol{x'})$ is at most $2^{-n(2p-p^2)} = 2^{-\Omega(n)}$, even after a single layer of noise, whereas Theorem 3 in Ref. [86] only predicts that $|\kappa^{FQ}(x,x') - 1/2^n| \leq (1-p)^2 = \Theta(1)$. Thus, compared to the previous literature, our result is exponentially tighter with respect to the number of layers for the local depolarizing noise. An analogous bound for local Pauli noise can be derived along the lines of Supplementary Lemma 6 in Ref. [50].

VI. Miscellaneous

In what follows, we present a number of useful lemmas.

Lemma 63 (Large variance implies significant probability of deviation)**.** *Let f be a real function depending by parameters distributed according to a probability distribution μ. Then, the inequality*

$$\mathrm{Prob}\left(|f-\mathbb{E}[f]| > \sqrt{\frac{\mathrm{Var}[f]}{2}}\right) \geq \frac{\mathrm{Var}[f]}{8\sup(|f|)^2} \tag{266}$$

holds, where the expected value and variance are taken with respect the probability distribution μ.

Proof. Let $T > 0$ be a real value that we will fix later. We have

$$\begin{aligned}\mathrm{Var}[f] &:= \int (f - \mathbb{E}[f])^2 \, d\mu \\ &= \int_{|f-\mathbb{E}[f]|\leq T} (f - \mathbb{E}[f])^2 \, d\mu + \int_{|f-\mathbb{E}[f]|>T} (f - \mathbb{E}[f])^2 \, d\mu \\ &\leq T^2 \int_{|f-\mathbb{E}[f]|\leq T} 1 \, d\mu + \int_{|f-\mathbb{E}[f]|>T} (f - \mathbb{E}[f])^2 \, d\mu \\ &\leq T^2 \left(1 - \int_{|f-\mathbb{E}[f]|>T} 1 \, d\mu\right) + 4(\sup(|f|))^2 \int_{|f-\mathbb{E}[f]|>T} 1 d\mu \\ &= T^2 + \left(4 (\sup(|f|))^2 - T^2\right) \mathrm{Prob}\left(|f - \mathbb{E}[f]| > T\right),\end{aligned} \tag{267}$$

where in the fourth step we have used that $|f - \mathbb{E}[f]| \leq |f| + |\mathbb{E}[f]| \leq 2\sup(|f|)$. Therefore, rearranging the previous inequality, we have

$$\mathrm{Prob}\left(|f - \mathbb{E}[f]| > T\right) \geq \frac{\mathrm{Var}[f] - T^2}{4\sup(|f|)^2 - T^2}. \tag{268}$$

By choosing $T := \frac{1}{\sqrt{2}}\sqrt{\mathrm{Var}[f]}$, we get

$$\mathrm{Prob}\left(|f - \mathbb{E}[f]| > \sqrt{\frac{\mathrm{Var}[f]}{2}}\right) \geq \frac{\mathrm{Var}[f]}{8\sup(|f|)^2 - \mathrm{Var}[f]} \geq \frac{\mathrm{Var}[f]}{8\sup(|f|)^2}. \tag{269}$$

□

Lemma 64 (Large first moments)**.** *Let f be a real function depending by parameters distributed according to a probability distribution μ. Then, the inequality*

$$\mathrm{Prob}\left(f > \frac{\mathbb{E}[f]}{2}\right) \geq \frac{\mathbb{E}[f]}{2\sup(|f|)} \tag{270}$$

holds, where the expected value and variance are taken with respect the probability distribution μ.

Proof. Let T a real value $T > 0$. Then, we have

$$\mathbb{E}[f] = \int f d\mu = \int_{|f|\leq T} f d\mu + \int_{|f|>T} f d\mu \leq T + \sup(|f|)\,\mathrm{Prob}\left(f > T\right). \tag{271}$$

Now, if we assume $T = \mathbb{E}[f]/2$ and rearrange the inequality, we obtain

$$\mathrm{Prob}\left(f > \frac{\mathbb{E}[f]}{2}\right) \geq \frac{\mathbb{E}[f]}{2\sup(|f|)}. \tag{272}$$

□

A. Trace distance decay for worst-case circuits under local depolarizing noise

As documented by the previous literature [50, 53, 80], the output of any circuit affected by unital, primitive noise converges exponentially fast in the depth to the maximally mixed state with respect to the trace distance. The proof relies on the Pinsker's inequality and on the contraction coefficients of the quantum Rényi divergence of order 2. See the definition of Rényi divergence in Eq. (252). For the sake of simplicity, we will consider the special case of the depolarizing noise, and refer to Refs. [50, 80] for an extension to arbitrary Pauli channels with normal form parameters satisfying $D_P < 1$ for all $P \in \{X, Y, Z\}$. We will need the following lemma.

Lemma 65 (Strong data-processing inequality. Adapted from Theorem 6.1 in Ref. [53])**.** *Let $\mathcal{N}_p^{(\mathrm{dep})}$ be the single-qubit depolarizing channel of rate p, i.e., $\mathcal{N}_p^{(\mathrm{dep})}(X) = p\frac{I}{2}\mathrm{Tr}(X) + (1-p)X$. Let $\Phi := \bigcirc_{i=1}^{L}(\mathcal{N}_p^{(\mathrm{dep})\otimes n} \circ \mathcal{U}_i)$ be a circuit of L unitary layers interspersed by local depolarizing noise. Then, for every state ρ, we have*

$$D_2\left(\Phi(\rho) \middle\| \frac{I}{2^n}\right) \leq (1-p)^{2L} n. \tag{273}$$

Then the desired result follows by a direct application of Pinsker's inequality.

Proposition 66 (Deviation from maximal mixedness)**.** *Under the same assumptions of Lemma 65, we obtain*

$$\left\| \Phi(\rho) - \frac{I}{2^n} \right\|_1 \leq \sqrt{2n}(1-p)^L. \tag{274}$$

And therefore, for all states ρ and σ, we have

$$\left\| \Phi(\rho) - \Phi(\sigma) \right\|_1 \leq 2\sqrt{2n}(1-p)^L. \tag{275}$$

Proof. Combining Pinsker's inequality with the monotonicity of the the quantum Rényi divergence, we obtain

$$\left\| \Phi(\rho) - \frac{I}{2^n} \right\|_1^2 \leq 2D\left(\Phi(\rho) \middle\| \frac{I}{2^n} \right) \leq 2D_2\left(\Phi(\rho) \middle\| \frac{I}{2^n} \right). \tag{276}$$

Hence, Proposition 66 implies

$$\left\| \Phi(\rho) - \frac{I}{2^n} \right\|_1 \leq \sqrt{2n}(1-p)^L. \tag{277}$$

Thus, a direct application of the triangle inequality yields the desired result

$$\left\| \Phi(\rho) - \Phi(\sigma) \right\|_1 \leq \left\| \Phi(\rho) - \frac{I}{2^n} \right\|_1 + \left\| \Phi(\sigma) - \frac{I}{2^n} \right\|_1 \leq 2\sqrt{2n}(1-p)^L. \tag{278}$$

□

We emphasize that the above result does not require randomness, unlike our Theorem 10. On the other hand, Theorem 10 yields non trivial bound even at constant depth, while the above depolarizing-noise result is vacuous at sub-logarithmic depth. We remark that a non-vacuous bound for shallow depths could be derived by means of the quantum Bretagnolle-Huber inequality (see, for instance, Ref. [90], (Lemma B.1 in Ref. [91]) and references therein).

Informally, Proposition 66 says that the output of a noisy circuit becomes computationally trivial at super-logarithmic depths, provided that the noise is unital and primitive. This poses severe constraints on the capabilities of noisy devices, as exemplified by the following result.

Corollary 67 (Exponential concentration, unital case)**.** *Under the same assumptions of Lemma 65, for every state ρ and for every observable O, we obtain*

$$\left| \mathrm{Tr}[O\Phi(\rho)] - \frac{\mathrm{Tr}[O]}{2^n} \right| \leq \sqrt{2n}(1-p)^L \left\| O \right\|_\infty. \tag{279}$$

Proof. First, we notice that the LHS can be rearranged as

$$\left| \mathrm{Tr}[O\Phi(\rho)] - \frac{\mathrm{Tr}[O]}{2^n} \right| = \left| \mathrm{Tr}\left[O\left(\Phi(\rho) - \frac{1}{2^n} \right) \right] \right|. \tag{280}$$

Hence, we obtain

$$\left| \mathrm{Tr}\left[O\left(\Phi(\rho) - \frac{1}{2^n} \right) \right] \right| \leq \left\| \Phi(\rho) - \frac{I}{2^n} \right\|_1 \left\| O \right\|_\infty \leq \sqrt{2n}(1-p)^L \left\| O \right\|_\infty, \tag{281}$$

where the first inequality follows from the Hölder's inequality, and the second one is a consequence of Proposition 66. □

B. Numerical simulations

In this section we present numerical results that corroborate our analytical results and explore regimes that go beyond the assumptions and results of our theorems, such as the one of assuming the 2-qubit gates to be distributed according to a local 2-design. We start by considering a brickwork architecture as depicted in Fig. 1, where each 2-qubit gate takes the form

$$U_{i,i+1}(\theta_1, \theta_2, \theta_3, \theta_4) := (R_Y(\theta_4) \otimes R_Y(\theta_3))\, \mathrm{CNOT}_{i,i+1}\, (R_X(\theta_2) \otimes R_X(\theta_1)). \tag{282}$$

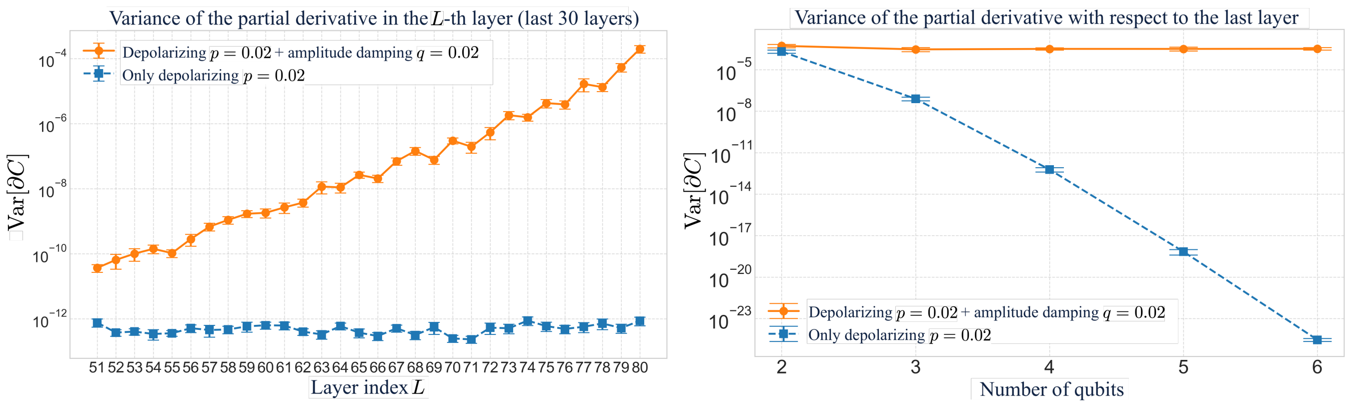

Figure 6. **Left:** Variance of the partial derivative with respect to a gate in the L-th layer of a 4-qubit one-dimensional quantum circuit of depth 80. The expectation value becomes exponentially less *sensitive* to gates far from the end of the circuit. Noise parameters are set to $p = q = 0.02$. When amplitude damping is switched off ($q = 0$), all partial derivatives exhibit comparable scaling across layers. All circuit parameters are sampled uniformly in $[0, 2\pi)$. **Right:** Variance of the partial derivative in the last layer versus the number of qubits, for circuit depth $20n$. With both amplitude damping and depolarizing noise ($p = q = 0.02$), the last-layer variance remains constant with system size, whereas for $q = 0$ it decays exponentially. In both plots, error bars indicate the standard deviation over 20 random parameter samples, with all parameters drawn uniformly from $[0, 2\pi)$. Error bars indicate the standard error of the variance estimate.

We consider the noise model given by the composition of amplitude damping and depolarizing channels given by

$$\mathcal{N}^{(\mathrm{dep,amp})}_{(p,q)} := \mathcal{N}^{(\mathrm{dep})}_{p} \circ \mathcal{N}^{(\mathrm{amp})}_{q}, \tag{283}$$

where two noise channels are defined respectively in Eq. (53) and Eq. (52) with $p, q \in [0, 1]$.

Moreover, we assume, in contrast to our circuit model in Fig. 1, that the circuit ends with a layer of noise (instead of a layer of single-qubits gates). Furthermore, we consider an expectation value with respect to the observable Z_1.

In Fig. 6 (left), we can clearly observe that the partial derivatives taken at the end of the circuit are significantly larger compared to those taken at the beginning of the circuit. This confirms the exponential decay that we proved in Theorem 51. It is noteworthy that if we were to deactivate the amplitude damping component, we would observe an average exponential concentration in all partial derivatives, regardless of the layer at which the derivative is taken. This aligns with the findings of the study on (depolarizing-)noise-induced barren plateaus [50] and with our Proposition 58. Furthermore, in our experiments, we observe that the partial derivatives in the final layers remain constant as the number of qubits increases, as depicted in Fig. 6 (right). This observation corroborates the conclusions drawn in our Corollary 50. In particular, this implies that the 2-norm of the gradient remains constant on average with respect to the number of qubits. Another consideration is whether our theorems heavily depend on the assumption that the 2-qubit gate is sampled by a 2-design, and whether this assumption can be relaxed. Our evidence suggests that we might relax such assumption: in our numerical simulations, we observe a similar trend of what we proved even with more structured ansatz like the *quantum approximate optimization algorithm* (QAOA) [92], as demonstrated in Fig. 7. This provides further support for the notion that the assumption regarding the 2-qubit gates being sampled by a 2-design is not crucial.

---

[1] A. A. Mele, Introduction to Haar measure tools in quantum information: A beginner's tutorial (2023), arXiv:2307.08956v2.
[2] D. Gross, K. Audenaert, and J. Eisert, Evenly distributed unitaries: On the structure of unitary designs, J. Math. Phys. **48**, 052104 (2007).
[3] D. Gottesman, The Heisenberg representation of quantum computers (1998), arXiv:quant-ph/9807006.
[4] S. Aaronson and D. Gottesman, Improved simulation of stabilizer circuits, Phys. Rev. A **70**, 052328 (2004).
[5] Z. Webb, The Clifford group forms a unitary 3-design (2016), arXiv:1510.02769.
[6] H. Zhu, R. Kueng, M. Grassl, and D. Gross, The Clifford group fails gracefully to be a unitary 4-design (2016), arXiv:1609.08172.
[7] R. Bhatia, *Positive definite matrices* (Princeton University Press, Princeton, 2007).
[8] C. King and M. Ruskai, Minimal entropy of states emerging from noisy quantum channels, IEEE Trans. Inf. Th. **47**, 192–209 (2001).
[9] M. Beth Ruskai, S. Szarek, and E. Werner, An analysis of completely-positive trace-preserving maps on M2, Lin. Alg. Appl. **347**, 159 (2002).
[10] L. D. Landau and L. M. Lifshitz, *Quantum mechanics non-relativistic theory, third edition: Volume 3*, 3rd ed. (Butterworth-Heinemann, 1981).
[11] M. Schumann, F. K. Wilhelm, and A. Ciani, Emergence of noise-induced barren plateaus in arbitrary layered noise models, Quantum Sci. Technol. **9**, 045019 (2024).

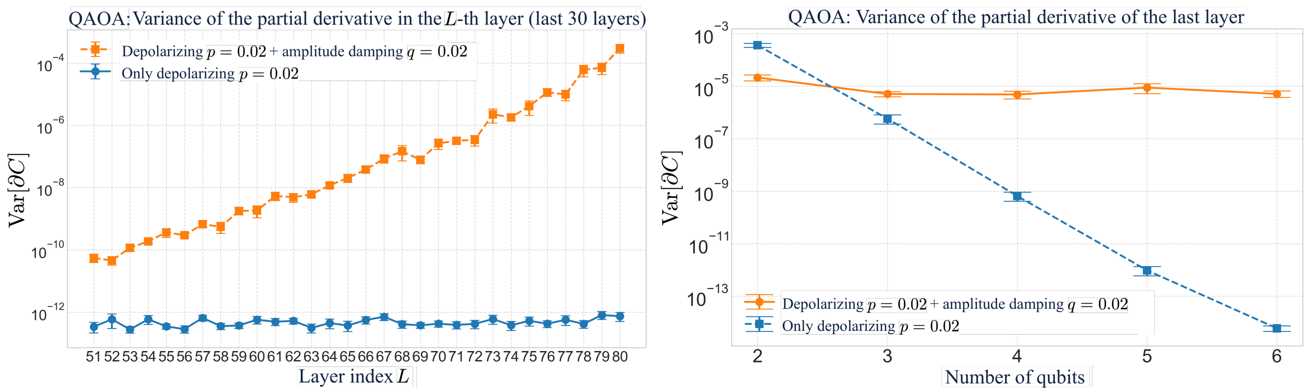

Figure 7. The same quantities as in Fig. 6, but using a QAOA circuit ansatz. The results show no qualitative change, confirming that our conclusions are not limited to unstructured random ansätze. The noise parameters are $p = q = 0.02$, and all circuit parameters $\{\gamma_i, \beta_i\}$ are sampled uniformly in $[0, 2\pi)$. As an observable, we use X_1 instead of Z_1 (the latter has vanishing expectation value in the noiseless case for any fixed QAOA circuit due to symmetry). The QAOA ansatz takes the form $\prod_{i=1}^{D} \exp(-i\beta_i H_x) \exp(-i\gamma_i H_z) \left|+\right\rangle^{\otimes n}$, with $H_z = \sum_{i=1}^{n-1} Z_i Z_{i+1}$ and $H_x = \sum_{i=1}^{n} X_i$. Error bars represent the standard deviation over 20 random parameter samples. Error bars indicate the standard error of the variance estimate.

[12] Y. Quek, D. S. França, S. Khatri, J. J. Meyer, and J. Eisert, Exponentially tighter bounds on limitations of quantum error mitigation (2023), arXiv:2210.11505.

[13] O. Fawzi, A. Müller-Hermes, and A. Shayeghi, A lower bound on the space overhead of fault-tolerant quantum computation, in *13th Innovations in Theoretical Computer Science Conference (ITCS 2022)* (Schloss Dagstuhl – Leibniz-Zentrum für Informatik, 2022).

[14] M. Ben-Or, D. Gottesman, and A. Hassidim, Quantum refrigerator (2013), arXiv:1301.1995.

[15] D. Stilck França and R. García-Patrón, Limitations of optimization algorithms on noisy quantum devices, Nature Phys. **17**, 1221–1227 (2021).

[16] G. De Palma, M. Marvian, D. Trevisan, and S. Lloyd, The quantum Wasserstein distance of order 1, IEEE Trans. Inf. Th. **67**, 6627 (2021).

[17] S. Bravyi, A. Kliesch, R. Koenig, and E. Tang, Obstacles to variational quantum optimization from symmetry protection, Phys. Rev. Lett. **125**, 260505 (2020).

[18] E. Farhi, D. Gamarnik, and S. Gutmann, The quantum approximate optimization algorithm needs to see the whole graph: Worst case examples (2020), arXiv:2005.08747.

[19] E. Farhi, D. Gamarnik, and S. Gutmann, The quantum approximate optimization algorithm needs to see the whole graph: A typical case (2020), arXiv:2004.09002.

[20] A. Anshu and T. Metger, Concentration bounds for quantum states and limitations on the QAOA from polynomial approximations, Quantum **7**, 999 (2023).

[21] S. Bravyi, D. Gosset, and R. König, Quantum advantage with shallow circuits, Science **362**, 308–311 (2018).

[22] S. Bravyi, D. Gosset, R. König, and M. Tomamichel, Quantum advantage with noisy shallow circuits, Nature Phys. **16**, 1040–1045 (2020).

[23] A. B. Watts and N. Parham, Unconditional quantum advantage for sampling with shallow circuits (2024), arXiv:2301.00995.

[24] M. Cerezo, A. Arrasmith, R. Babbush, S. C. Benjamin, S. Endo, K. Fujii, J. R. McClean, K. Mitarai, X. Yuan, L. Cincio, and P. J. Coles, Variational quantum algorithms, Nature Rev. Phys. **3**, 625 (2021).

[25] J. R. McClean, S. Boixo, V. N. Smelyanskiy, R. Babbush, and H. Neven, Barren plateaus in quantum neural network training landscapes, Nature Comm. **9**, 4812 (2018).

[26] Z. Holmes, K. Sharma, M. Cerezo, and P. J. Coles, Connecting ansatz expressibility to gradient magnitudes and barren plateaus, PRX Quantum **3**, 010313 (2022).

[27] F. G. S. L. Brandão, A. W. Harrow, and M. Horodecki, Local random quantum circuits are approximate polynomial-designs, Commun. Math. Phys. **346**, 397 (2016).

[28] M. Cerezo, A. Sone, T. Volkoff, L. Cincio, and P. J. Coles, Cost function dependent barren plateaus in shallow parametrized quantum circuits, Nature Comm. **12**, 1791 (2021).

[29] A. Uvarov and J. D. Biamonte, On barren plateaus and cost function locality in variational quantum algorithms, J. Phys. A **54**, 245301 (2021).

[30] J. Napp, Quantifying the barren plateau phenomenon for a model of unstructured variational ansätze (2022), arXiv:2203.06174.

[31] E. Grant, L. Wossnig, M. Ostaszewski, and M. Benedetti, An initialization strategy for addressing barren plateaus in parametrized quantum circuits, Quantum **3**, 214 (2019).

[32] S. H. Sack, R. A. Medina, A. A. Michailidis, R. Kueng, and M. Serbyn, Avoiding barren plateaus using classical shadows, PRX Quantum **3**, 020365 (2022).

[33] A. A. Mele, G. B. Mbeng, G. E. Santoro, M. Collura, and P. Torta, Avoiding barren plateaus via transferability of smooth solutions in a hamiltonian variational ansatz, Phys. Rev. A **106**, L060401 (2022).

[34] S. Liu, S.-X. Zhang, S.-K. Jian, and H. Yao, Training variational quantum algorithms with random gate activation, Phys. Rev. Res. **5**,

L032040 (2023).
[35] M. S. Rudolph, J. Miller, D. Motlagh, J. Chen, A. Acharya, and A. Perdomo-Ortiz, Synergy between quantum circuits and tensor networks: Short-cutting the race to practical quantum advantage (2023), arXiv:2208.13673.
[36] N. Jain, B. Coyle, E. Kashefi, and N. Kumar, Graph neural network initialisation of quantum approximate optimisation, Quantum **6**, 861 (2022).
[37] X. Shi and Y. Shang, Avoiding barren plateaus via Gaussian mixture model (2024), arXiv:2402.13501.
[38] L. Schatzki, M. Larocca, Q. T. Nguyen, F. Sauvage, and M. Cerezo, Theoretical guarantees for permutation-equivariant quantum neural networks (2022), arXiv:2210.09974.
[39] K. Zhang, M.-H. Hsieh, L. Liu, and D. Tao, Toward trainability of quantum neural networks (2020), arXiv:2011.06258.
[40] T. Volkoff and P. J. Coles, Large gradients via correlation in random parameterized quantum circuits, Quant. Sc. Tech. **6**, 025008 (2021).
[41] A. Pesah, M. Cerezo, S. Wang, T. Volkoff, A. T. Sornborger, and P. J. Coles, Absence of barren plateaus in quantum convolutional neural networks, Phys. Rev. X **11**, 041011 (2021).
[42] X. Liu, G. Liu, J. Huang, H.-K. Zhang, and X. Wang, Mitigating barren plateaus of variational quantum eigensolvers (2022), arXiv:2205.13539.
[43] J. J. Meyer, M. Mularski, E. Gil-Fuster, A. A. Mele, F. Arzani, A. Wilms, and J. Eisert, Exploiting symmetry in variational quantum machine learning, PRX Quantum **4**, 010328 (2023).
[44] C.-Y. Park and N. Killoran, Hamiltonian variational ansatz without barren plateaus (2023), arXiv:2302.08529.
[45] M. Larocca, F. Sauvage, F. M. Sbahi, G. Verdon, P. J. Coles, and M. Cerezo, Group-invariant quantum machine learning, PRX Quantum **3**, 030341 (2022).
[46] K. Zhang, L. Liu, M.-H. Hsieh, and D. Tao, Escaping from the barren plateau via Gaussian initializations in deep variational quantum circuits, Adv. Neur. Inf. Proc. Sys. **35**, 18612 (2022).
[47] C.-Y. Park, M. Kang, and J. Huh, Hardware-efficient ansatz without barren plateaus in any depth (2024), arXiv:2403.04844.
[48] H.-K. Zhang, S. Liu, and S.-X. Zhang, Absence of barren plateaus in finite local-depth circuits with long-range entanglement, Phys. Rev. Lett. **132**, 150603 (2024).
[49] M. Cerezo, M. Larocca, D. García-Martín, N. L. Diaz, P. Braccia, E. Fontana, M. S. Rudolph, P. Bermejo, A. Ijaz, S. Thanasilp, E. R. Anschuetz, and Z. Holmes, Does provable absence of barren plateaus imply classical simulability? or, why we need to rethink variational quantum computing (2023), arXiv:2312.09121.
[50] S. Wang, E. Fontana, M. Cerezo, K. Sharma, A. Sone, L. Cincio, and P. J. Coles, Noise-induced barren plateaus in variational quantum algorithms, Nature Comm. **12**, 6961 (2021).
[51] A. Sannia, F. Tacchino, I. Tavernelli, G. L. Giorgi, and R. Zambrini, Engineered dissipation to mitigate barren plateaus (2023), arXiv:2310.15037.
[52] D. Aharonov, M. Ben-Or, R. Impagliazzo, and N. Nisan, Limitations of noisy reversible computation (1996), arXiv:quant-ph/9611028.
[53] A. Müller-Hermes, D. Stilck França, and M. M. Wolf, Relative entropy convergence for depolarizing channels, J. Math. Phys. **57**, 022202 (2016).
[54] B. Fefferman, S. Ghosh, M. Gullans, K. Kuroiwa, and K. Sharma, Effect of non-unital noise on random circuit sampling (2023), arXiv:2306.16659.
[55] A. M. Dalzell, N. Hunter-Jones, and F. G. Brandão, Random quantum circuits anticoncentrate in log depth, PRX Quantum **3**, 010333 (2022).
[56] S. Liu, M.-R. Li, S.-X. Zhang, S.-K. Jian, and H. Yao, Universal Kardar-Parisi-Zhang scaling in noisy hybrid quantum circuits, Phys. Rev. B **107**, L201113 (2023).
[57] S. Liu, M.-R. Li, S.-X. Zhang, and S.-K. Jian, Entanglement structure and information protection in noisy hybrid quantum circuits, Phys. Rev. Lett. **132**, 240402 (2024).
[58] S. Liu, M.-R. Li, S.-X. Zhang, S.-K. Jian, and H. Yao, Noise-induced phase transitions in hybrid quantum circuits (2024), arXiv:2401.16631.
[59] P. Singkanipa and D. A. Lidar, Beyond unital noise in variational quantum algorithms: noise-induced barren plateaus and limit sets (2024), arXiv:2402.08721.
[60] S. Bravyi, M. B. Hastings, and F. Verstraete, Lieb-Robinson Bounds and the generation of correlations and topological quantum order, Phys. Rev. Lett. **97**, 050401 (2006).
[61] S. Bravyi, D. Gosset, R. König, and M. Tomamichel, Quantum advantage with noisy shallow circuits, Nature Phys. **16**, 1040–1045 (2020).
[62] E. Fontana, M. S. Rudolph, R. Duncan, I. Rungger, and C. Cîrstoiu, Classical simulations of noisy variational quantum circuits (2023), arXiv:2306.05400.
[63] Y. Shao, F. Wei, S. Cheng, and Z. Liu, Simulating noisy variational quantum algorithms: A polynomial approach, Phys. Rev. Lett. **133**, 120603 (2024).
[64] M. Schwarz, O. Buerschaper, and J. Eisert, Approximating local observables on projected entangled pair states, Phys. Rev. A **95**, 060102 (2017).
[65] J. Borregaard, M. Christandl, and D. Stilck França, Noise-robust exploration of many-body quantum states on near-term quantum devices, npj Quant. Inf. **7**, 45 (2021).
[66] D. Aharonov, X. Gao, Z. Landau, Y. Liu, and U. Vazirani, A polynomial-time classical algorithm for noisy random circuit sampling, in *Proc. 55th Ann. ACM Symp. Th. Comp.*, STOC '23 (ACM, 2023).
[67] T. Schuster, C. Yin, X. Gao, and N. Y. Yao, A polynomial-time classical algorithm for noisy quantum circuits (2024), arXiv:2407.12768.
[68] M. M. Wilde, *Quantum information theory* (Cambridge University Press, 2013).
[69] C. Hirche, C. Rouzé, and D. S. França, Quantum differential privacy: An information theory perspective, IEEE Trans. Inf. Th. **69**, 5771 (2023).

[70] A. Angrisani, A. Schmidhuber, M. S. Rudolph, M. Cerezo, Z. Holmes, and H.-Y. Huang, Classically estimating observables of noiseless quantum circuits, Phys. Rev. Lett. **135**, 170602 (2025).
[71] H.-Y. Huang, S. Chen, and J. Preskill, Learning to predict arbitrary quantum processes, (2023), arXiv:2210.14894.
[72] More generally, the same counterexample demonstrates that for $1 \leq p \leq \infty$, the inequality $\|\Phi^\dagger(O)\|_p \leq \|O\|_p$ can hold for all quantum channels Φ and observables O only if $p = \infty$. Specifically, for $p = \infty$, the inequality $\|\Phi^\dagger(O)\|_\infty \leq \|O\|_\infty$ is satisfied for all channels and observables (Russo-Dye Theorem [7]). Furthermore, it can be shown that for $1 \leq p < \infty$, the inequality $\|\Phi^\dagger(O)\|_p \leq \|O\|_p$ holds for all observables O if and only if the channel Φ is unital. This result follows by: 1) the fact shown in [**?**] that for $p > 1$, the inequality $\|\Phi(\rho)\|_p \leq \|\rho\|_p$ holds for all matrices ρ if and only if the channel Φ is unital, 2) from a duality argument, that is, let $p, q \in \mathbb{R}$ be such that $p^{-1} + q^{-1} = 1$, then Φ is p-norm contractive if and only if $\Phi^\dagger$ is q-norm contractive.
[73] A. M. Dalzell, N. Hunter-Jones, and F. G. S. L. Brandão, Random quantum circuits transform local noise into global white noise (2021), arXiv:2111.14907.
[74] A. W. Harrow and S. Mehraban, Approximate unitary t-designs by short random quantum circuits using nearest-neighbor and long-range gates, Commun. Math. Phys. **401**, 1531 (2023).
[75] S. Wang, P. Czarnik, A. Arrasmith, M. Cerezo, L. Cincio, and P. J. Coles, Can error mitigation improve trainability of noisy variational quantum algorithms? (2021), arXiv:2109.01051.
[76] G. E. Crooks, Gradients of parameterized quantum gates using the parameter-shift rule and gate decomposition (2019), arXiv:1905.13311.
[77] A. A. Razborov, An upper bound on the threshold quantum decoherence rate (2003), arXiv:quant-ph/0310136.
[78] J. Kempe, O. Regev, F. Unger, and R. De Wolf, Upper bounds on the noise threshold for fault-tolerant quantum computing, in *International Colloquium on Automata, Languages, and Programming* (Springer, 2008) pp. 845–856.
[79] G. De Palma, M. Marvian, C. Rouzé, and D. S. França, Limitations of variational quantum algorithms: A quantum optimal transport approach, PRX Quantum **4**, 010309 (2023).
[80] C. Hirche, C. Rouzé, and D. S. França, On contraction coefficients, partial orders and approximation of capacities for quantum channels, Quantum **6**, 862 (2022).
[81] M. Müller-Lennert, F. Dupuis, O. Szehr, S. Fehr, and M. Tomamichel, On quantum Rényi entropies: A new generalization and some properties, J. Math. Phys. **54**, 122203 (2013).
[82] M. M. Wilde, A. Winter, and D. Yang, Strong converse for the classical capacity of entanglement-breaking and Hadamard channels via a sandwiched Rényi relative entropy, Comm. Math. Phys. **331**, 593 (2014), 1306.1586.
[83] M. Christandl and A. Müller-Hermes, Relative entropy bounds on quantum, private and repeater capacities, Commun. Math. Phys. **353**, 821 (2017).
[84] R. Rubboli, R. Takagi, and M. Tomamichel, Mixed-state additivity properties of magic monotones based on quantum relative entropies for single-qubit states and beyond (2023), arXiv:2307.08258.
[85] Y. Liu, S. Arunachalam, and K. Temme, A rigorous and robust quantum speed-up in supervised machine learning, Nature Phys. **17**, 1013 (2021).
[86] S. Thanasilp, S. Wang, M. Cerezo, and Z. Holmes, Exponential concentration and untrainability in quantum kernel methods, Nature Comm. **16**, 7907 (2025).
[87] V. Havlíček, A. D. Córcoles, K. Temme, A. W. Harrow, A. Kandala, J. M. Chow, and J. M. Gambetta, Supervised learning with quantum-enhanced feature spaces, Nature **567**, 209 (2019).
[88] M. Schuld, Supervised quantum machine learning models are kernel methods (2021), arXiv:2101.11020.
[89] S. Shalev-Shwartz and S. Ben-David, *Understanding machine learning: From theory to algorithms* (Cambridge university press, 2014).
[90] C. L. Canonne, A short note on an inequality between KL and TV (2022), arXiv:2202.07198.
[91] A. Angrisani, M. Doosti, and E. Kashefi, A unifying framework for differentially private quantum algorithms (2023), arXiv:2307.04733.
[92] E. Farhi, J. Goldstone, and S. Gutmann, A quantum approximate optimization algorithm (2014), arXiv:1411.4028.
